# A shape changing tandem Rh(CNC) catalyst: preparation of bicyclo[4.2.0]octa-1,5,7-trienes from terminal aryl alkynes†
†Electronic supplementary information (ESI) available: Full experimental details, NMR and ESI spectra (PDF), primary NMR data (MNOVA), and optimised geometries (XYZ). CCDC 1970203–1970209. For ESI and crystallographic data in CIF or other electronic format see DOI: 10.1039/c9sc06153c


**DOI:** 10.1039/c9sc06153c

**Published:** 2020-01-22

**Authors:** Caroline M. Storey, Audrius Kalpokas, Matthew R. Gyton, Tobias Krämer, Adrian B. Chaplin

**Affiliations:** a Department of Chemistry , University of Warwick , Coventry CV4 7AL , UK . Email: a.b.chaplin@warwick.ac.uk; b Department of Chemistry , Maynooth University , Maynooth , Co. Kildare , Ireland . Email: tobias.kraemer@mu.ie; c Hamilton Institute , Maynooth University , Maynooth , Co. Kildare , Ireland

## Abstract

The preparation of a range of tetraaryl-substituted bicyclo[4.2.0]octa-1,5,7-trienes using a one-pot procedure starting from terminal aryl alkynes and catalysed by a rhodium(i) complex is reported. This synthesis proceeds by a reaction sequence involving head-to-tail homocoupling of the terminal alkyne and zipper annulation of the resulting *gem*-enyne. The rhodium catalyst employed is notable for the incorporation of a flexible NHC-based pincer ligand, which is suggested to interconvert between *mer*- and *fac*-coordination modes to fulfil the orthogonal mechanistic demands of the two transformations. Evidence for this interesting auto-tandem action of the catalyst is provided by reactions of the precatalyst with model substrates, corroborating proposed intermediates in both component cycles, and norbornadiene, which reversibly captures the change in pincer ligand coordination mode, along with a DFT-based computational analysis.

## Abstract 2

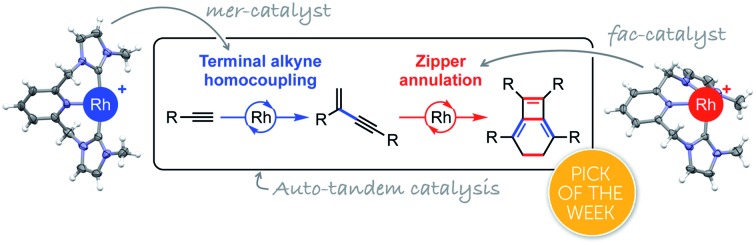
Two catalysts for the price of one: a shape changing rhodium catalyst enables preparation of unusual isobenzenes using a one-pot procedure.

## Introduction

**Fig. 1:**
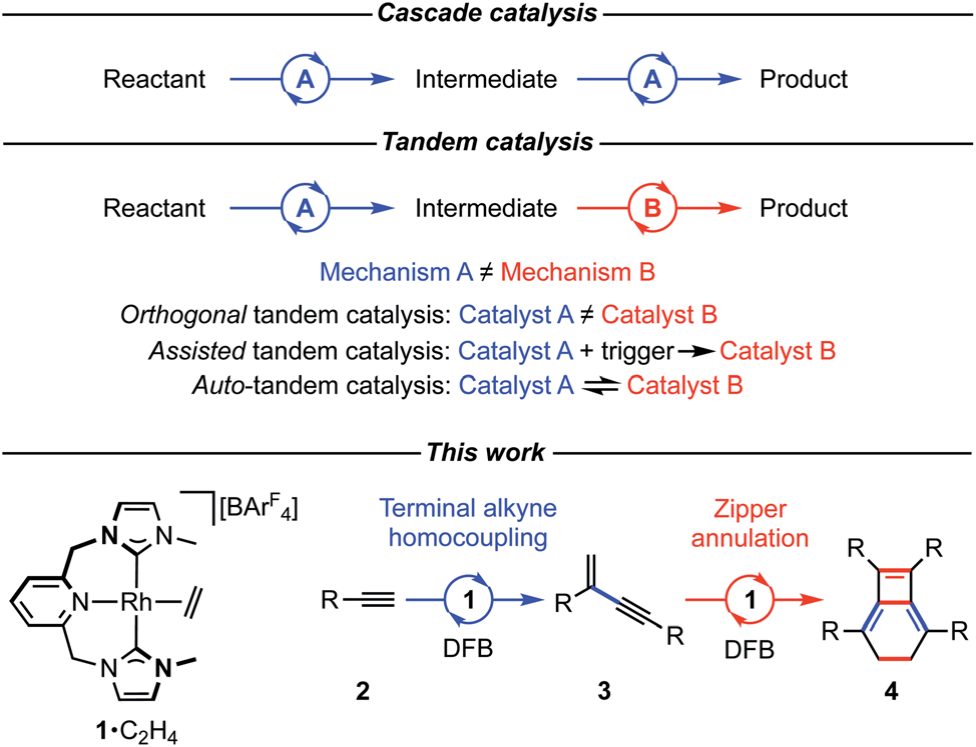
Sequential reaction protocols and bicyclo[4.2.0]octa-1,5,7-triene synthesis.

In pursuit of more efficient synthetic procedures tandem catalysis has emerged as a powerful approach, enabling complex molecules to be assembled in one pot through a precisely choreographed sequence of catalytic steps, reducing waste and saving time (Fig. 1).^1^–^3^ Contrasting cascade or domino reaction manifolds, tandem catalysis involves orchestration of functionally distinct transformations using a single or multiple catalyst precursor(s) (orthogonal tandem catalysis).^2^ The former variation is operationally the simplest and harnesses catalyst productivity most efficiently, however, sequencing multiple operations with high fidelity using a common catalyst precursor can be a formidable challenge. Whilst such temporal control can be accomplished through deliberate intervention to transform the catalyst *in situ* after a suitable time period (assisted tandem catalysis), this solution lacks the practical simplicity of autonomous counterparts (auto-tandem catalysis) that do not require reaction monitoring nor additional experimental operations.^3^

As part of our work exploring terminal alkyne coupling reactions promoted by rhodium complexes of NHC-based pincer ligands,^4^ we serendipitously discovered that [Rh(CNC-Me)(C_2_H_4_)][BAr^F^_4_] (**1**·C_2_H_4_, Ar^F^ = 3,5-(CF_3_)_2_C_6_H_3_) is a highly effective auto-tandem precatalyst for the preparation of bicyclo[4.2.0]octa-1,5,7-trienes from terminal aryl alkynes with high selectivity (**2** → **3** → **4**, Fig. 1). There are only two literature precedents for isobenzenes of this type, with the most pertinent example involving a single-step nickel(0) catalysed annulation of isolated (electron deficient) *gem*-enynes.^5^,^6^ Despite extensive investigation, the selective head-to-tail coupling of terminal alkynes (**2** → **3**) invoked in the formation of **4** remains a challenging task for transition metal catalysts and is typically accompanied with mechanistically interconnected *E*-enynes that are products of head-to-head coupling.^7^ We have, however, previously demonstrated that **1**·C_2_H_4_ is a remarkably regioselective precatalyst for the dimerisation of terminal aryl alkynes (**2a**, R = 3,5-*t*Bu_2_C_6_H_3_=Ar′; **2b**, R = Ph) into the corresponding *gem*-enynes (**3a** and **3b**) under mild conditions in dichloromethane solution.^4^ Upon switching to more weakly coordinating solvent 1,2-F_2_C_6_H_4_ (DFB),^8^ catalyst stability and activity are significantly enhanced with no loss of selectivity, but subsequent metal catalysed conversion into **4** became more apparent. Although metal-catalysed reactions of terminal alkynes have been extensively investigated,^9^ to the best of our knowledge, this one-pot reaction sequence is unprecedented: a paucity that we attribute to the orthogonal mechanistic demands of the component steps.

After briefly overviewing the scope of this unique one-pot reaction, we herein present mechanistic and computational studies that suggest the unique catalytic behaviour of **1** is enabled by the ability of the flexible CNC ligand to adopt both *mer*- and *fac*-coordination modes.^10^ This is a potentially widely applicable concept for tandem catalysis.

## Results and discussion

### Scope of tandem reaction

**Fig. 2:**
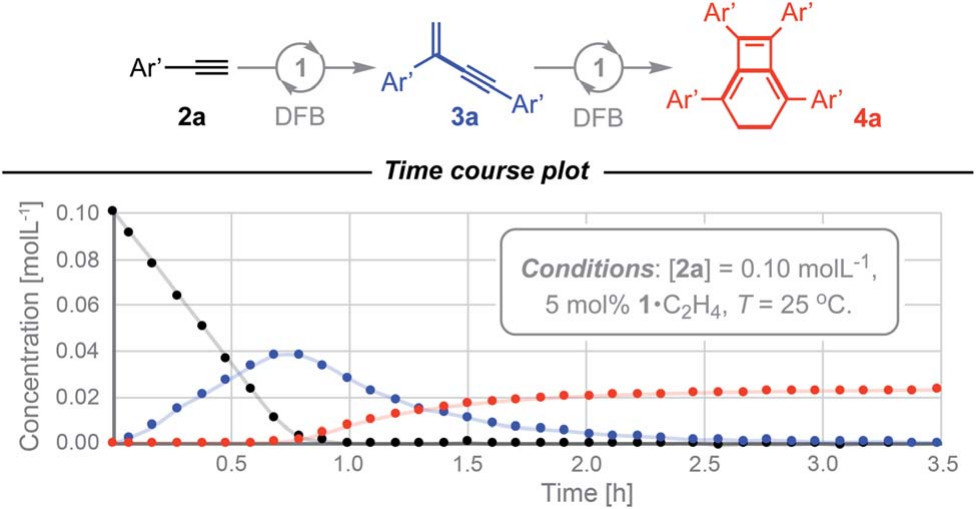
Time course analysis of the formation **4a** from **2a** catalysed by **1**·C_2_H_4_.

We have previously shown the homocoupling of **2a** catalysed by **1**·C_2_H_4_ (5 mol%) in dichloromethane proceeds at 25 °C with a TOF = 2.5 h^–1^ and exclusive formation of the corresponding *gem*-enyne **3a** (until conversion reached *ca.* 90%), which was readily isolated by quenching the reaction at high alkyne conversion with carbon monoxide.^4^ We now report the use of DFB as a solvent, which results in an order of magnitude faster homocoupling under otherwise equivalent conditions (TOF = 26 h^–1^). On the considerably shorter time frames associated with these experiments, the ensuing metal-catalysed reaction of **3a** into balance tetramer **4a** was more readily apparent nearing complete consumption of **2a** by ^1^H NMR spectroscopy, with the appearance of a characteristic 4H singlet resonance at *δ* 2.87 alongside two new 36H *t*Bu resonances at *δ* 1.03 and 1.11 (Fig. 2). Under these conditions, **4a** was obtained in quantitative spectroscopic yield within 4 h. Subsequent isolation and characterisation in solution and the solid state enabled unambiguous assignment of **4a** as a bicyclo[4.2.0]octa-1,5,7-triene and motivated us to explore the scope of this unique sequential reaction.

**Fig. 3:**
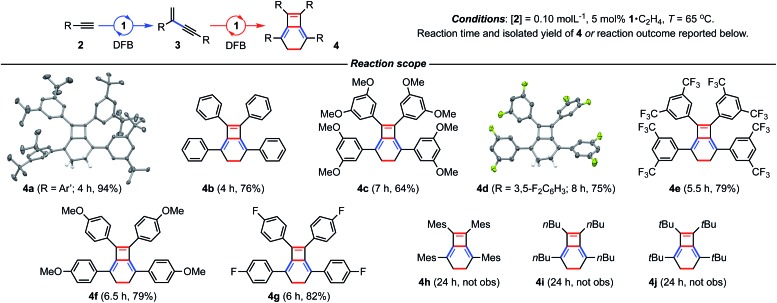
Preparation of bicyclo[4.2.0]octa-1,5,7-trienes from terminal alkynes. Solid-state structures of **4a** (not unique, *Z*′ = 2) and **4d** shown with 50% probability thermal ellipsoids.

After ascertaining the intermediacy of *gem*-enynes, using *in situ* experiments monitored by ^1^H NMR spectroscopy (see ESI†), a straightforward one-pot protocol was developed for the preparation of a range of 2,5,7,8-tetraarylbicyclo-[4.2.0]octa-1,5,7-trienes (Fig. 3). Analytically pure samples of **4a–g** were obtained, with unoptimised yields ranging from 64–94%, and fully characterised. This range of products demonstrates the compatibility of the tandem process for both electron withdrawing and donating groups in the *para* or *meta* positions of the aryl alkyne. Attempts at employing the bulky mesityl substituted alkyne **2h**, however, yielded only an 80 : 20 mixture of *gem*- and *E*-enyne dimerisation products under these conditions. The tandem reaction also appears to be limited to aryl substituted alkynes, with alkyl alkynes **2i** (R = *n*Bu) and **2j** (R = *t*Bu) failing to afford the respective bicyclo[4.2.0]octa-1,5,7-trienes, even on prolonged heating at 65 °C. Homocoupling of these substrates was nevertheless observed, with the latter notable for proceeding with the slow, but exclusive, formation of *E-t*BuC≡CCHCH*t*Bu at 25 °C. Monitoring this reaction *in situ* using ^1^H NMR spectroscopy suggests catalytic turnover is impeded by a significant degree of product inhibition. Indeed, supporting this assertion the corresponding rhodium *E*-enyne complex [Rh(CNC-Me)(*E-t*BuC≡CCHCH*t*Bu)][BAr^F^_4_] **5** observed *in situ* was independently synthesised and fully characterised (see ESI†).

### Mechanistic proposal and supporting organometallic chemistry

**Fig. 4:**
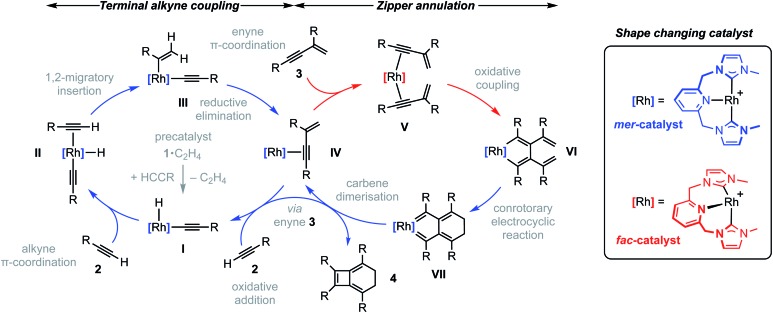
Proposed auto-tandem catalysed preparation of bicyclo[4.2.0]octa-1,5,7-trienes from terminal alkynes.

To reconcile the formation of **4** the auto-tandem scheme outlined in Fig. 4 is hypothesised, which involves asynchronous homocoupling of **2** into **3** followed by annulation of **3** into **4**: the latter enabled by the capacity of CNC-Me to shuttle between *mer*- and *fac*-coordination modes.

A hydrometallation mechanism, which comprises C–H bond oxidative addition of the terminal alkyne (**1**/**IV** → **I**), selectivity determining 1,2-migratory insertion of the second alkyne into the resulting metal hydride (**II** → **III**), and rate determining C–C bond reductive elimination to generate the *gem*-enyne (**III** → **IV**), is proposed for the homocoupling based on literature precedents and our previous interrogation of the **1**/**2a** system.^4^,^7^ Pincer complexes have been shown to be effective catalysts for terminal alkyne coupling reactions and these component steps are fully compatible with *mer*-coordination of CNC-Me throughout the cycle.^11^ Indeed, we have previously shown that terminal alkyne coupling of **2a** by an analogue of **1**, bearing instead a macrocyclic NHC-based pincer ligand [Rh(CNC-12)(C_2_H_4_)][BAr^F^_4_] **6**·C_2_H_4_, results exclusively in entrapment of the enyne product within the annulus of the ligand.^4^ This outcome is only possible if the NHC-based pincer ligand maintains a *mer*-coordination mode during the homocoupling.
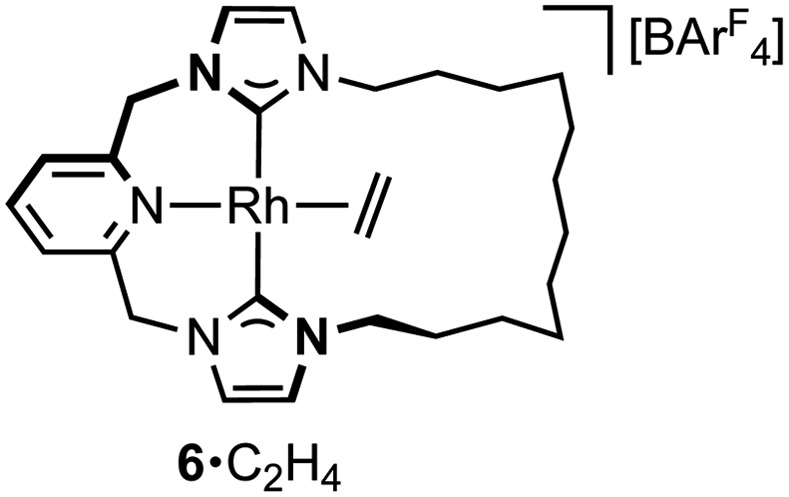


**Fig. 5:**
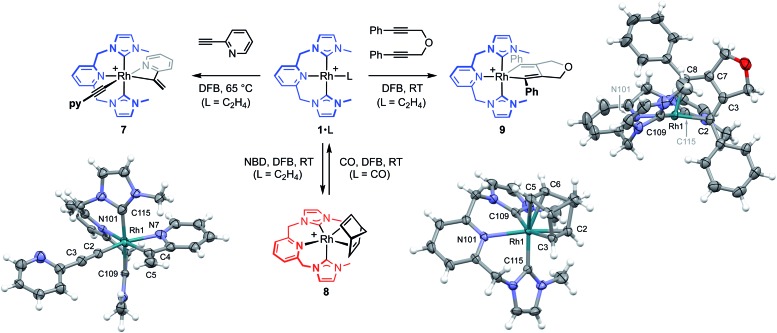
Reactivity of **1**·C_2_H_4_ relevant to the proposed mechanism ([BAr^F^_4_]^–^ anions omitted). Solid-state structures of the cations of **7**, **8** and **9** (not unique, *Z*′ = 2) shown with 30%, 50% and 50% probability thermal ellipsoids, respectively. Selected data: **7**, Rh1–C2, 1.92(2) Å; C2–C3, 1.21(2) Å; Rh1–C2–C3, 179(2)°; Rh1–C4, 2.02(2) Å; C4–C5, 1.30(3) Å; Rh1–N7, 2.15(2) Å; Rh1–C4–C5, 140(2)°; Rh1–N101, 2.25(2) Å; Rh1–C109, 2.01(2) Å; Rh1–C115, 2.01(2) Å; C109–Rh1–C115, 174.5(8)°; **8**, Rh1–Cnt(C2,C3), 1.964(2) Å; Rh1–Cnt(C5,C6), 2.144(2) Å; C2–C3, 1.431(4) Å; C5–C6, 1.369(4) Å; N101–Rh1–Cnt(C2,C3), 135.48(8)°; C109–Rh1–Cnt(C2,C3), 140.01(9)°; C115–Rh1–Cnt(C5,C6), 160.86(10)°; Rh1–N101, 2.327(2) Å; Rh1–C109, 2.118(3) Å; Rh1–C115, 2.026(2) Å; C109–Rh1–C115, 103.60(10)°; **9**, Rh1–C2, 2.029(2) Å; Rh1–C8, 2.024(2) Å, N101–Rh1–C2, 171.69(8)°; C2–C3, 1.354(3) Å; C3–C7, 1.433(3) Å; C7–C8, 1.345(3) Å; Rh1–N101, 2.242(2) Å; Rh1–C109, 2.049(2) Å; Rh1–C115, 2.063(2) Å; C109–Rh1–C115, 171.92(8)°.

To help provide further evidence for the hydrometallation mechanism, the reaction between **1**·C_2_H_4_ and 2-ethynylpyridine was studied in DFB (Fig. 5). Heating at 65 °C overnight resulted in exclusive formation of Rh(iii) *gem*-alkenyl alkynyl **7** (*cf.***III**),^4^,^12^ where chelation of the pyridine completes the coordination sphere and encumbers onward reductive elimination. Complex **7** was subsequently isolated from solution in 82% yield and fully characterised, including structural elucidation in the solid state using single crystal X-ray diffraction (1.2 Å resolution). Key spectroscopic features of **7** include *C*_1_ symmetry, geminal alkene ^1^H resonances at *δ* 5.97 and 6.48, and ^13^C resonances at *δ* 98.8 (RhC[combining low line]≡C, ^1^*J*_RhC_ = 56 Hz) and 137.0 (RhC[combining low line](CH_2_), ^1^*J*_RhC_ = 25 Hz) that display large ^103^Rh coupling.

Although the annulation of **3** has little direct precedent, parallels can be drawn with oxidative coupling reactions of internal alkynes, which afford metallacyclopentadienes and, in some cases, thereafter the corresponding cyclobutadiene complexes.^13^ In these well-established reactions, *cis*-parallel coordination of the alkynes is a prerequisite for efficient mixing of the alkyne frontier molecular orbitals,^14^ and correspondingly the majority of examples are associated with *fac*-coordinating ancillary ligands (*e.g.* cyclopentadienyls) that promote these geometries. On this basis we suggest the annulation of **3** commences with distortion of the catalyst geometry in such a way as to place the CNC-Me ligand in a *fac*-coordination mode and therefore enable binding of two enynes in a *cis*-parallel arrangement (**IV** → **V**). Subsequent oxidative coupling would then afford metallacyclopentadiene **VI**, with the alkyne carbon atom bearing the most sterically demanding substituents forming the σ-bond with the metal centre in accordance with Wakatsuki's rule.^14^ A metal promoted conrotatory electrocyclic reaction (**VI** → **VII**) and product elimination, involving formal alkylidene dimerisation, is thereafter proposed to afford **4**. The latter step is related, by the principle of microscopic reversibility, to the insertion of metals into tetraaminoethylenes for which there is precedent for rhodium(i).^15^ An alternative product releasing route, where formation of the four-membered ring of the isobenzene precedes that of the six, through formation of the corresponding cyclobutadiene complex of **VI** and then electrocyclic reaction, was also considered but ultimately discounted (*vide infra*).

The key requirement of the annulation conjecture is the ability of the CNC ligand to adopt a *fac*-coordination mode and this was confirmed by reaction of **1**·C_2_H_4_ with 1 equivalent of norbornadiene (NBD) in DFB at RT, which resulted in quantitative formation of **8** within 3 h in a sealed tube (Fig. 5, 76% isolated yield). Such behaviour is uncharacteristic for pincer ligands, but some examples can be found in the literature.^16^ The formation of **8** was established by NMR spectroscopy and X-ray diffraction. In solution, **8** is notable is for the adoption of time averaged *C*_s_ symmetry of the pincer and fast rotation of the NBD ligand on the NMR time scale (298 K, 500 MHz), carbene resonances at *δ* 187.4 that show enhanced ^1^*J*_RhC_ coupling compared to **1**·C_2_H_4_ (51 *vs.* 40 Hz), and an alkene ^13^C signal at *δ* 43.6 (^1^*J*_RhC_ = 8 Hz). In the solid state the metal adopts a distorted trigonal bipyramidal geometry (C109–Rh1–C115 = 103.60(10)°), with the appreciably shorter axial Rh–NHC (2.026(2) *vs.* 2.118(3) Å) and equatorial Rh–alkene (1.964(2) *vs.* 2.144(2) Å) contacts in line with a structurally related complexes.^17^ Reaction of isolated **8** with carbon monoxide (1 atm) generated the known square planar carbonyl derivative of **1** within 5 h at RT.^4^ Macrocyclic **6**·C_2_H_4_ also reacts reversibly with NBD and, in strong support of the underlying hypotheses, catalyses the formation of **4a** from **3a**, albeit under considerably more forcing reaction conditions than its acyclic congener (TON = 2, after 45 days at 50 °C; details provided in the ESI†).

We next turned to probe the capacity for **1** to promote the oxidative coupling of two alkynes, for which di(3-phenylprop-2-ynyl)ether was identified from the literature.^18^ Gratifyingly, reaction between **1**·C_2_H_4_ and the propargyl ether in DFB at RT afforded five-coordinate metallacyclopentadiene **9** within 30 min, as marked visually by its characteristic dark green colour (Fig. 5). Complex **9** was subsequently isolated in 97% yield and fully characterised. In solution **9** displays time averaged *C*_2_ symmetry (298 K, 500 MHz), with the metallacyclopentadiene ^13^C resonances located at *δ* 150.8 (RhC[combining low line](Ph)C, ^1^*J*_RhC_ = 41 Hz) and 155.5 (RhC(Ph)C[combining low line], ^2^*J*_RhC_ = 3 Hz). Five-coordinate **9** adopts a distorted square pyramidal structure in the solid state, with the pincer ligand in the expected *mer*-coordination mode. We have previously reported a 2,2′-biphenyl complex of **6**, which shows a similar geometry and the metal-based metrics are in good agreement.^19^ As a structural analogue of **VI**, we sought to ascertain if the corresponding cyclobutadiene complex could be obtained. As no reaction was evident on extended thermolysis of **9** in DFB (85 °C, 24 h) we discount this possibility.

### Computational evaluation

**Fig. 6:**
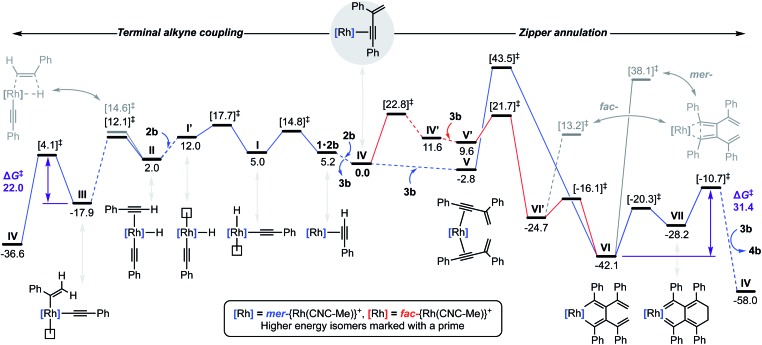
Calculated reaction profile (B3PW91-D3/SDD/6-31G**) for the terminal alkyne coupling of **2b** and zipper annulation of **3b**. Relative Gibbs free energies (kcal mol^–1^) are corrected for DFB solvent. Solid traces for elementary steps for which transition states have been calculated.

To substantiate the proposed auto-tandem reaction and help elucidate the mechanistic intricacies, DFT calculations at the B3PW91-D3/SDD/6-31G** level of theory were employed for the most computationally amenable phenyl-substituted system (Fig. 6). Coordination and C(sp)–H bond oxidative addition of **2b** proceeds with a low barrier, but endergonic isomerisation (Δ*G* = +7.0 kcal mol^–1^) of the resulting alkynyl hydride **I** is required to bind the second equivalent *trans* to the alkynyl (**II**).^20^ The two possible regioisomers of **II** are nearly isoenergetic, but it is only for the head-to-tail configuration that 1,2-migratory insertion is productive with respect to dissociation. As the reaction thereafter proceeds thermodynamically downhill, this step is irreversible and selectively determining (ΔΔ*G*^‡^ = –2.5 kcal mol^–1^).^21^ Consistent with the experimental findings, *gem*-alkenyl alkynyl **III** is calculated to be the resting state. Subsequent reductive elimination affording **IV** is turnover limiting (Δ*G*^‡^ = 22.0 kcal mol^–1^) and completes the production of **3b**, which is formed in Δ*G* = –36.6 kcal mol^–1^ overall.

Two pathways for the annulation have been computationally evaluated: the lowest energy route commences with ring flipping of one of the bridging methylene groups of the pincer backbone,^22^ which distorts the metal coordination geometry and ultimately causes CNC-Me to adopt a *fac*-coordination mode (**IV′**). This process is associated with a small thermodynamic penalty of Δ*G* = +11.6 kcal mol^–1^, but appreciable barrier of Δ*G*^‡^ = 22.8 kcal mol^–1^. The latter is consistent with the highly asynchronous nature of the tandem reaction, with the homocoupling proceeding with a lower barrier of Δ*G*^‡^ = 17.7 kcal mol^–1^ with respect to **IV**. The *fac*-configuration is stabilised by coordination of an additional equivalent of **3b**, forming **V′** and from which facile and irreversible oxidative coupling is possible: Δ*G*^‡^ = 21.7 kcal mol^–1^ relative to **IV**, but only 12.1 kcal mol^–1^ with respect to **V′**. Formation of a cyclobutadiene complex from the resulting *fac*-metallacyclopentadiene **VI′** was examined, but the associated barrier is considerably higher than formation of the corresponding and thermodynamically preferred *mer*-isomer **VI** (Δ*G* = –42.1 kcal mol^–1^). The alternative higher energy annulation pathway involves retention of CNC-Me in a *mer*-coordination mode and converges at **VI**, but is associated with a prohibitively high activation barrier of Δ*G*^‡^ = 43.5 kcal mol^–1^ for the oxidative coupling and is consequently discounted. In line with the experimentally established stability of **9**, subsequent C–C bond reductive elimination from **VI** is prohibitively high in energy (Δ*G*^‡^ = 55.3/80.2 kcal mol^–1^), however the postulated conrotatory electrocyclic reaction (**VI** → **VII**) and alkylidene dimerisation (**VII** → **IV**) appears energetically feasible. The latter is predicted to be turnover limiting in the case of the phenyl-substituted system (Δ*G*^‡^ = +31.4 kcal mol^–1^), producing **4b** in Δ*G* = –58.0 kcal mol^–1^ overall.^23^

## Conclusions

An atom efficient and operationally simple procedure for the synthesis of unusual bicyclic isobenzenes from terminal alkynes is reported. Using this one-pot protocol seven novel tetraaryl-substituted bicyclo[4.2.0]octa-1,5,7-trienes were successfully prepared, with the aryl substituents bearing a range of electron withdrawing and donating groups in the *para* or *meta* positions.

This synthesis proceeds by a reaction sequence involving head-to-tail homocoupling of the terminal alkyne and annulation of the resulting *gem*-enyne. Both are catalysed with remarkably high fidelity by a common rhodium(i) catalyst, which features a flexible NHC-based pincer ligand that is hypothesised to interconvert between *mer*- and *fac*-coordination modes to fulfil the orthogonal mechanistic demands of the two transformations. Experimental evidence for this interesting auto-tandem action of the catalyst is provided by reactions of the precatalyst with model substrates, corroborating the formation of *gem*-alkenyl alkynyl and metallacyclopentadiene intermediates in the homocoupling and annulation steps respectively, and norbornadiene, which reversibly captures the change in the pincer ligand coordination mode. This work is supplemented by a detailed DFT-based computational analysis, which supports a hydrometallation-based homocoupling and a zipper-type annulation reaction that proceeds by oxidative coupling of the two *gem*-enynes, metal promoted conrotatory electrocyclic reaction and a product releasing formal alkylidene dimerisation.

The capacity of the NHC-based pincer ligand to adopt both *mer*- and *fac*-coordination modes appears to be central to the success of this one-pot procedure and this concept may prove to be a fruitful for the design of new tandem catalytic reactions.

## Conflicts of interest

There are no conflicts to declare.

## Supplementary Material

Supplementary information

Supplementary information

Supplementary information

Crystal structure data
