# Supplementary material for: A shape changing tandem Rh(CNC) catalyst: preparation of bicyclo[4.2.0]octa-1,5,7-trienes from terminal aryl alkynes
Source: Chem Sci. 2020 Jan 22;11(8):2051–7. doi: 10.1039/c9sc06153c (PMC7053420; doi:10.1039/c9sc06153c)

Electronic Supplementary Material (ESI) for Chemical Science.
This journal is © The Royal Society of Chemistry 2020

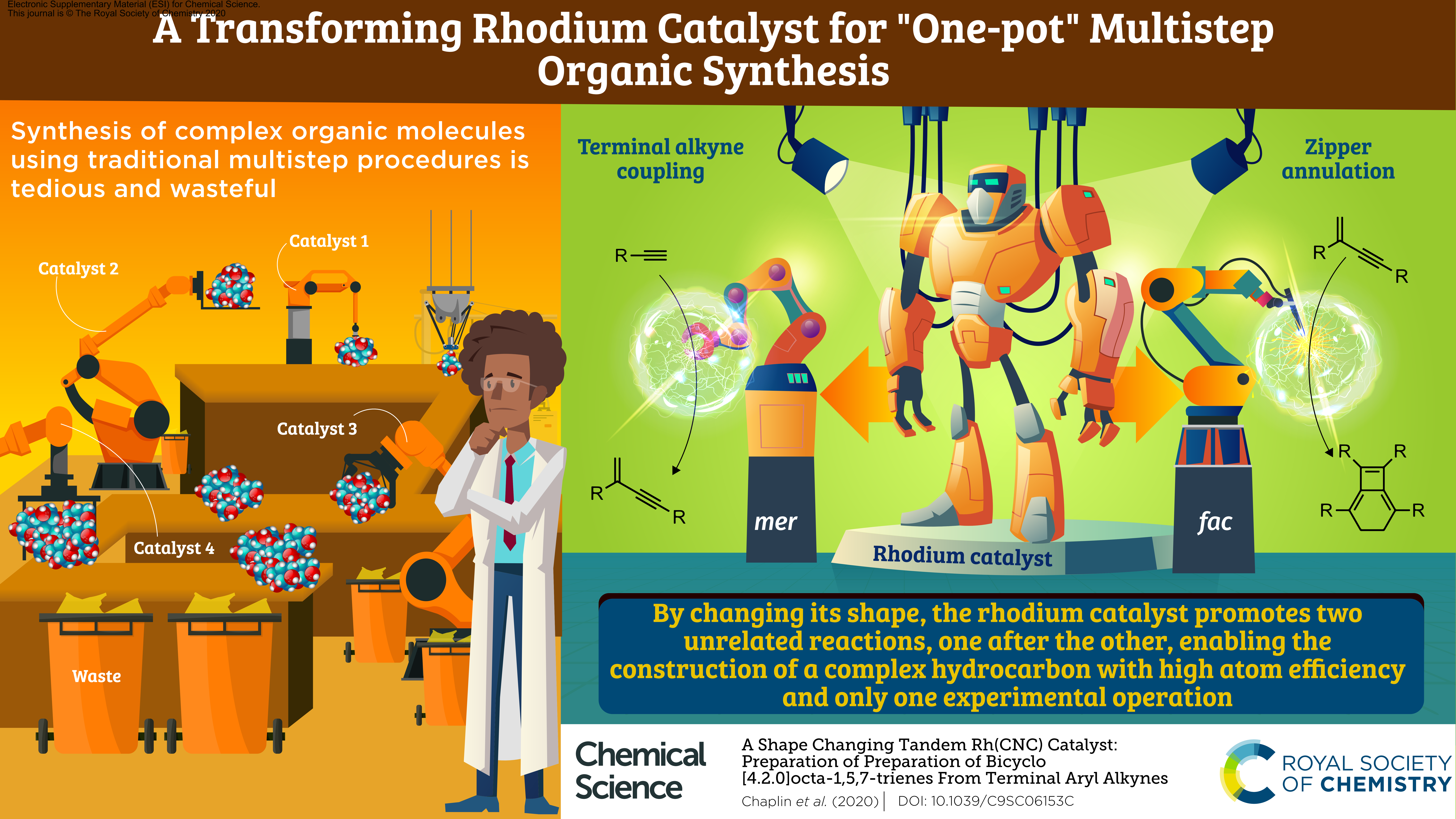

Supplement: Supplementary file 2 [file SC-011-C9SC06153C-s002.pdf]
