# Supplementary material for: A shape changing tandem Rh(CNC) catalyst: preparation of bicyclo[4.2.0]octa-1,5,7-trienes from terminal aryl alkynes
Source: Chem Sci. 2020 Jan 22;11(8):2051–7. doi: 10.1039/c9sc06153c (PMC7053420; doi:10.1039/c9sc06153c)
Supplement: Supplementary file 3 [file SC-011-C9SC06153C-s003.pdf]

Electronic Supplementary Material (ESI) for Chemical Science.
This journal is © The Royal Society of Chemistry 2020

Electronic Supporting information for

A shape changing tandem Rh(CNC) catalyst: preparation of bicyclo[4.2.0]octa-1,5,7-trienes from terminal aryl alkynes

Caroline M. Storey,[a] Audrius Kalpokas,[b] Matthew R. Gyton,[a] Tobias Krämer,*[bc] and Adrian B. Chaplin*[a]

[a] *Department of Chemistry, University of Warwick, Coventry CV4 7AL, UK.*

[b] *Department of Chemistry, Maynooth University, Maynooth, Co. Kildare, Ireland.*

[c] *Hamilton Institute, Maynooth University, Maynooth, Co. Kildare, Ireland.*

Table of contents

1. General experimental methods 2
2. Reaction scope 3
3. NMR scale reaction of **1**·C_2H_4 with 2-ethynylpyridine 30
4. Synthesis of [Rh(CNC-Me)(C≡Cpy){pyC(CH_2)}][BAr^F_4] **7** 30
5. NMR scale reaction of **1**·C_2H_4 with NBD 33
6. Synthesis of [Rh(CNC-Me)(NBD)][BAr^F_4] **8** 33
7. NMR scale reaction of **8** with CO 35
8. NMR scale reaction of **1**·C_2H_4 with di(3-phenylprop-2-ynyl)ether 36
9. Synthesis of [Rh(CNC-Me)($Ph_2C_6H_4O$)][BAr^F_4] **9** 36
10. NMR scale thermolysis of **9** in DFB 38
11. Organometallic chemistry of [Rh(CNC-12)(C_2H_4)][BAr^F_4] **6**·C_2H_4 39
12. Computational methods 43
13. Homocoupling of PhC≡CH (**2b**) 44
14. Homocoupling of *t*BuC≡CH (**2j**) 49
15. Annulation of PhC≡CC(CH_2)Ph (**3b**) 52
16. References 55

1. General experimental methods

All manipulations were performed under an atmosphere of argon using Schlenk and glove box techniques. Glassware was oven-dried at 150 °C overnight and flame-dried under vacuum prior to use. Molecular sieves were activated by heating at 300 °C *in vacuo* overnight. Fluorobenzene (FB) and 1,2-difluorobenzene (DFB) were stirred over alumina, distilled from calcium hydride and dried twice over 3 Å molecular sieves.[1] Hexane was stored over a potassium mirror. Tetramethylsilane was dried over liquid Na/K alloy, vacuum distilled and stored over a potassium mirror. CD_2Cl_2 was freeze–pump–thaw degassed and dried over activated 3 Å molecular sieves. Anhydrous CH_2Cl_2, diethyl ether, and pentane were purchased from Acros or Sigma-Aldrich and freeze–pump–thaw degassed and stored over activated 3 Å molecular sieves. [Rh(CNC-Me)(C_2H_4)][BAr$^F_4$] (**1**·C_2H_4),[2] [Rh(CNC-12)(C_2H_4)][BAr$^F_4$] (**6**·C_2H_4),[2] 3,5-di-*tert*-butylphenylacetylene (Ar'C≡CH),[3] *E*-*t*BuC≡CCHCH*t*Bu,[4] di(3-phenylprop-2-ynyl)ether,[5] and Ar'C≡CC(CH_2)Ar'[2] were synthesised using literature protocols. All other solvents and reagents are commercial products and were used as received. NMR spectra were recorded on Bruker spectrometers at 298 K unless otherwise stated. NMR spectra in DFB were recorded using an internal capillary of C_6D_6.[1] Chemical shifts are quoted in ppm and coupling constants in Hz. High resolution (HR) ESI-MS were recorded on Bruker Maxis Plus instrument. Microanalyses were performed by Stephen Boyer at London Metropolitan University.

2. Reaction scope

2.1. General procedure: preparation of bicyclo[4.2.0]octa-1,5,7-trienes

In an argon-filled glovebox, a standard solution (5 mL, 100 mM) of terminal alkyne (**2**) in DFB was added to a J. Young's flask charged with **1**·C_2H_4 (31.5 mg, 0.025 mmol) and the resulting solution stirred at 65 °C. After a given time (Table S1), informed from proceeding *in situ* experiments or periodic sampling of the reaction to monitor progress using ^1H NMR spectroscopy, the solution was freeze-pump-thaw degassed and placed under an atmosphere of CO (1 atm).

Table S1. Reaction times and isolated yields of bicyclo[4.2.0]octa-1,5,7-trienes **4**.

R	Alkyne	Bicyclo[4.2.0]octa-1,5,7-triene	Reaction time / h	Isolated yield of 4
3,5-tBu$_2$C$_6$H$_3$	**2a**	**4a**	4 h	94%
Ph	**2b**	**4b**	4 h	76%
3,5-(MeO)$_2$C$_6$H$_3$	**2c**	**4c**	7 h	64%
3,5-F$_2$C$_6$H$_3$	**2d**	**4d**	8 h	75%
3,5-(CF$_3$)$_2$C$_6$H$_3$	**2e**	**4e**	5.5 h	79%
4-(MeO)C$_6$H$_4$	**2f**	**4f**	6.5 h	79%
4-FC$_6$H$_4$	**2g**	**4g**	6 h	82%
Mes	**2h**	Not formed	24 h	-
*n*Bu	**2i**	Not formed	24 h	-
*t*Bu	**2j**	Not formed	24 h	-

2.2. Synthesis of 4a

2.2.1. *In situ* experiments

The formation of **4a** by terminal alkyne coupling of alkyne **2a** into *gem*-enyne **3a** and subsequent annulation, catalysed by **1**·C_2H_4, was established using a reaction carried out in a J. Young's valve NMR tube (100 mM **2a**, 5 mol% **1**·C_2H_4, 0.5 mL DFB) that was monitored *in situ* using ^{1}H NMR spectroscopy at 25 °C. Intermediate formation of **3a** is apparent by CH_2 signals at δ 5.93 and 5.67, which are in good agreement with literature data.[2]

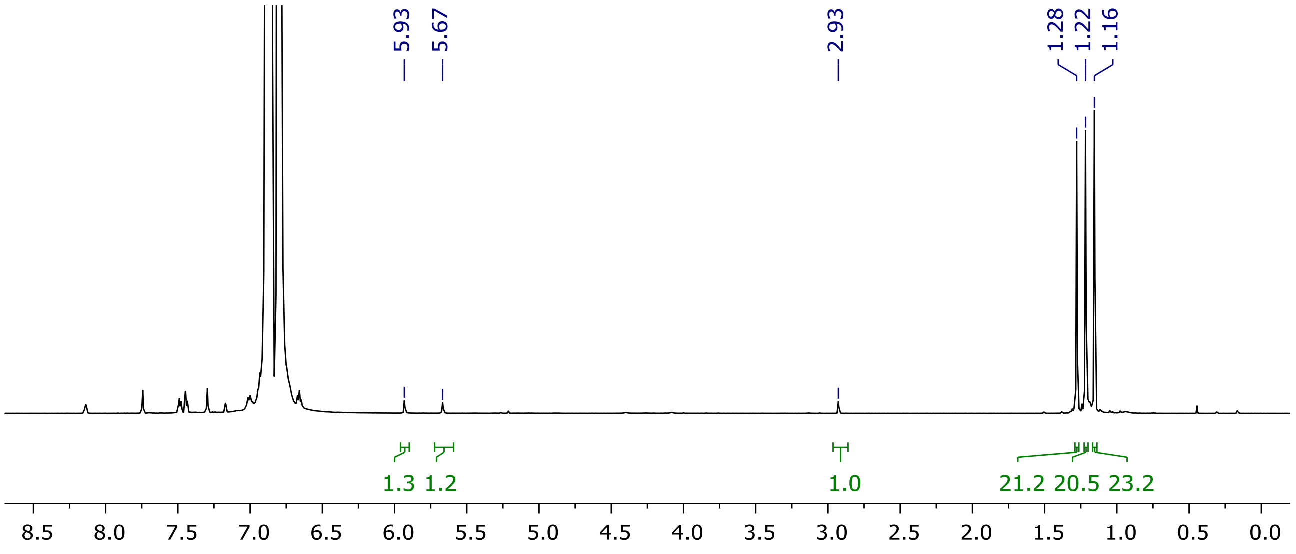

Figure S1: ^{1}H NMR spectrum collected during the synthesis of **4a** at 25 °C (*t* = 0.5 h, DFB, 600 MHz).

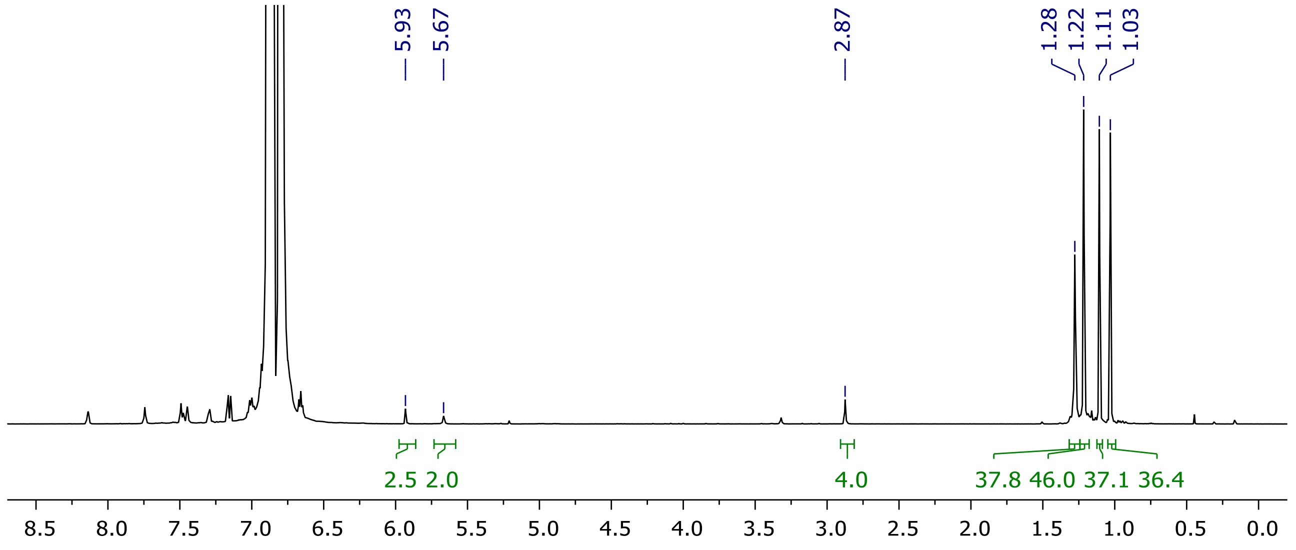

Figure S2: ^{1}H NMR spectrum collected during the synthesis of **4a** at 25 °C (*t* = 1.1 h, DFB, 600 MHz).

2.2.2. Preparation of 4a

Bicyclo[4.2.0]octa-1,5,7-triene **4a** was isolated following the general procedure, quenching after 4 h. The resulting solution was reduced to dryness *in vacuo* and the product extracted into cold tetramethylsilane

(0 °C). The product was isolated as a pale-yellow solid on removal of volatiles under reduced pressure. Yield: 101 mg (94%). Crystals suitable for X-ray diffraction were grown by slow evaporation of a solution of the product in tetramethylsilane.

^{1}H NMR (600 MHz, DFB, selected data): *δ* 2.87 (s, 4H, CH_2), 1.11 (s, 36H, *t*Bu), 1.03 (s, 36H, *t*Bu).

^{1}H NMR (500 MHz, CD_2Cl_2): *δ* 7.22 (t, $^4J_{HH}$ = 1.9, 2H, *p*-CH), 7.18 (t, $^4J_{HH}$ = 1.9, 2H, *p*-CH), 7.05 (d, $^4J_{HH}$ = 1.9, 4H, *o*-CH), 7.02 (d, $^4J_{HH}$ = 1.9, 4H, *o*-CH), 2.97 (s, 4H, CH_2), 1.11 (s, 36H, *t*Bu), 1.08 (s, 36H, *t*Bu).

^{13}C{^{1}H} NMR (126 MHz, CD_2Cl_2): *δ* 150.5 (s, *m*-C), 150.3 (s, *m*-C), 149.0 (s, $C^{7/8}$), 139.2 (s, *i*-C), 135.5 (s, $C^{1/6}$), 133.0 (s, *i*-C), 123.5 (s, *o*-CH), 123.0 (s, *o*-CH), 122.9 (s, *p*-CH), 121.3 (s, *p*-CH), 118.7 (s, $C^{2/5}$), 35.1 (s, *t*Bu{C}), 35.0 (s, *t*Bu{C}), 31.6 (s, 2× *t*Bu{CH_3}), 30.0 (s, $C^{3/4}H_2$).

HR ESI-MS (positive ion, 4 kV): 879.6775 ([*M*+Na]$^+$, calcd 879.6778) *m/z*.

Anal. Calcd for $C_{32}H_{24}$ (857.41 g·mol^{-1}): C, 89.65; H, 10.35; N, 0.00. Found: C, 89.61; H, 10.35; N, 0.00.

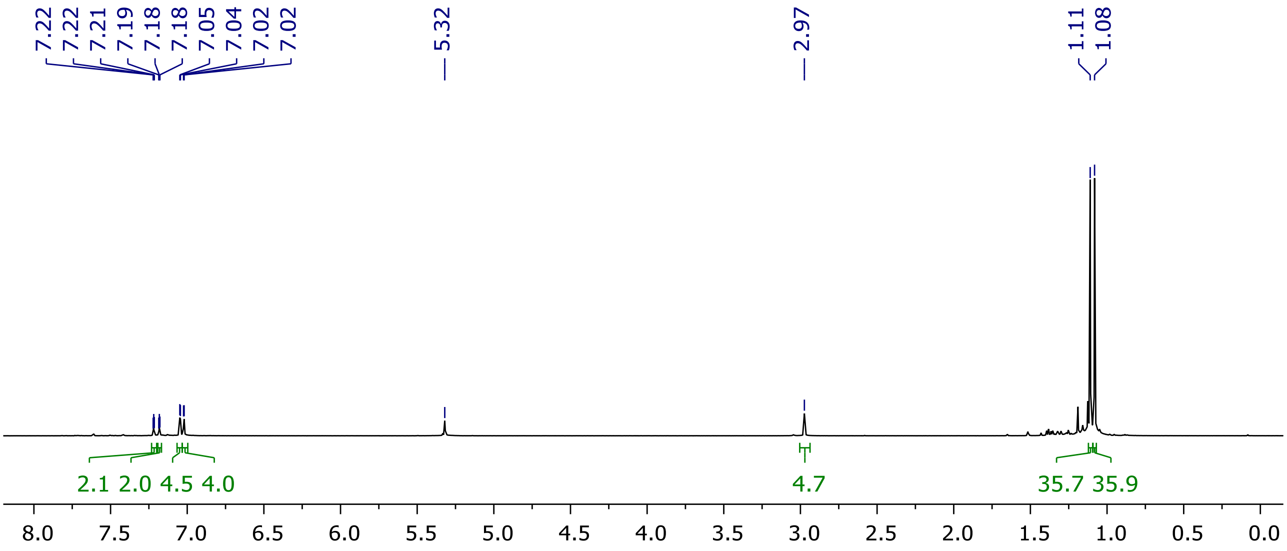

Figure S3: ^{1}H NMR spectrum of **4a** (CD_2Cl_2, 500 MHz).

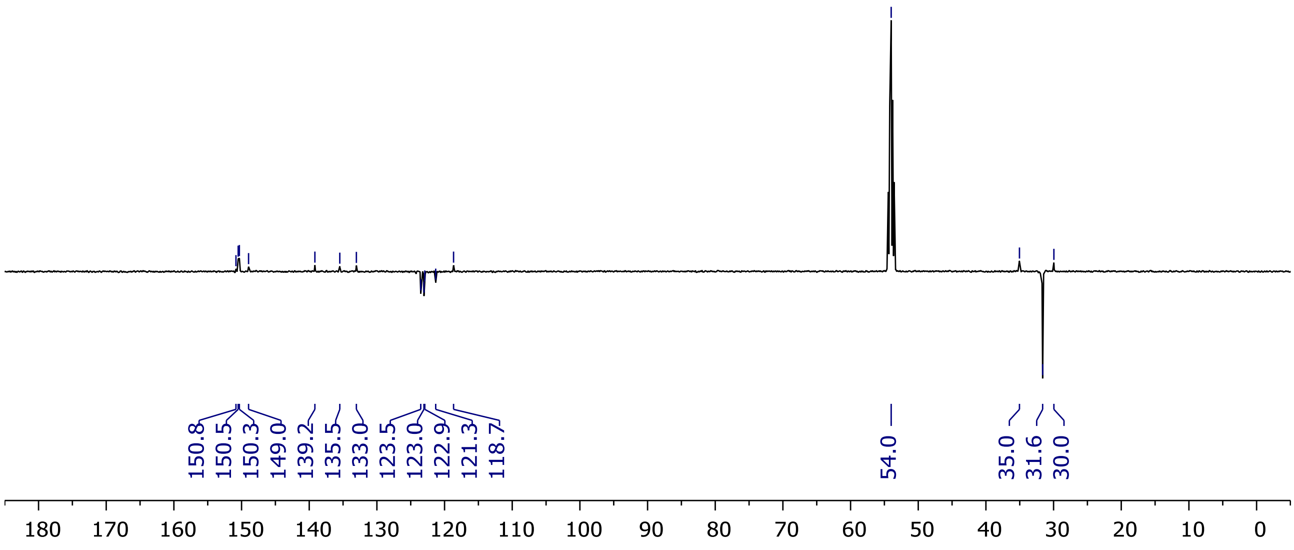

Figure S4: ^{13}C{^{1}H} APT NMR spectrum of **4a** (CD_2Cl_2, 126 MHz).

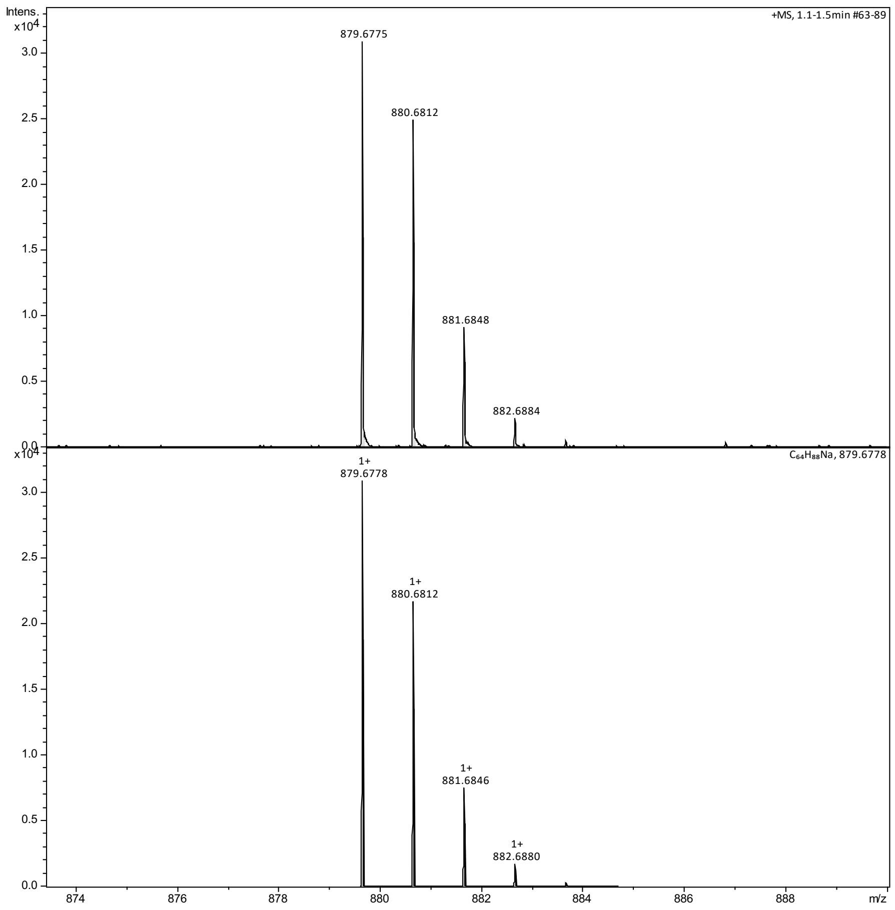

Figure S5: ESI-MS of **4a**.

2.3. Synthesis of 4b

2.3.1. *In situ* experiment

The formation of **4b** by terminal alkyne coupling of alkyne **2b** into *gem*-enyne **3b** and subsequent annulation, catalysed by **1**·C_2H_4, was established using a reaction carried out in a J. Young's valve NMR tube (100 mM **2b**, 5 mol% **1**·C_2H_4, 0.5 mL DFB) that was monitored *in situ* using ^1H NMR spectroscopy at 25 °C. Intermediate formation of **3b** is apparent by CH_2 signals at δ 5.82 and 5.61, which are in good agreement with literature data.[6]

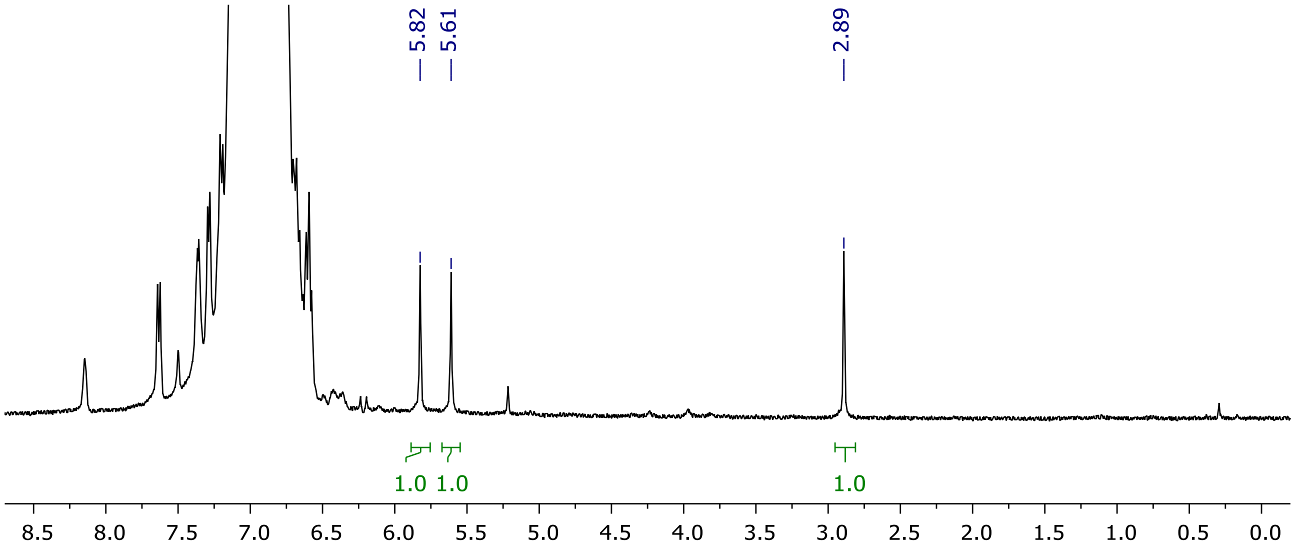

Figure S6: ^1H NMR spectrum collected during the synthesis of **4b** at 25 °C (*t* = 1.7 h, DFB, 400 MHz).

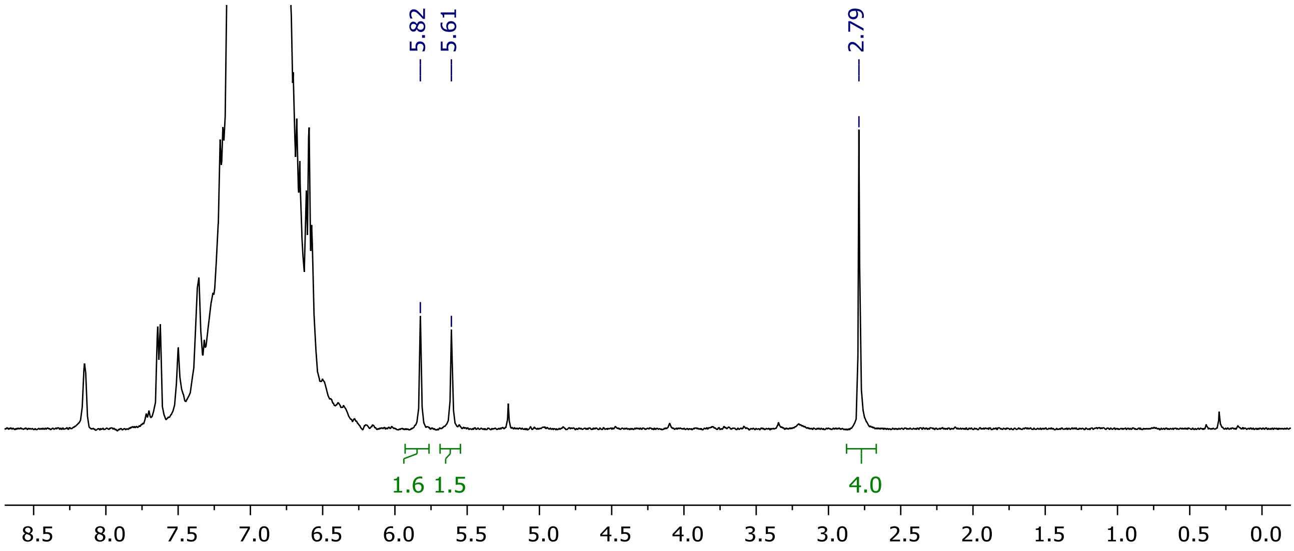

Figure S7: ^1H NMR spectrum collected during the synthesis of **4b** at 25 °C (*t* = 7 h, DFB, 400 MHz).

2.3.2. Preparation of 4b

Bicyclo[4.2.0]octa-1,5,7-triene **4b** was isolated following the general procedure, quenching after 4 h. The resulting solution was reduced to dryness *in vacuo* and the product extracted into hot hexane (50 °C). The solution was concentrated and cooled to 4 °C to afford the product as pale-yellow needles. Yield: 39 mg (76%).

^{1}H NMR (400 MHz, DFB, selected data): *δ* 2.79 (s, 4H, CH_2).

^{1}H NMR (500 MHz, CD_2Cl_2): δ 7.09 – 7.27 (m, 20H, CH), 2.98 (s, 4H, CH_2).

^{13}C{^{1}H} NMR (126 MHz, CD_2Cl_2): δ 148.7 (s, $C^{7/8}$), 139.9 (s, *i*-C), 135.6 (s, $C^{1/6}$), 133.6 (s, *i*-C), 129.2 (s, CH), 128.8 (s, CH), 128.6 (s, *p*-CH), 128.4 (s, CH), 128.2 (s, CH), 127.3 (s, *p*-CH), 118.7 (s, $C^{2/5}$), 29.5 (s, $C^{3/4}H_2$).

HR ESI-MS (positive ion, 4 kV): 447.1511 ([*M*+K]$^+$, calcd 447.1510) *m/z*.

Anal. Calcd for $C_{32}H_{24}$ (408.54 g·mol^{-1}): C, 94.08; H, 5.92; N, 0.00. Found: C, 94.08; H, 5.84; N, 0.00.

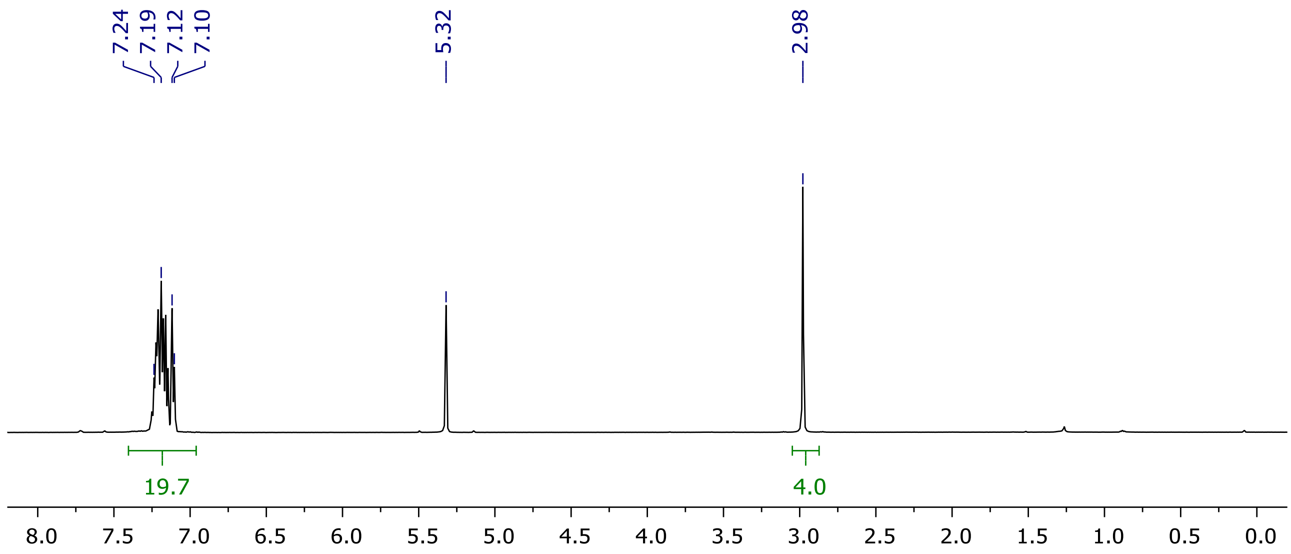

Figure S8: ^{1}H NMR spectrum of **4b** (CD_2Cl_2, 500 MHz).

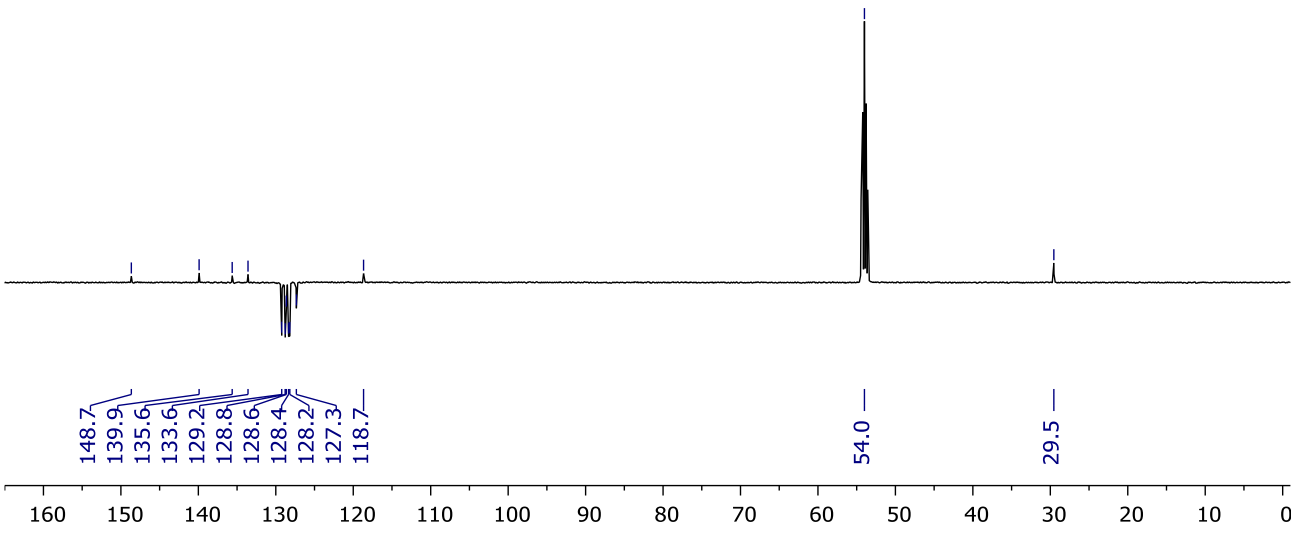

Figure S9: ^{13}C{^{1}H} APT NMR spectrum of **4b** (CD_2Cl_2, 126 MHz).

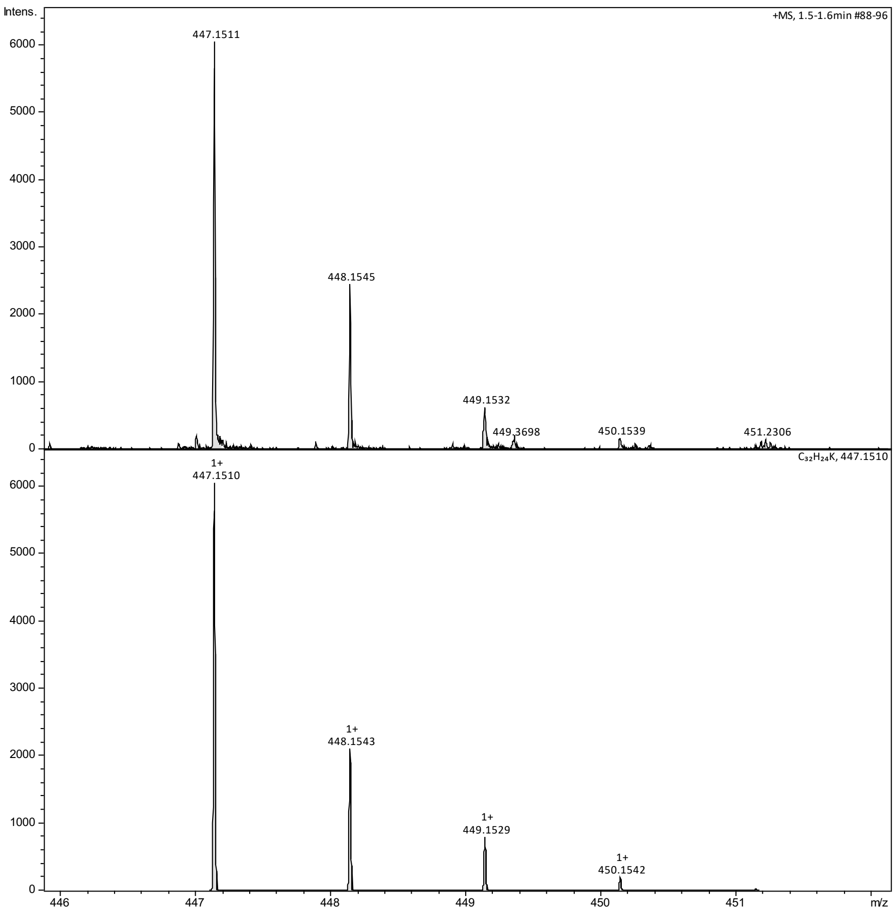

Figure S10: ESI-MS of **4b**.

2.4. Synthesis of 4c

2.4.1. *In situ* experiment

The intermediate formation of **3c** during the **1**·C_2H_4 catalysed formation of **4c** from **2c** was established using a reaction carried out in a J. Young's valve NMR tube (100 mM **2c**, 5 mol% **1**·C_2H_4, 0.5 mL DFB) that was periodically monitored *in situ* using ^{1}H NMR spectroscopy at RT. Formation of **3c** was apparent by CH_2 signals at δ 5.87 and 5.64.

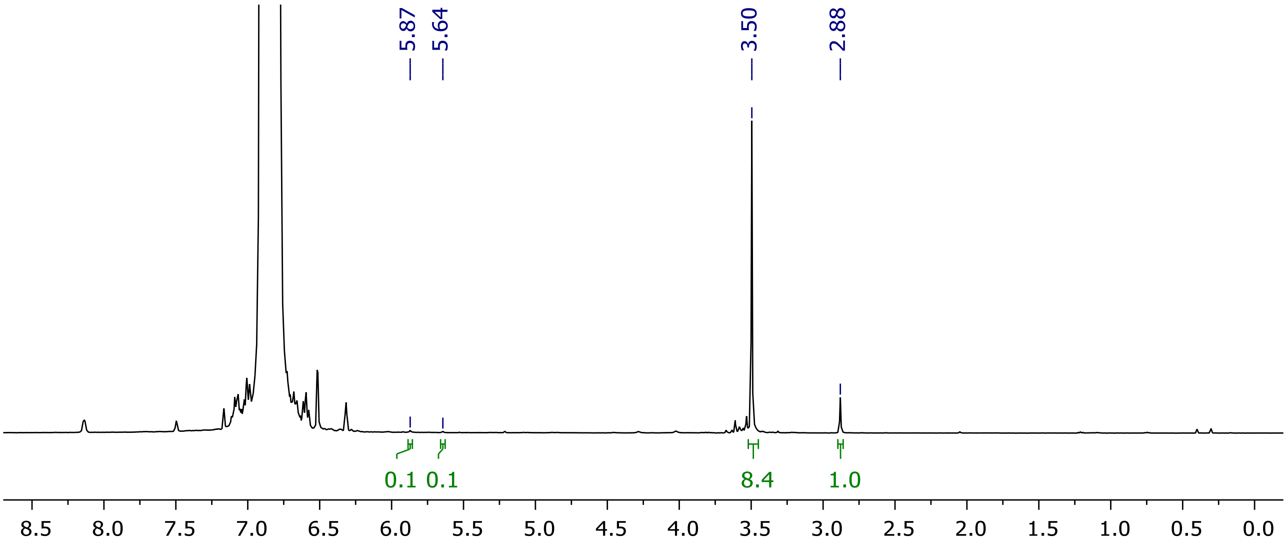

Figure S11: ^{1}H NMR spectrum collected during the synthesis of **4c** at RT (t < 1 h, DFB, 400 MHz).

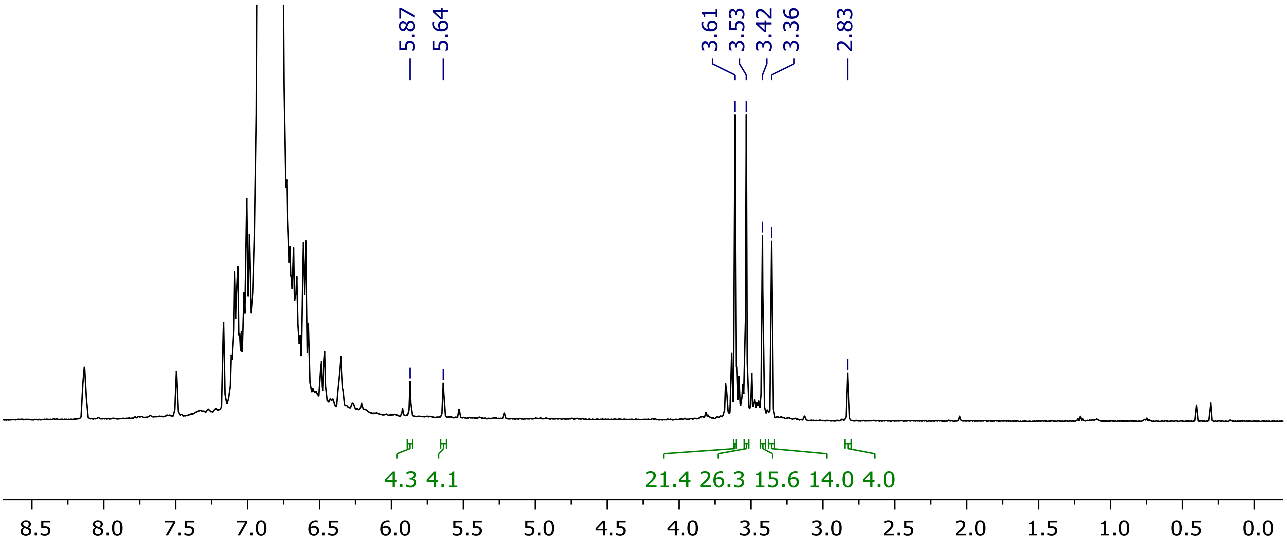

Figure S12: ^{1}H NMR spectrum collected during the synthesis of **4c** at RT (t = 14.5 h, DFB, 400 MHz).

2.4.2. Preparation of 4c

Bicyclo[4.2.0]octa-1,5,7-triene **4c** was isolated following the general procedure, quenching after 7 h. The dark yellow solution was concentrated to dryness and the residue washed with cold diethyl ether (0 °C) to afford the pure product as a pale yellow solid. Yield: 53 mg (64%).

^{1}H NMR (400 MHz, DFB, selected data): *δ* 3.42 (s, 12H, CH_3), 3.36 (s, 12H, CH_3), 2.83 (s, 4H, CH_2).

^{1}H NMR (500 MHz, CD_2Cl_2): *δ* 6.40 (d, $^4J_{HH}$ = 2.2, 4H, *o*-CH), 6.37 (d, $^4J_{HH}$ = 2.2, 4H, *o*-CH), 6.35 (t, $^4J_{HH}$ = 2.2, 2H, *p*-CH), 6.32 (t, $^4J_{HH}$ = 2.2, 2H, *p*-CH), 3.52 (s, 12H, CH_3), 3.49 (s, 12H, CH_3), 2.94 (s, 4H, CH_2).

^{13}C{^{1}H} NMR (126 MHz, CD_2Cl_2): δ 160.9 (s, *m*-C), 160.7 (s, *m*-C), 148.9 (s, $C^{7/8}$), 141.7 (s, *i*-C), 135.5 (s, $C^{1/6}$), 134.9 (s, *i*-C), 119.2 (s, $C^{2/5}$), 106.9 (s, *o*-CH), 106.8 (s, *o*-CH), 101.9 (s, *p*-CH), 100.3 (s, *p*-CH), 55.7 (s, CH_3), 55.5 (s, CH_3), 29.5 (s, $C^{3/4}H_2$).

HR ESI-MS (positive ion, 4 kV): 671.2607 ([*M*+Na]$^+$, calcd 671.2615) *m/z*.

Anal. Calcd for $C_{40}H_{40}O_8$ (648.75 g·mol^{-1}): C, 74.06; H, 6.22; N, 0.00. Found: C, 73.82; H, 6.30; N, 0.00.

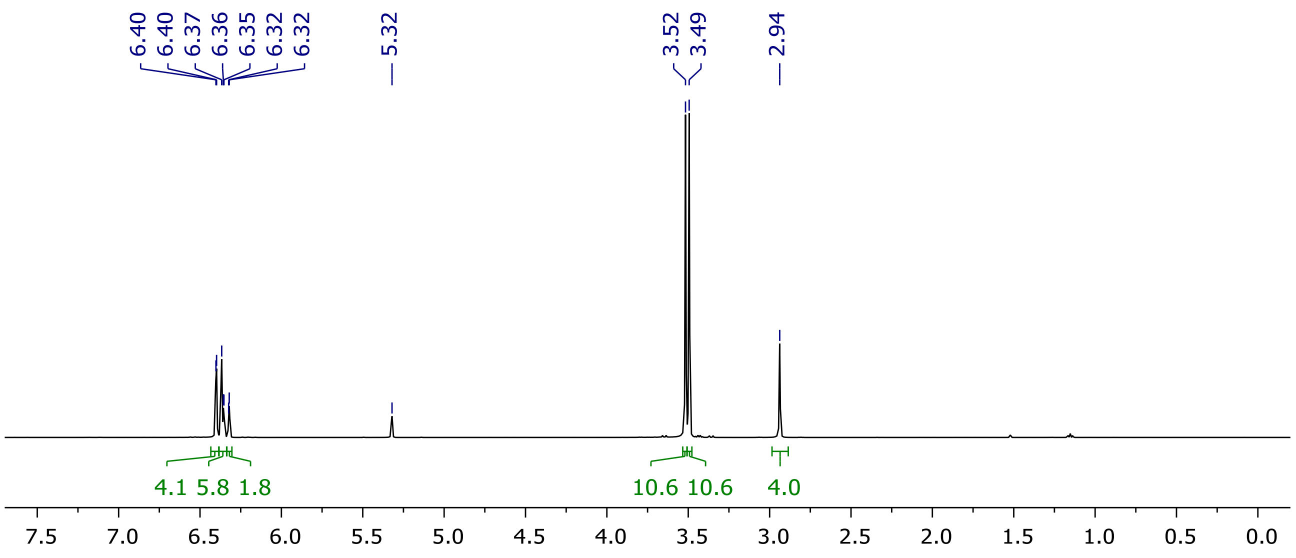

Figure S13: ^{1}H NMR spectrum of **4c** (CD_2Cl_2, 500 MHz).

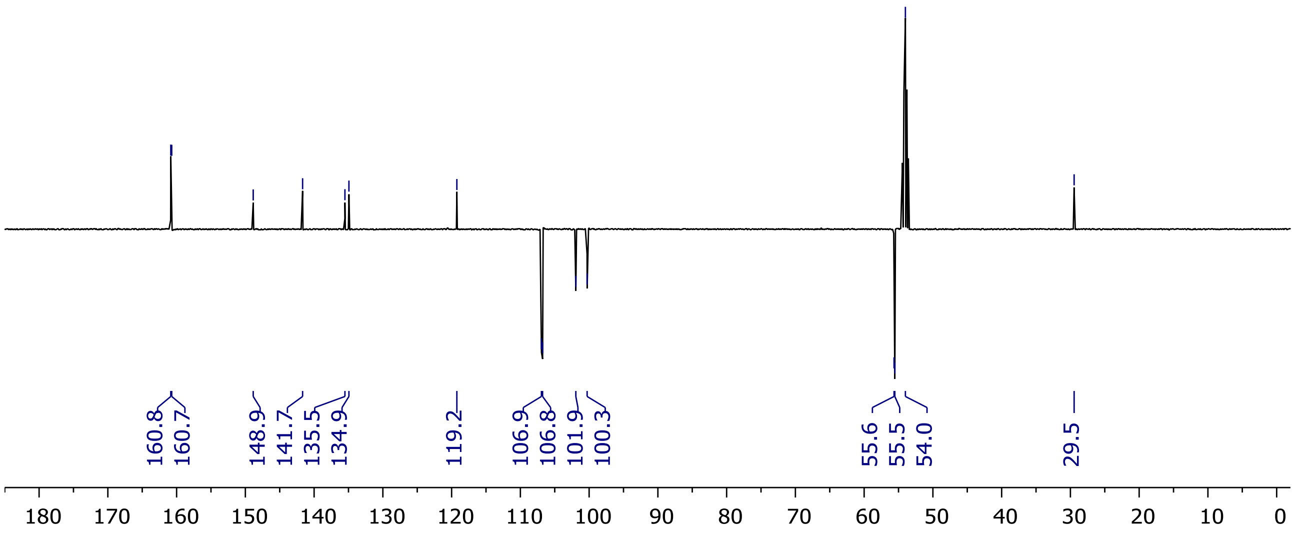

Figure S14: ^{13}C{^{1}H} APT NMR spectrum of **4c** (CD_2Cl_2, 126 MHz).

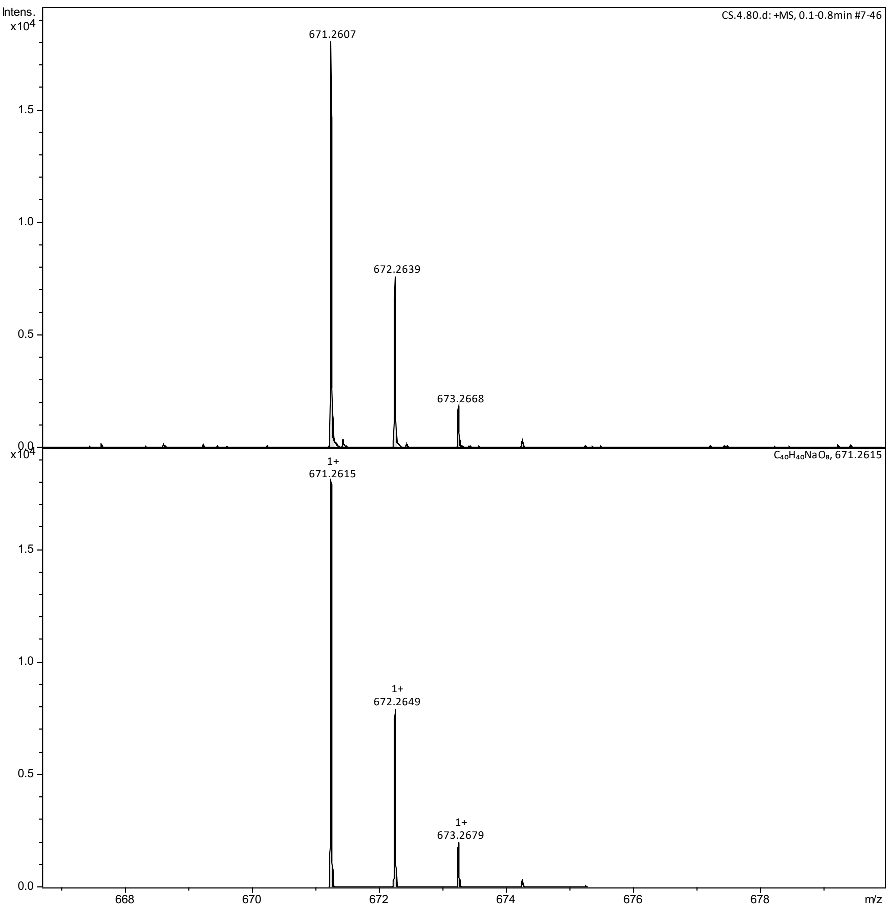

Figure S15: ESI-MS of **4c**.

2.5. Synthesis of 4d

2.5.1. *In situ* experiment

The intermediate formation of **3d** during the **1**·C_2H_4 catalysed formation of **4d** from **2d** was established using a reaction carried out in a J. Young's valve NMR tube (50 mM **2d**, 5 mol% **1**·C_2H_4, 0.5 mL DFB) that was periodically monitored *in situ* using ^{1}H NMR spectroscopy at RT. Formation of **3d** was apparent by CH_2 signals at δ 5.81 and 5.66.

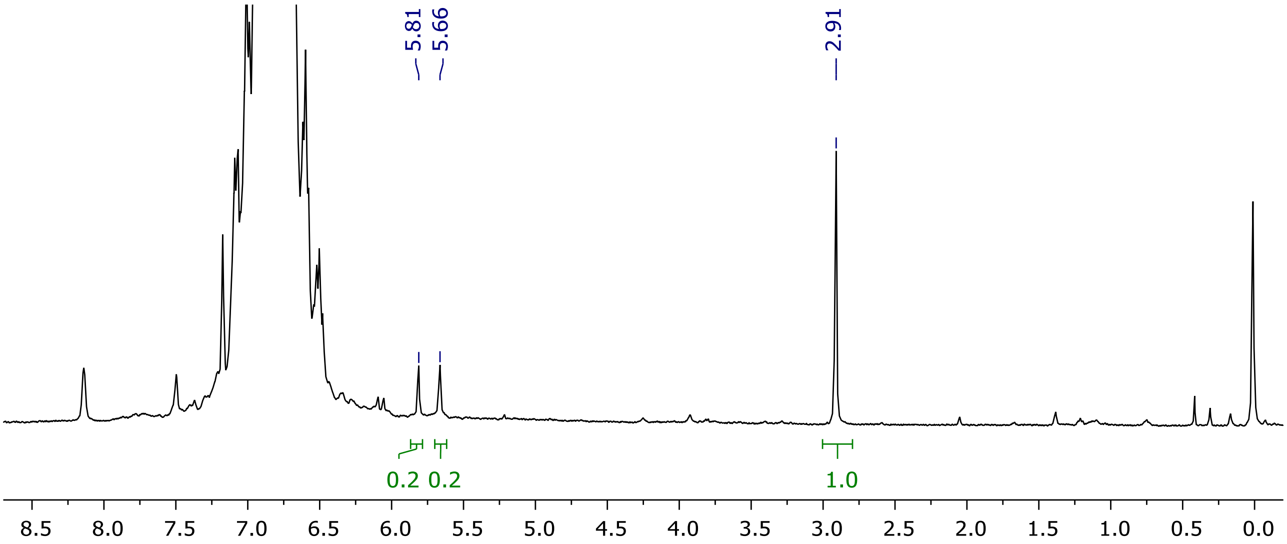

Figure S16: ^{1}H NMR spectrum collected during the synthesis of **4d** at RT (*t* = 24 h, DFB, 400 MHz).

2.5.2. Preparation of 4d

Bicyclo[4.2.0]octa-1,5,7-triene **4d** was isolated following the general procedure, quenching after 8 h. The resulting solution was reduced to dryness *in vacuo* and the residue washed with cold diethyl ether (0 °C) to afford the pure product as a pale yellow powder. Yield: 69 mg (75%). Crystals suitable for X-ray diffraction were grown from slow evaporation of a solution of the product in CH_2Cl_2 at RT.

^{1}H NMR (400 MHz, DFB, selected data): δ 2.67 (s, 4H, CH_2).

^{1}H NMR (500 MHz, CD_2Cl_2): δ 6.81 (tt, $^3J_{FH}$ = 9.0, $^4J_{HH}$ = 2.3, 2H, *p*-CH), 6.75 (tt, $^3J_{FH}$ = 8.9, $^4J_{HH}$ = 2.3, 2H, *p*-CH), 6.68 – 6.75 (m, 4H, *o*-CH), 6.59 – 6.66 (m, 4H, *o*-CH), 2.93 (s, 4H, CH_2).

^{13}C{^{1}H} NMR (126 MHz, CD_2Cl_2): δ 163.3 (dd, $^1J_{FC}$ = 248, $^3J_{FC}$ = 24, CF), 163.2 (dd, $^1J_{FC}$ = 248, $^3J_{FC}$ = 24, CF), 147.7 (t, $^4J_{FC}$ = 3, $C^{7/8}$), 142.2 (t, $^3J_{FC}$ = 10, *i*-C), 135.6 (s, $C^{1/6}$), 135.1 (t, $^3J_{FC}$ = 10, *i*-C), 119.1 (t, $^4J_{FC}$ = 3, $C^{2/5}$), 112.0 (m, *o*-Ar), 111.5 (m, *o*-Ar), 104.9 (t, $^2J_{FC}$ = 26, *p*-CH), 103.2 (t, $^2J_{FC}$ = 26, *p*-CH), 28.9 (s, $C^{3/4}H_2$).

^{19}F{^{1}H} NMR (377 MHz, CD_2Cl_2): δ -109.7 (s, 4F), -111.1 (s, 4F).

Anal. Calcd for $C_{32}H_{16}F_8$ (552.47 g·mol^{-1}): C, 69.57; H, 2.92; N, 0.00. Found: C, 69.72; H, 2.85; N, 0.00.

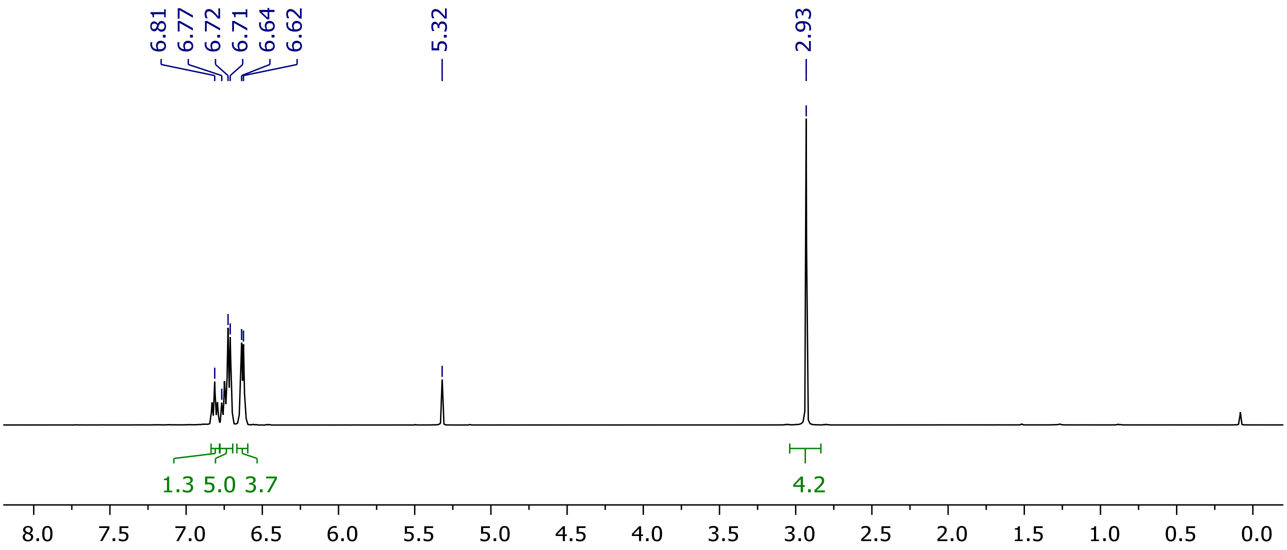

Figure S17: ^{1}H NMR spectrum of **4d** (CD_2Cl_2, 500 MHz).

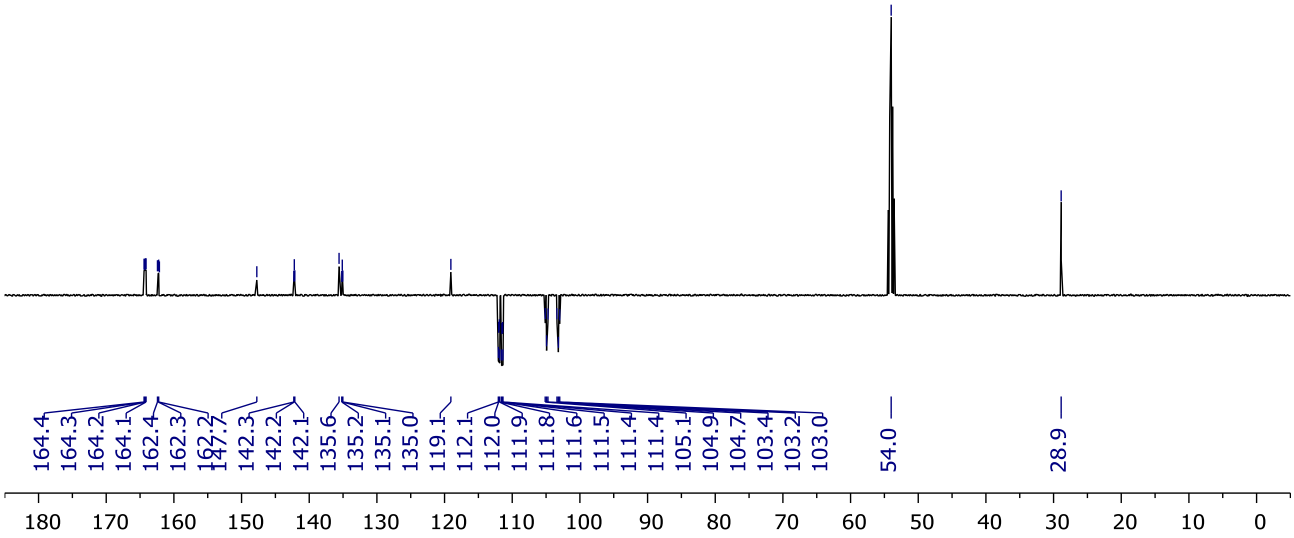

Figure S18: ^{13}C{^{1}H} APT NMR spectrum of **4d** (CD_2Cl_2, 126 MHz).

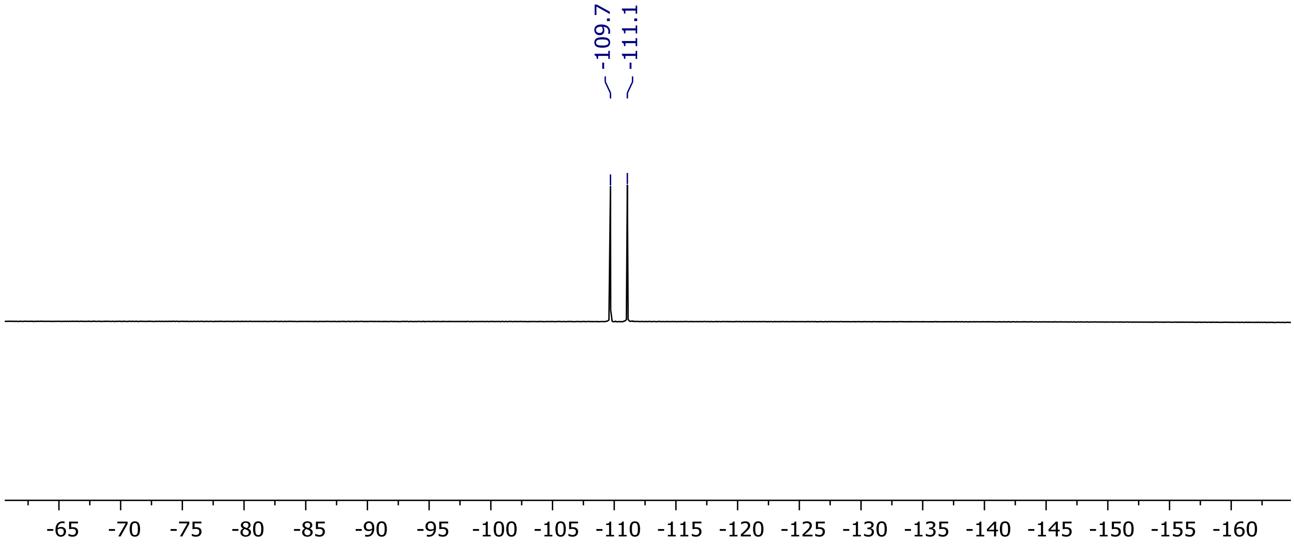

Figure S19: ^{19}F{^{1}H} NMR spectrum of **4d** (CD_2Cl_2, 377 MHz).

2.6. Synthesis of 4e

2.6.1. *In situ* experiment

The intermediate formation of **3e** during the **1·C₂H₄** catalysed formation of **4e** from **2e** was established using a reaction carried out in a J. Young's valve NMR tube (50 mM **2e**, 5 mol% **1**·C_2H_4, 0.5 mL DFB) that was periodically monitored *in situ* using ^{1}H NMR spectroscopy at RT. Formation of **3e** was apparent by CH_2 signals at δ 5.99 and 5.87.

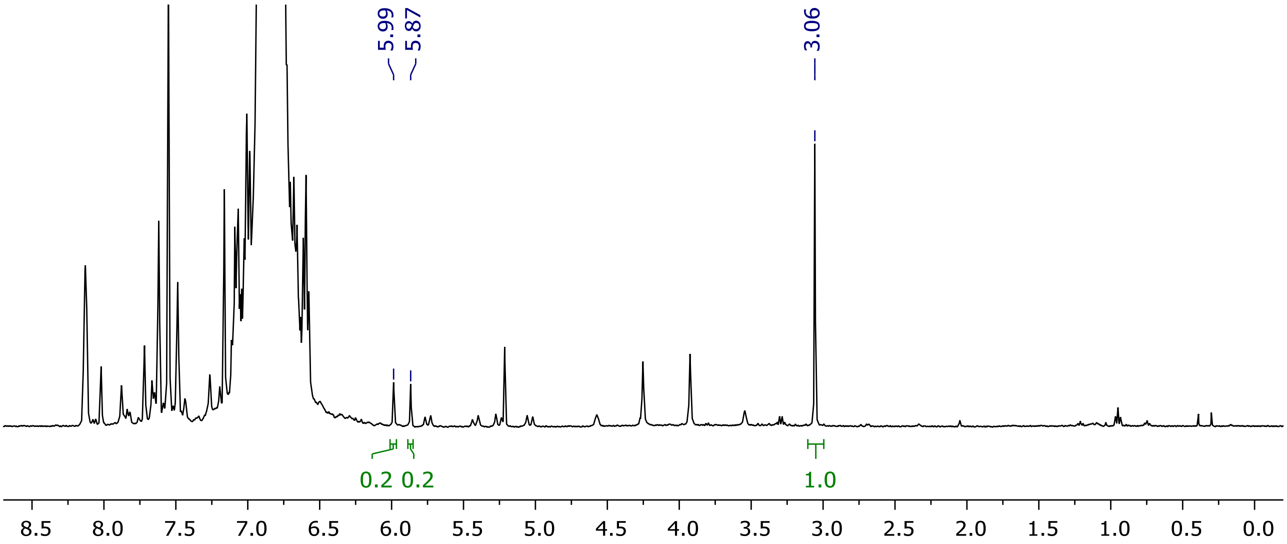

Figure S20: ^{1}H NMR spectrum collected during the synthesis of **4e** at RT (*t* = 2.5 h, DFB, 400 MHz).

2.6.2. Preparation of 4e

Bicyclo[4.2.0]octa-1,5,7-triene **4e** was isolated following the general procedure, quenching after 5.5 h. The resulting orange gel was concentrated to dryness and the crude product extracted into hexane. The pure compound was isolated as a pale-yellow oil following chromatography (SiO_2, hexane). Yield: 94.4 mg (79%).

^{1}H NMR (300 MHz, DFB, selected data): δ 2.92 (s, 4H, CH_2).

^{1}H NMR (600 MHz, $CDCl_3$): δ 7.83 (br, 2H, *p*-CH), 7.79 (br, 2H, *p*-CH), 7.59 (br, 4H, *o*-CH), 7.43 (br, 4H, *o*-CH), 3.11 (s, 4H, CH_2).

^{13}C{^{1}H} NMR (151 MHz, $CDCl_3$): δ 146.8 (s, $C^{7/8}$), 139.7 (s, *i*-C), 135.2 (s, $C^{1/6}$), 132.8 (s, *i*-C), 132.6 (q, $^2J_{FC}$ = 34, <u>C</u>CF_3), 132.0 (q, $^2J_{FC}$ = 34, <u>C</u>CF_3), 128.0 (s, *o*-CH), 127.9 (s, *o*-CH), 123.1 (s, *p*-CH), 123.1 (q, $^1J_{FC}$ = 273, CF_3), 122.7 (q, $^1J_{FC}$ = 273, CF_3), 121.8 (s, *p*-CH), 119.2 (s, $C^{2/5}$), 28.5 (s, $C^{3/4}H_2$).

^{19}F{^{1}H} NMR (377 MHz, $CDCl_3$): δ -63.43 (s, 12F), -63.74 (s, 12F).

Anal. Calcd for $C_{40}H_{16}F_{24}$ (952.53 g·mol^{-1}): C, 50.44; H, 1.69; N, 0.00. Found: C, 50.29; H, 1.69; N, 0.00.

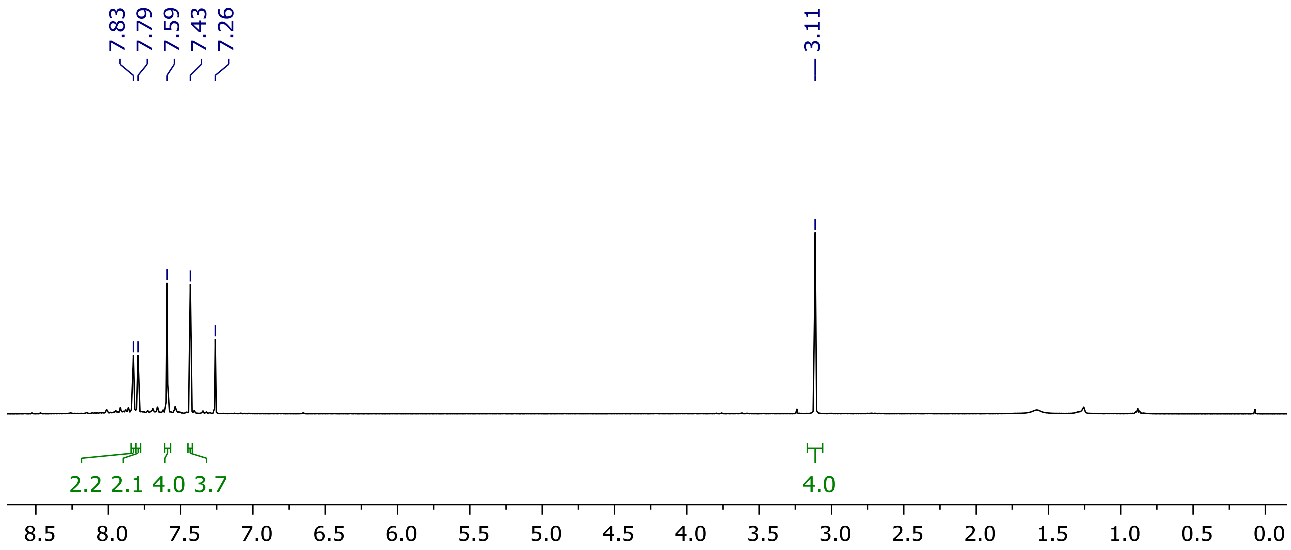

Figure S21: ^{1}H NMR spectrum of **4e** ($CDCl_3$, 600 MHz).

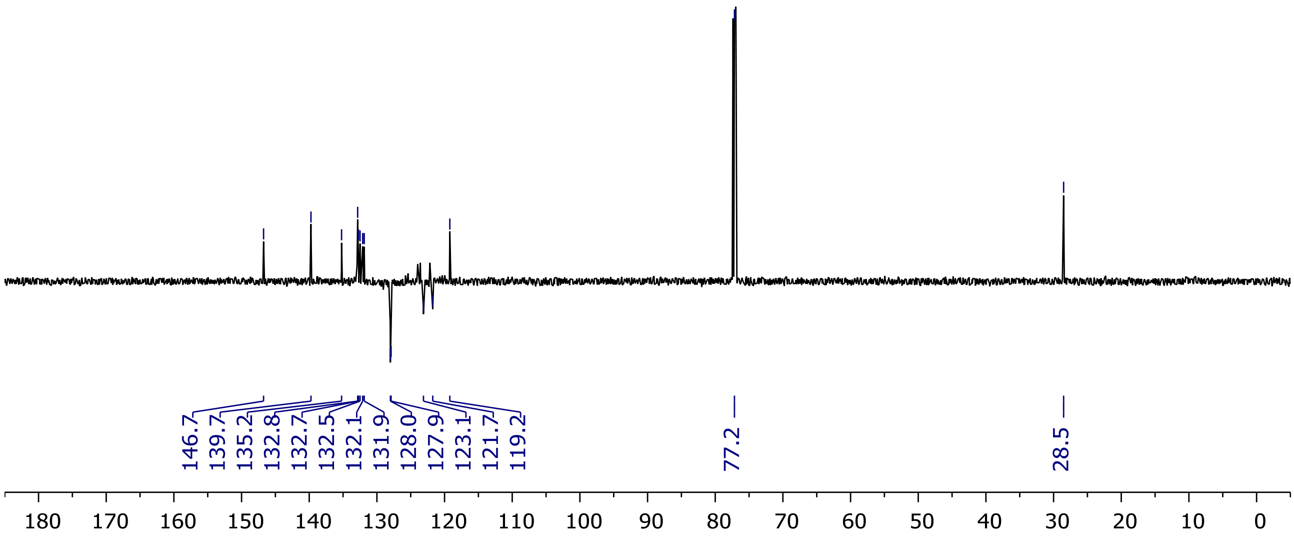

Figure S22: $^{13}C\{^{1}H\}$ APT NMR spectrum of **4e** ($CDCl_3$, 151 MHz).

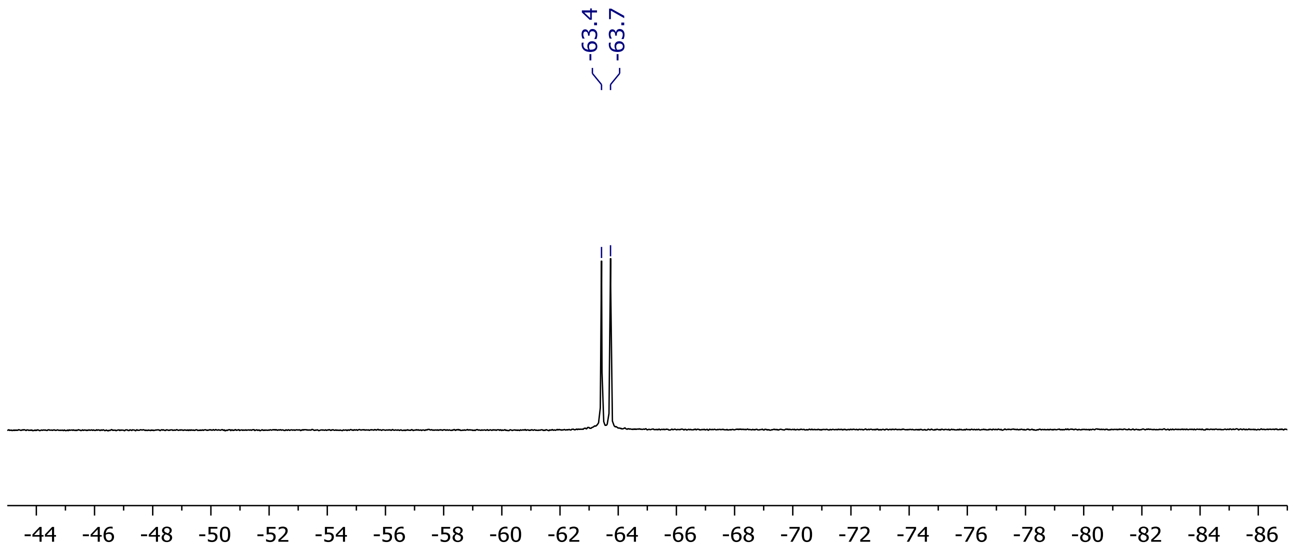

Figure S23: $^{19}F\{^{1}H\}$ NMR spectrum of **4e** ($CDCl_3$, 377 MHz).

2.7. Synthesis of 4f

2.7.1. *In situ* experiment

The intermediate formation of **3f** during the **1**·C_2H_4 catalysed formation of **4f** from **2f** was established using a reaction carried out in a J. Young's valve NMR tube (50 mM **2f**, 5 mol% **1**·C_2H_4, 0.5 mL DFB) that was periodically monitored *in situ* using ^{1}H NMR spectroscopy at RT. Formation of **3f** was apparent by CH_2 signals at δ 5.73 and 5.52.

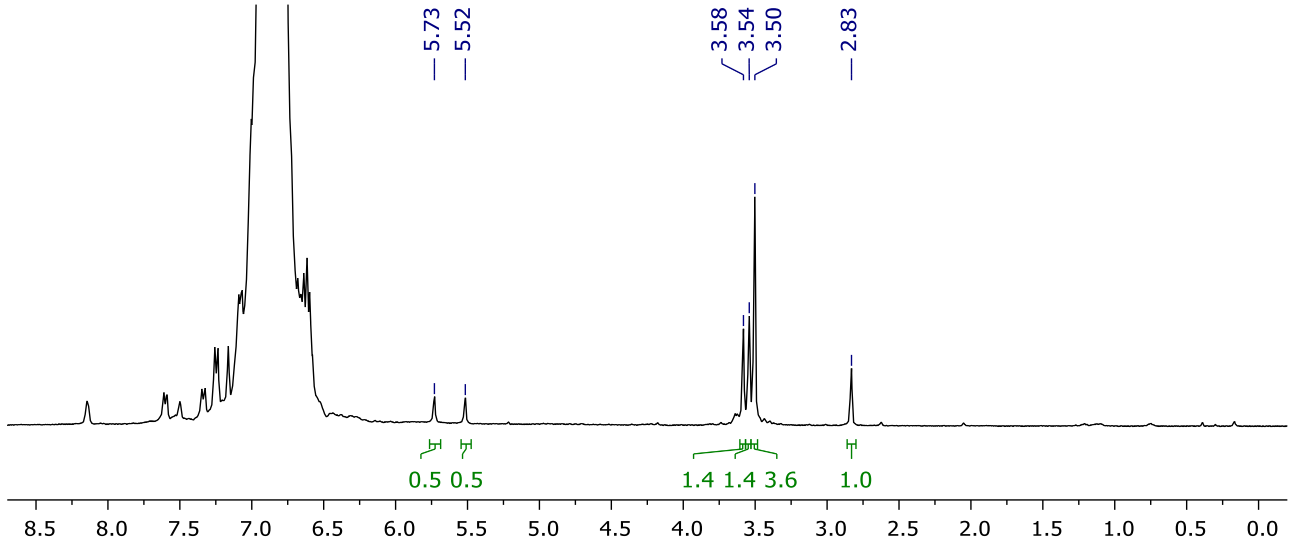

Figure S24: ^{1}H NMR spectrum collected during the synthesis of **4f** at RT (*t* = 24 h, DFB, 400 MHz).

2.7.2. Preparation of 4f

Bicyclo[4.2.0]octa-1,5,7-triene **4f** was isolated following the general procedure, quenching after 6.5 h. The resulting solution was reduced to dryness *in vacuo* and the residue washed with cold diethyl ether (0 °C) to afford the pure product as a pale-yellow solid. Yield: 52 mg (79%)

^{1}H NMR (300 MHz, DFB, selected data): δ 3.56 (s, 6H, CH_3), 3.51 (s, 6H, CH_3), 2.80 (s, 4H, CH_2).

^{1}H NMR (500 MHz, CD_2Cl_2): δ 7.15 (d, $^3J_{HH}$ = 8.7, 4H, *o*-CH), 7.07 (d, $^3J_{HH}$ = 8.7, 4H, *o*-CH), 6.74 (d, $^3J_{HH}$ = 8.7, 4H, *m*-CH), 6.71 (d, $^3J_{HH}$ = 8.7, 4H, *m*-CH), 3.78 (s, 12H, 2×CH_3), 2.90 (s, 4H, CH_2).

^{13}C{^{1}H} NMR (126 MHz, CD_2Cl_2): δ 160.0 (s, *p*-C), 159.1 (s, *p*-C), 146.9 (s, $C^{7/8}$), 134.9 (s, $C^{1/6}$), 132.8 (s, *i*-C), 130.7 (s, *o*-CH) 129.9 (s, *o*-CH), 126.5 (s, *i*-C), 117.0 (s, $C^{2/5}$), 113.7 (s, *m*-CH), 113.6 (s, *m*-CH), 55.77 (s, CH_3), 55.76 (s, CH_3), 29.7 (s, $C^{3/4}H_2$).

HR ESI-MS (positive ion, 4 kV): 551.2188 ([*M*+Na]$^+$, calcd 551.2193) *m/z*.

Anal. Calcd for $C_{36}H_{32}O_4$ (528.65 g·mol^{-1}): C, 81.79; H, 6.10; N, 0.00. Found: C, 81.67; H, 5.94; N, 0.00.

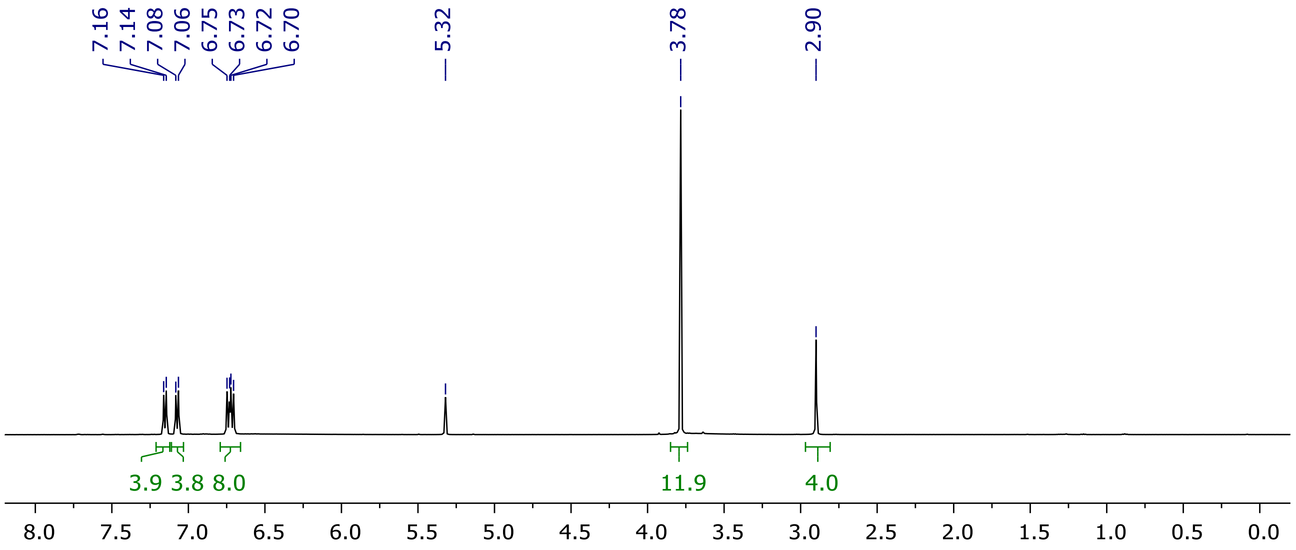

Figure S25: ^{1}H NMR spectrum of **4f** (CD_2Cl_2, 500 MHz).

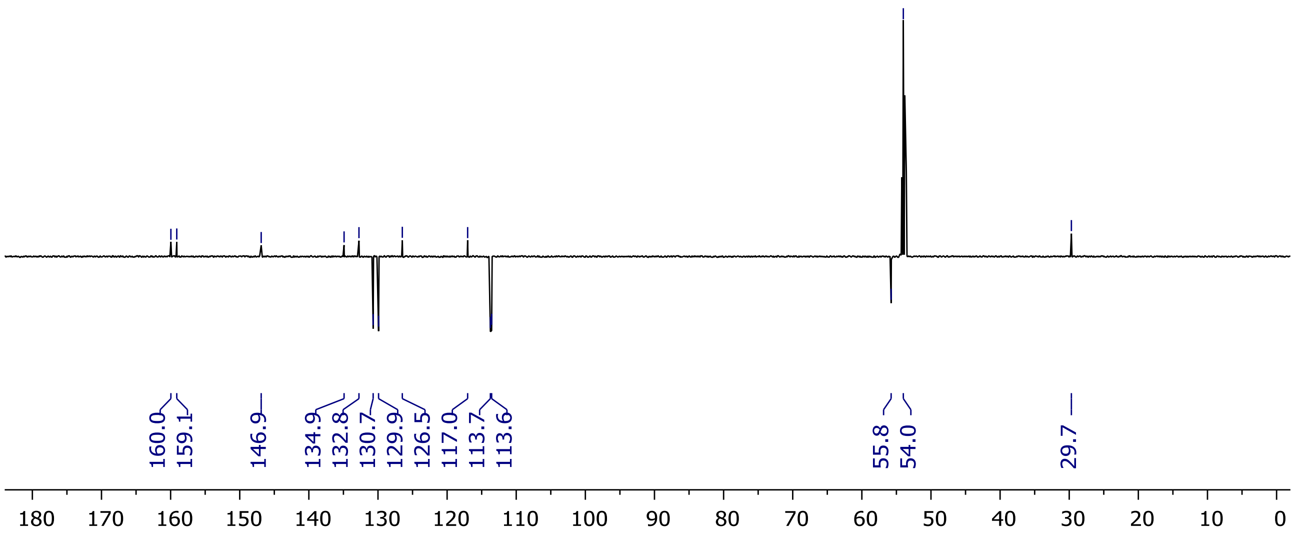

Figure S26: $^{13}C\{^{1}H\}$ APT NMR spectrum of **4f** (CD_2Cl_2, 126 MHz).

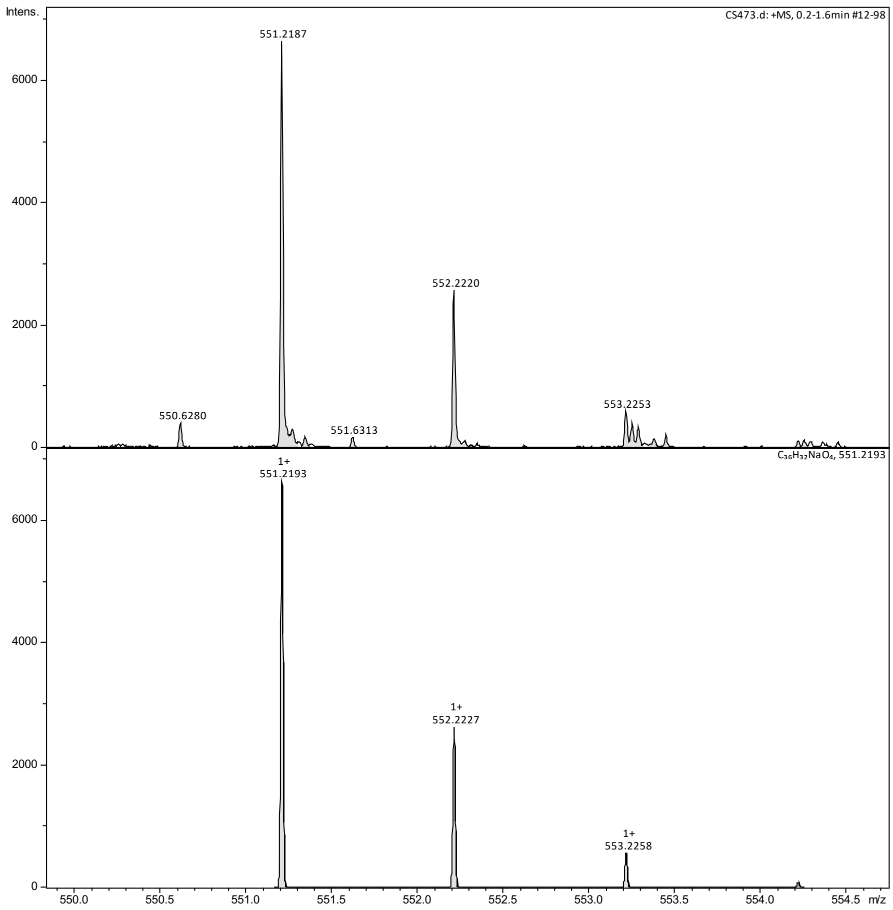

Figure S27: ESI-MS of **4f**.

2.8. Synthesis of 4g

2.8.1. *In situ* experiment

The intermediate formation of **3g** during the **1**·C_2H_4 catalysed formation of **4g** from **2g** was established using a reaction carried out in a J. Young's valve NMR tube (50 mM **2g**, 5 mol% **1**·C_2H_4, 0.5 mL DFB) that was periodically monitored *in situ* using ^{1}H NMR spectroscopy at RT. Formation of **3g** was apparent by CH_2 signals at δ 5.73 and 5.56.

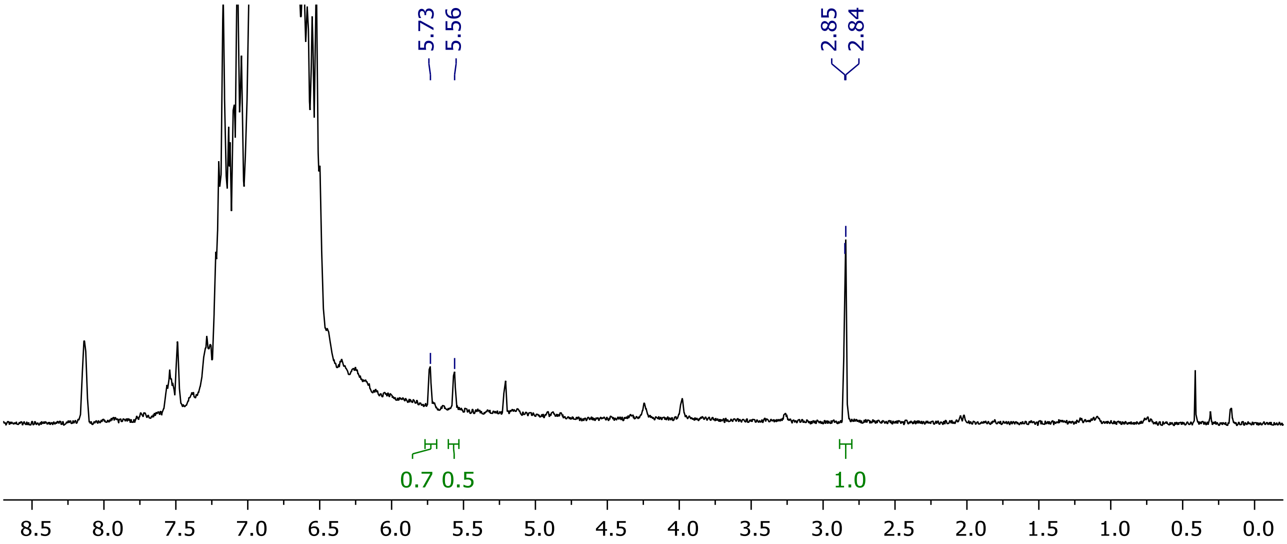

Figure S28: ^{1}H NMR spectrum collected during the synthesis of **4g** at RT (t < 1 h, DFB, 400 MHz).

2.8.2. Preparation of 4g

Bicyclo[4.2.0]octa-1,5,7-triene **4g** was isolated following the general procedure, quenching after 6 h. The resulting solution was reduced to dryness *in vacuo* and the product extracted into diethyl ether and passed through a short silica plug. Recrystallisation from a saturated diethyl ether solution at 4 °C afforded the product as pale-yellow needles. Yield: 49.1 mg (82%).

^{1}H NMR (300 MHz, DFB, selected data): δ 2.71 (s, 4H, CH_2).

^{1}H NMR (500 MHz, CD_2Cl_2): δ 7.18 (app. dd, J = 9, 6, 4H, *o*-CH), 7.07 (app. dd, J = 8, 6, 4H, *o*-CH), 6.87 – 6.94 (m, 8H, 2× *m*-CH), 2.93 (s, 4H, CH_2).

$^{13}C\{^1H\}$ NMR (126 MHz, CD_2Cl_2): δ 163.1 (d, $^1J_{FC}$ = 248, *p*-C), 162.5 (d, $^1J_{FC}$ = 246, *p*-C), 147.2 (s, $C^{7/8}$), 135.9 (d, $^4J_{FC}$ = 3, *i*-C), 135.2 (s, $C^{1/6}$), 131.1 (d, $^3J_{FC}$ = 8, *o*-CH), 130.3 (d, $^3J_{FC}$ = 8, *o*-CH), 129.5 (d, $^4J_{FC}$ = 4, *i*-C), 117.7 (s, $C^{2/5}$), 115.6 (d, $^2J_{FC}$ = 22, *m*-CH), 115.1 (d, $^2J_{FC}$ = 21, *m*-CH), 29.5 (s, $C^{3/4}H_2$).

$^{19}F\{^1H\}$ NMR (376 MHz, CD_2Cl_2): δ -112.93 (s, 2F), -115.85 (s, 2F)

Anal. Calcd for $C_{32}H_{20}F_4$ (480.51 g·mol^{-1}): C, 79.99; H, 4.20; N, 0.00. Found: C, 79.90; H, 4.15; N, 0.00.

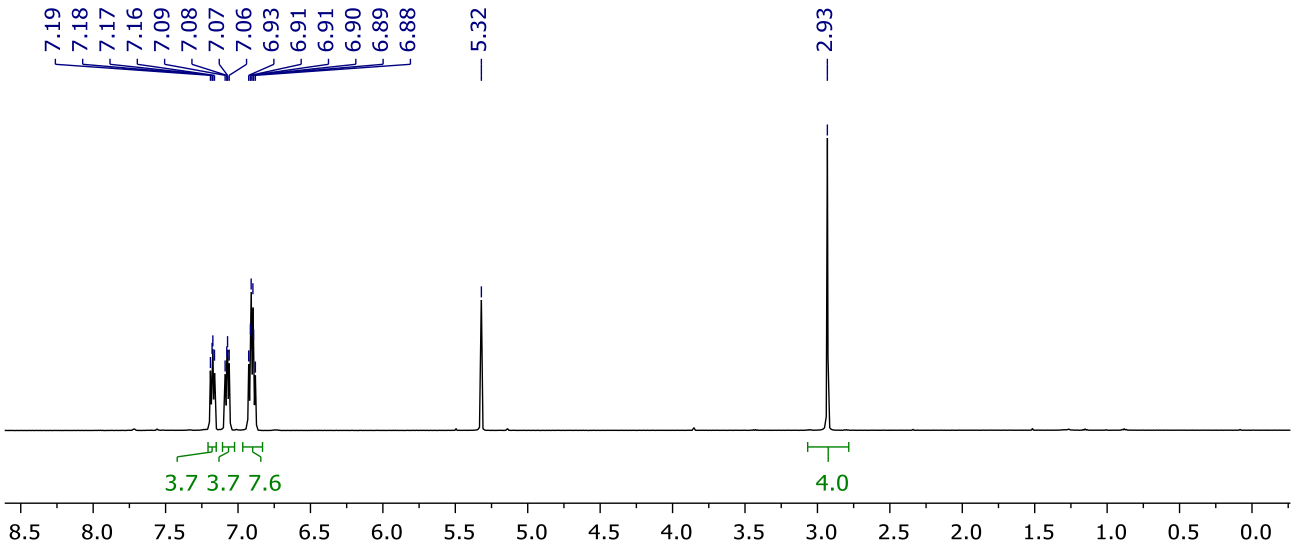

Figure S29: ^{1}H NMR spectrum of **4g** (CD_2Cl_2, 500 MHz).

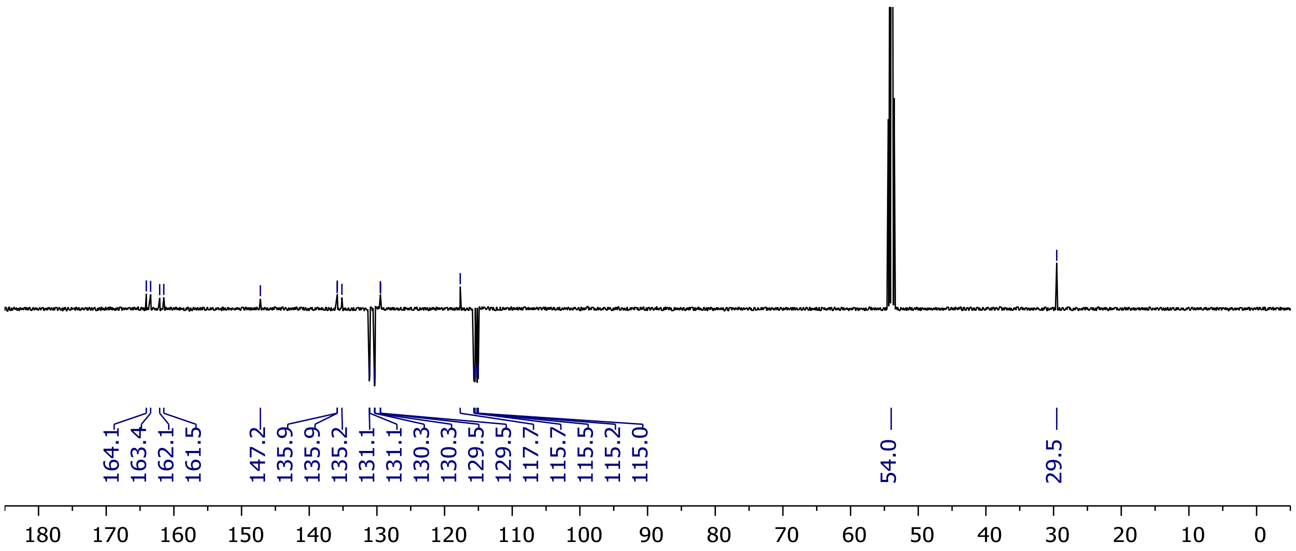

Figure S30: $^{13}C\{^1H\}$ APT NMR spectrum of **4g** (CD_2Cl_2, 126 MHz).

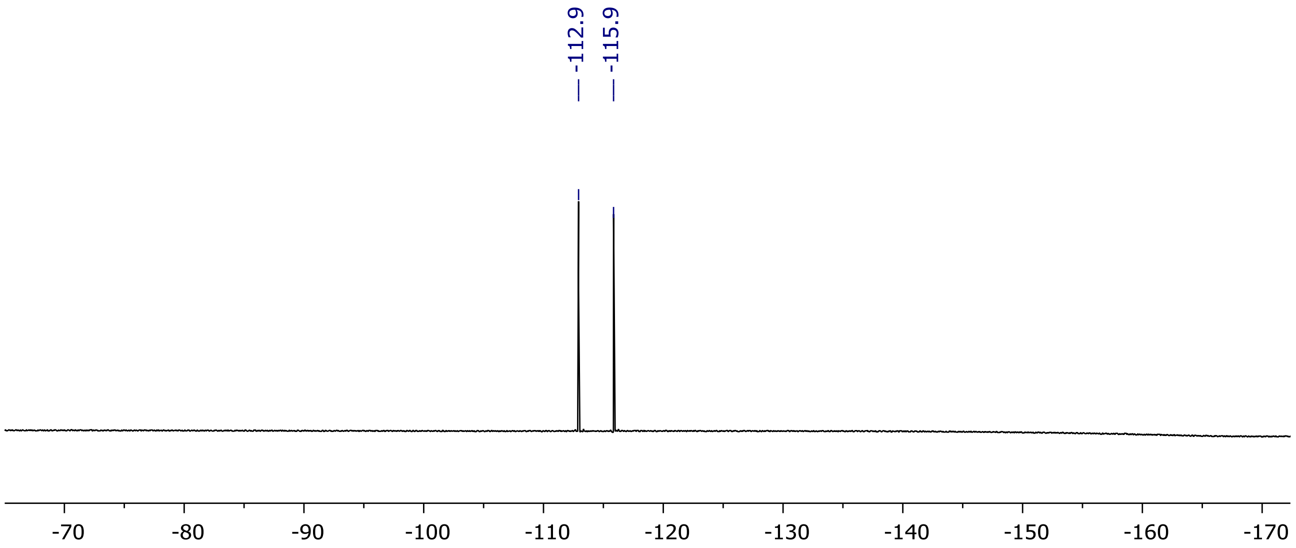

Figure S31: $^{19}F\{^1H\}$ NMR spectrum of **4g** (CD_2Cl_2, 377 MHz).

2.9. Attempted synthesis of 4h

2.9.1. *In situ* experiment

The synthesis of bicyclo[4.2.0]octa-1,5,7-triene **4h** was attempted using a reaction carried out in a J. Young's valve NMR tube (100 mM **2h**, 5 mol% **1**·C_2H_4, 0.5 mL DFB) that was monitored *in situ* using ^{1}H NMR spectroscopy at 25 °C. Complete consumption of **2h** and formation of a 60:40 mixture of *E*-MesC≡CCHCHMes/**3h** in 99% spectroscopic yield was observed after 14 h.

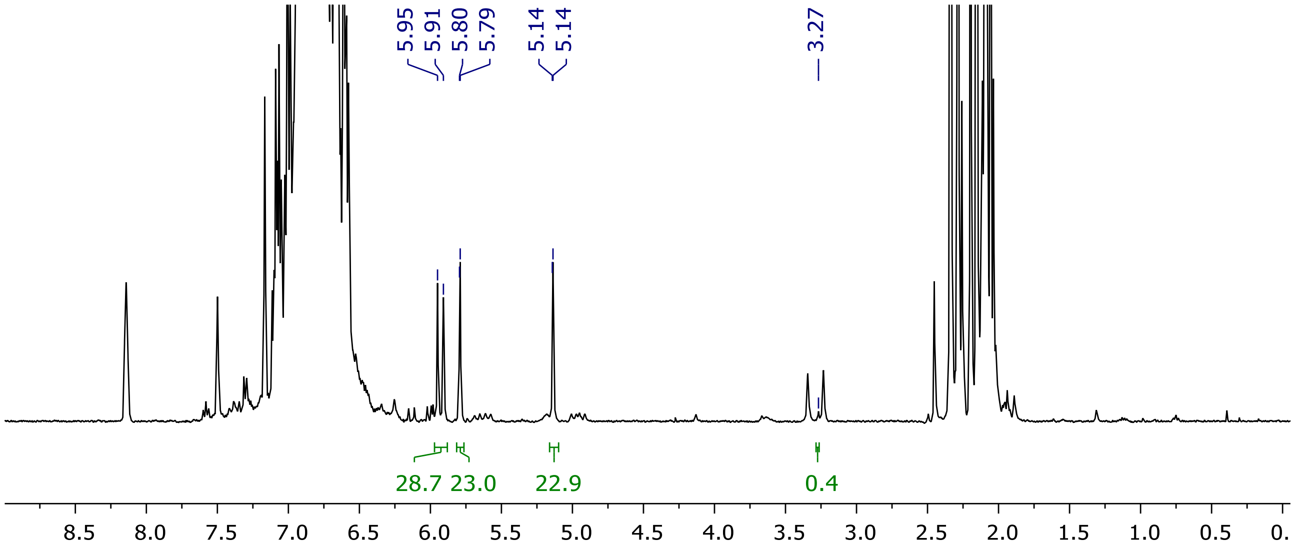

Figure S32: ^{1}H NMR spectrum collected during the attempted synthesis of **4h** at 25 °C (*t* = 14 h, DFB, 400 MHz).

2.9.2. General procedure

Synthesis of bicyclo[4.2.0]octa-1,5,7-triene **4h** was attempted using the general procedure. Complete consumption of **2h** and formation of a 20:80 mixture of *E*-MesC≡CCHCHMes/**3h** in 100% spectroscopic yield was observed after 8 h by ^{1}H NMR spectroscopy. No changes were observed on additional heating (total 24 h).

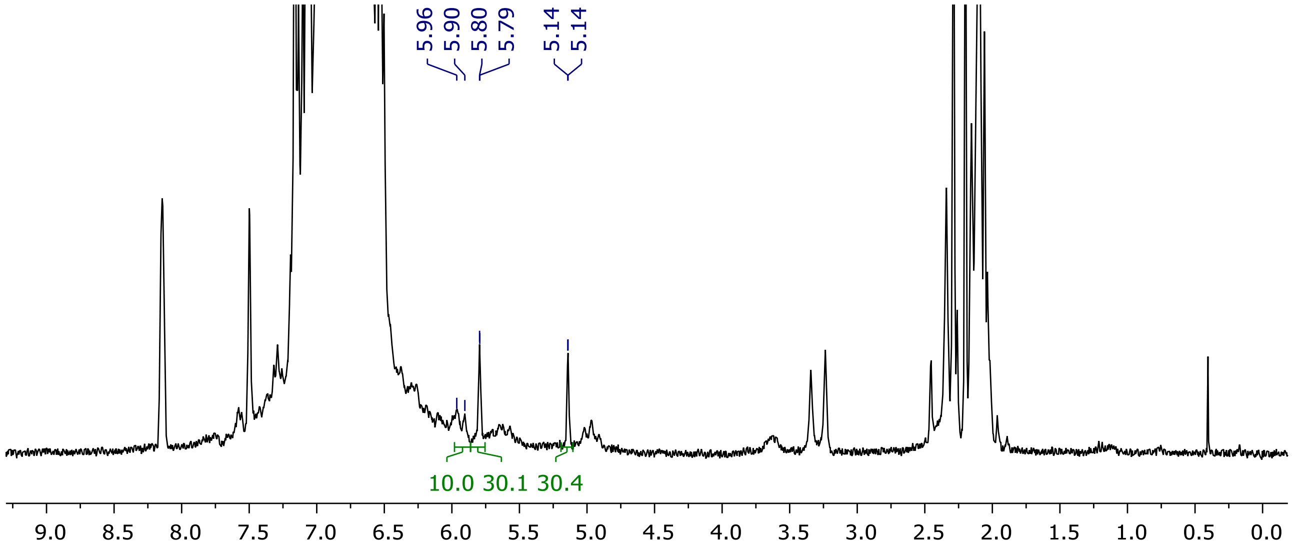

Figure S33: ^{1}H NMR spectrum collected during the attempted synthesis of **4h** at 65 °C (*t* = 8 h, DFB, 300 MHz).

2.9.3. Preparation of *E*-MesC≡CCHCHMes and 3h

A solution of **2h** (34 µL, 0.22 mmol) in DFB (2.1 mL) was added to a flask charged with **1**·C_2H_4 (13.6 mg, 0.011 mmol) and the resulting orange solution stirred at RT for 18 h. The solution was freeze-pump-thaw degassed placed under an atmosphere of CO (1 atm) and then concentrated to dryness. The resulting residue was extracted into hexane through a short silica plug to give a *ca.* 60:40 mixture of *E*-MesC≡CCHCHMes/**3h**. Yield: 28mg (90%). Separation was achieved using chromatography (SiO_2, 99:1 hexane/toluene), affording analytically pure *E*-MesC≡CCHCHMes as a white solid (15 mg, 48%) and analytically pure **3h** as a pale-yellow oil (9 mg, 29%).

Data for *E*-MesC≡CCHCHMes is consistent with the literature.[7]

^{1}H NMR (300 Hz, DFB, selected data): δ 5.93 (d, $^3J_{HH}$ = 16.8, 1H, CH=CH), 2.34 (s, 6H, *o*-CH_3), 2.16 (s, 6H, *o*-CH_3), 2.09 (s, 6H, 2×*p*-CH_3).

^{1}H NMR (300 MHz, $CDCl_3$): δ 7.05 (d, $^3J_{HH}$ = 16.4, 1H, CH=CH), 6.89 (s, 2H, *m*-CH), 6.89 (s, 2H, *m*-CH), 6.01 (d, $^3J_{HH}$ = 16.4, 1H, CH=CH), 2.44 (s, 6H, *o*-CH_3), 2.35 (s, 6H, *o*-CH_3), 2.28 (s, 6H, 2×*p*-CH_3).

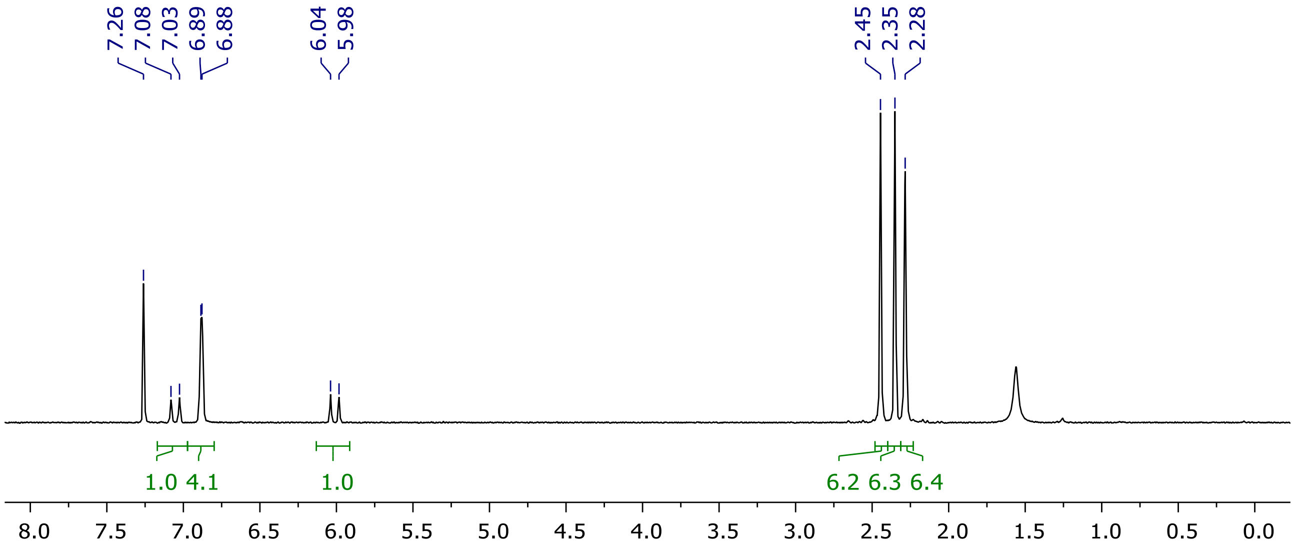

Figure S34: ^{1}H NMR spectrum of *E*-MesC≡CCHCHMes ($CDCl_3$, 300 MHz).

Data for **3h**:

^{1}H NMR (400 Hz, DFB, selected data): δ 5.79 (d, $^2J_{HH}$ = 2.1, 1H, CH_2), 5.14 (d, $^2J_{HH}$ = 2.0, 1H, CH_2), 2.28 (s, 6H, *o*-CH_3), 2.20 (s, 6H, *o*-CH_3), 2.09 (s, 3H, *p*-CH_3), 2.06 (s, 3H, *p*-CH_3).

^{1}H NMR (500 MHz, $CDCl_3$): δ 6.88 (s, 2H, *m*-CH), 6.82 (s, 2H, *m*-CH), 5.88 (d, $^2J_{HH}$ = 2.1, 1H, CH_2), 5.30 (d, $^2J_{HH}$ = 2.1, 1H, CH_2), 2.35 (s, 6H, *o*-CH_3), 2.32 (s, 6H, *o*-CH_3), 2.28 (s, 3H, *p*-CH_3), 2.25 (s, 3H, *p*-CH_3).

^{13}C{^{1}H} NMR (126 MHz, CD_2Cl_2): δ 140.2 (s, *o*-C), 137.8 (s, *p*-C), 136.9 (s, *i*-C), 136.8 (s, *p*-C), 135.5 (s, *o*-C), 130.5 (s, $\underline{C}(CH_2)$), 128.3 (s, *m*-CH), 127.6 (s, *m*-CH), 124.5 (s, CH_2), 120.2 (s, *i*-C), 97.2 (s, MesC≡$\underline{C}$), 87.2 (s, Mes$\underline{C}$≡C), 21.5 (s, *p*-CH_3), 21.2 (s, *p*-CH_3), 21.0 (s, *o*-CH_3), 20.2 (s, *o*-CH_3).

Anal. Calcd for $C_{22}H_{24}$ (288.43 g·mol^{-1}): C, 91.61; H, 8.39; N, 0.00. Found: C, 91.70; H, 8.21; N, 0.00.

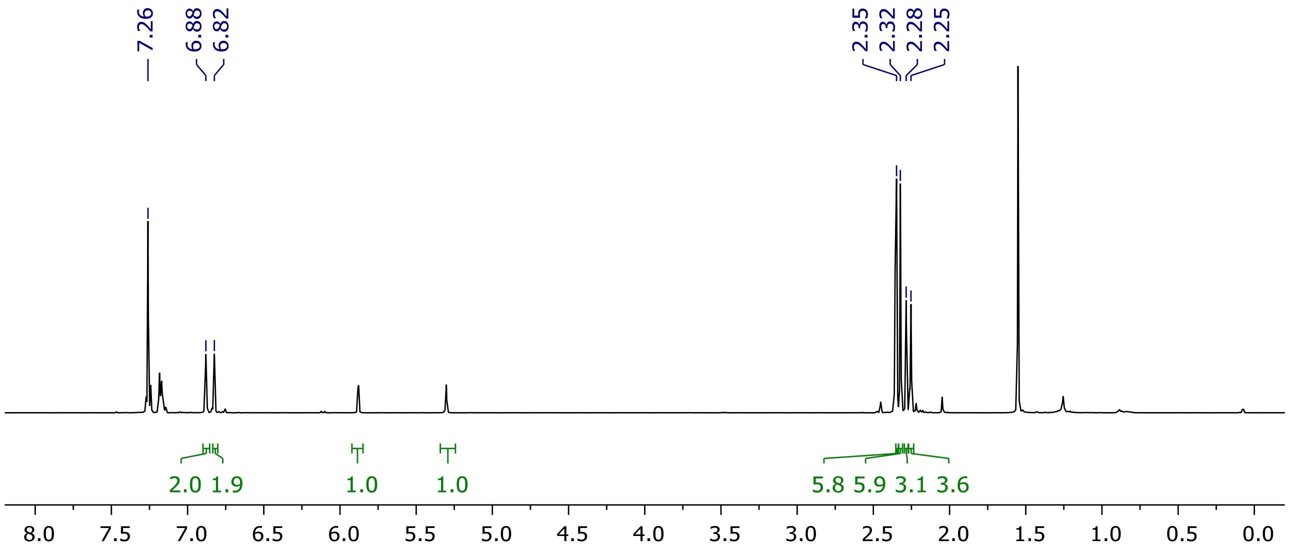

Figure S35: ^{1}H NMR spectrum of **3h** ($CDCl_3$, 500 MHz).

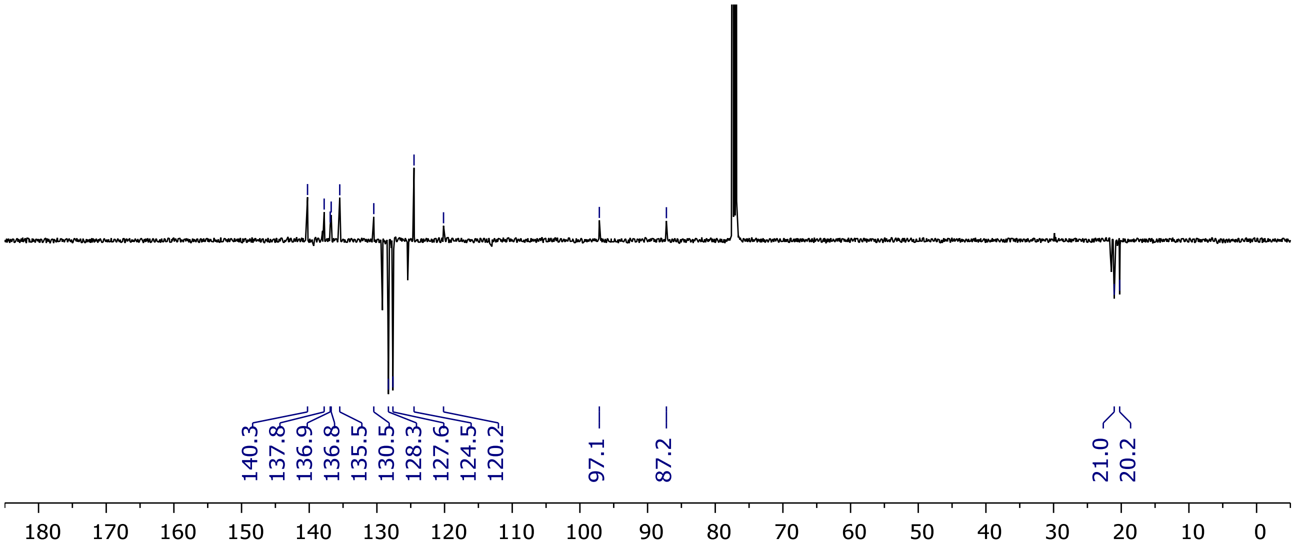

Figure S36: $^{13}C\{^{1}H\}$ APT NMR spectrum of **3h** ($CDCl_3$, 126 MHz).

2.10. Attempted synthesis of 4i

2.10.1. *In situ* experiment

The synthesis of bicyclo[4.2.0]octa-1,5,7-triene **4i** was attempted using a reaction carried out in a J. Young's valve NMR tube (100 mM **2i**, 5 mol% **1**·C_2H_4, 0.5 mL DFB) that was monitored *in situ* using ^{1}H NMR spectroscopy at 25 °C. Partial consumption of **2i** and formation of a 18:82 mixture of *E*-*n*BuC≡CCHCH*n*Bu ($\delta_{CH=CH}$ 5.90, 5.35; $^3J_{HH}$ = 16 Hz) / **3h** ($\delta_{C=CH2}$ 5.14, 5.02) in 68% spectroscopic yield was observed after 7 h. Data for the enynes are consistent with the literature.[8,9]

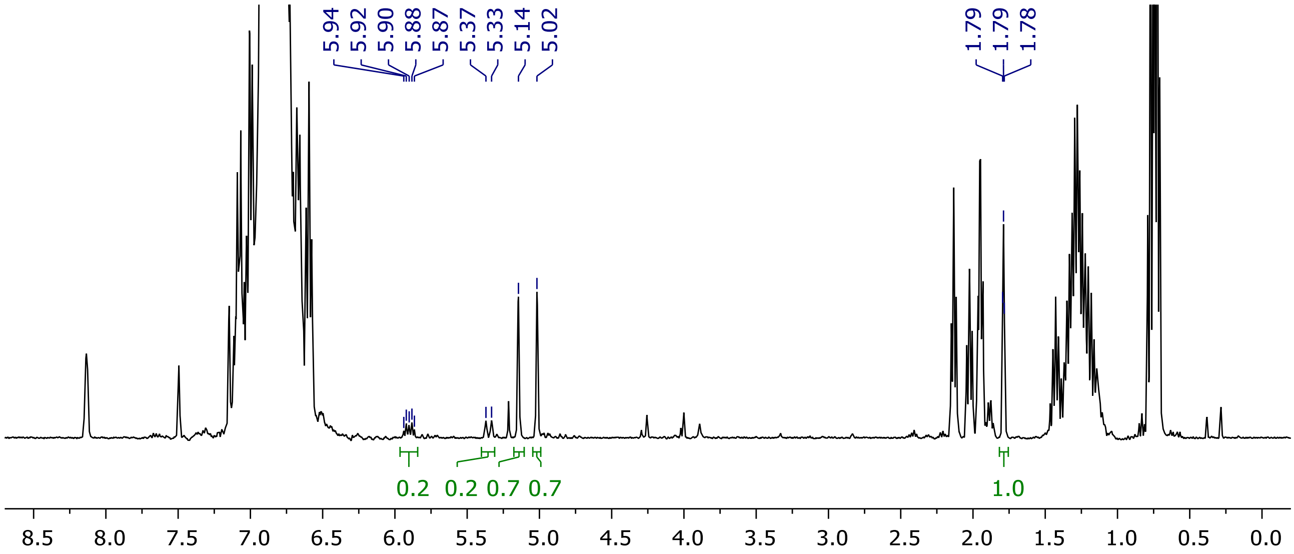

Figure S37: ^{1}H NMR spectrum collected during the attempted synthesis of **4i** at 25 °C (*t* = 7 h, DFB, 400 MHz).

2.10.2. General procedure

Synthesis of bicyclo[4.2.0]octa-1,5,7-triene **4i** was attempted using the general procedure. Complete consumption of **2i** and formation of **3i** in 90% spectroscopic yield was observed after 8 h by ^{1}H NMR spectroscopy. No changes were observed on additional heating (total 24 h).

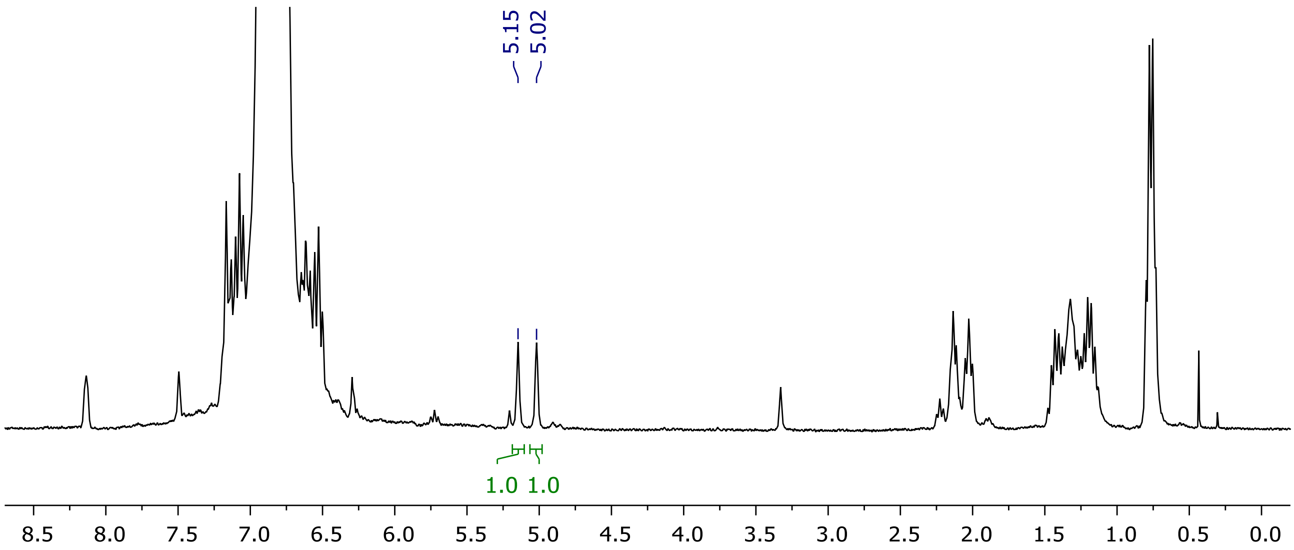

Figure S38: ^{1}H NMR spectrum collected during the attempted synthesis of **4i** at 65 °C (*t* = 8 h, DFB, 300 MHz).

2.11. Attempted synthesis of 4j

2.11.1. *In situ* experiment

The synthesis of bicyclo[4.2.0]octa-1,5,7-triene **4j** was attempted using a reaction carried out in a J. Young's valve NMR tube (100 mM **2j**, 5 mol% **1**·C_2H_4, 0.5 mL DFB) that was monitored *in situ* using ^1H NMR spectroscopy at 25 °C. Partial consumption of **2j** and exclusive formation of *E*-*t*BuC≡CCHCH*t*Bu (δ_{CH} 5.95, 5.29; $^3J_{HH}$ = 16 Hz) in 66% spectroscopic yield was observed after 14.5 h. Data for the enyne is consistent with the literature.[10]

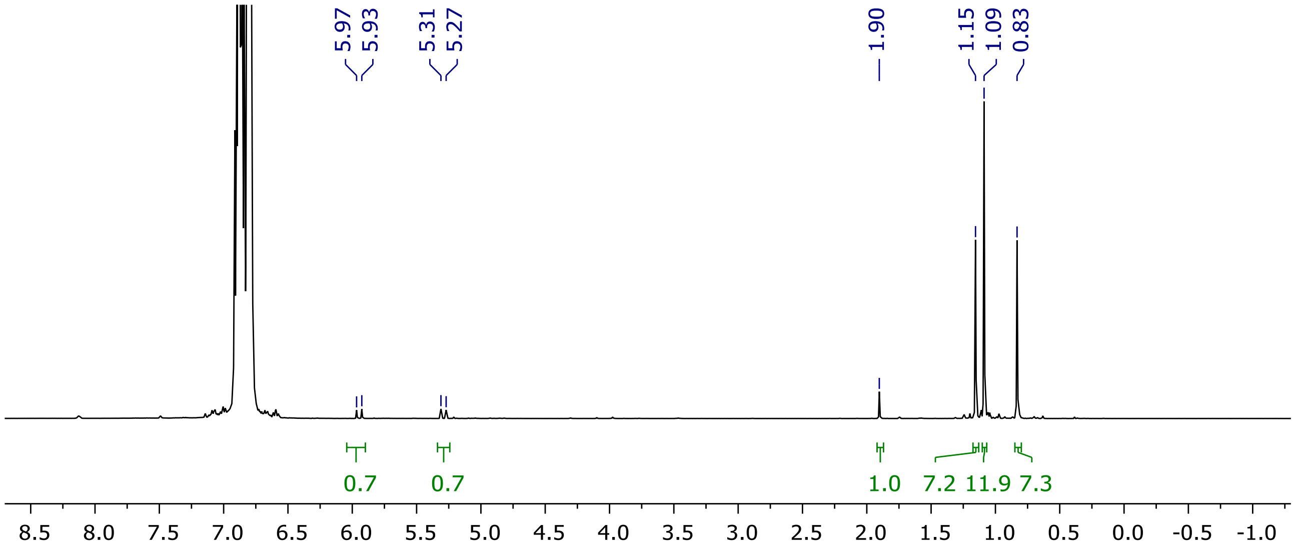

Figure S39: ^1H NMR spectrum collected during the attempted synthesis of **4j** at 25 °C (*t* = 7 h, DFB, 400 MHz).

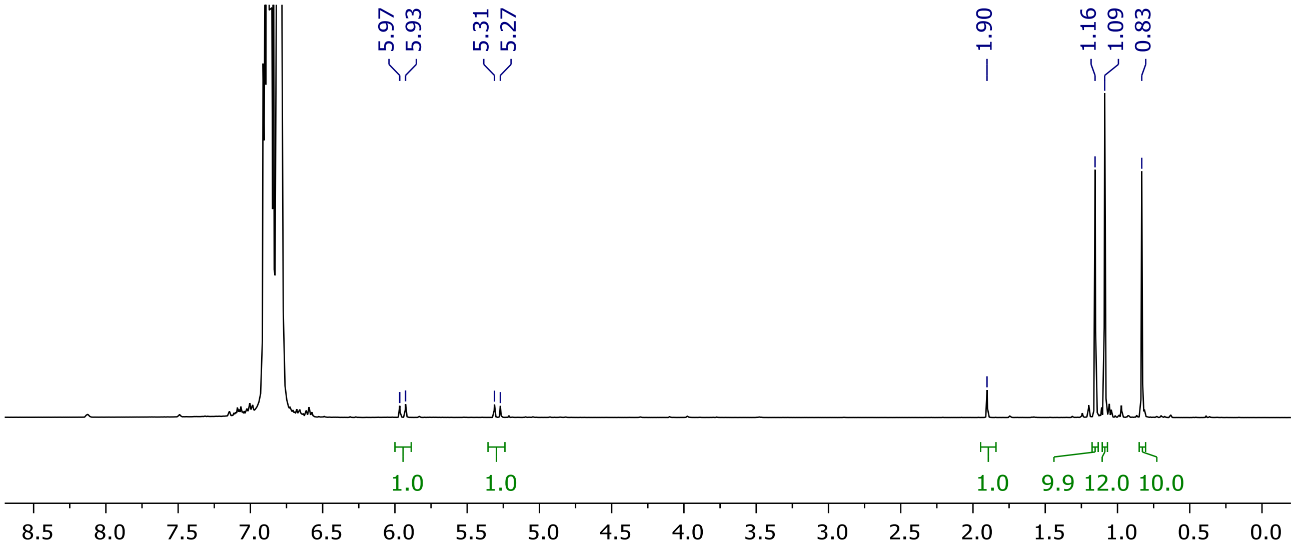

Figure S40: ^1H NMR spectrum collected during the attempted synthesis of **4j** at 25 °C (*t* = 14 h, DFB, 400 MHz).

2.11.2. General procedure

Synthesis of bicyclo[4.2.0]octa-1,5,7-triene **4j** was attempted using the general procedure. Complete consumption of **2j** and formation of *E*-*t*BuC≡CCHCH*t*Bu in 70% spectroscopic yield (free and complexed), alongside a balance trimer, was observed after 8 h by ^{1}H NMR spectroscopy. No changes were observed on additional heating (total 24 h).

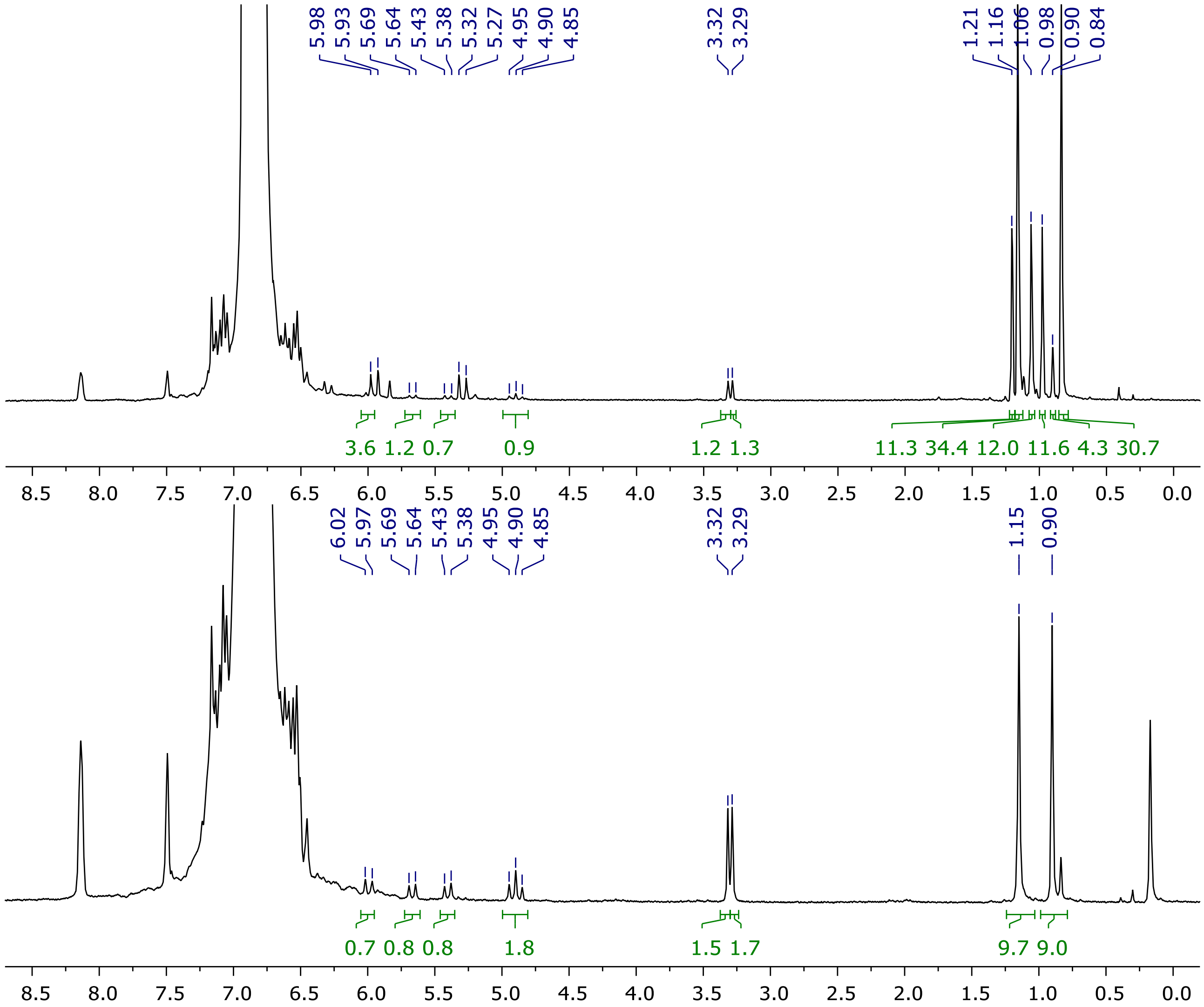

Figure S41: ^{1}H NMR spectrum collected during the attempted synthesis of **4j** at 65 °C (t = 8 h) and comparison to that of independently prepared **5** (DFB, 300 MHz).

2.11.3. Synthesis of [Rh(CNC-Me)(*E*-*t*BuC≡CCHCH*t*Bu)][BAr^F_4] 5

A solution of **1**·C_2H_4 (28.8 mg, 0.0228 mmol) in DFB (1 mL) was added to a flask charged with *E*-*t*BuC≡CCHCH*t*Bu (4.1 mg, 0.025 mmol) and the resulting red solution stirred for 2 h. Volatiles were removed *in vacuo* and the residue washed with pentane to afford the product as a bright red solid. Yield: 30.1 mg (94%). Crystals suitable for X-ray diffraction were grown from CH_2Cl_2/hexane at -30 °C.

^{1}H NMR (300 MHz, DFB, selected data): δ 8.09 – 8.18 (m, 8H, Ar^F), 7.49 (br, 4H, Ar^F), 5.99 (d, 1H, $^3J_{HH}$ = 15.1, C<u>H</u>CH*t*Bu), 5.67 (d, $^2J_{HH}$ = 14.5, 1H, pyC<u>H</u>$_2$), 5.40 (d, $^2J_{HH}$ = 14.7, 1H, pyC<u>H</u>$_2$), 4.90 (app. t, *J* = 14.6, 2H, pyC<u>H</u>$_2$), 3.32 (s, 3H, NCH_3), 3.29 (s, 3H, NCH_3), 1.15 (s, 9H, <u>*t*Bu</u>C≡C), 0.90 (s, 9H, CH<u>*t*Bu</u>).

^{1}H NMR (500 MHz, CD_2Cl_2): δ 7.76 (t, $^3J_{HH}$ = 7.7, 1H, py), 7.70 – 7.74 (m, 8H, Ar^F), 7.55 (br, 4H, Ar^F), 7.46 (d, $^3J_{HH}$ = 7.7, 1H, py), 7.43 (d, $^3J_{HH}$ = 7.7, 1H, py), 7.07 (d, $^3J_{HH}$ = 1.9, 1H, NCH), 7.02 (d, $^3J_{HH}$ = 1.9, 1H, NCH), 6.71 (d, $^3J_{HH}$ = 1.9, 1H, NCH), 6.67 (d, $^3J_{HH}$ = 1.8, 1H, NCH), 6.38 (d, $^3J_{HH}$ = 15.3, 1H, CHC<u>H</u>*t*Bu), 5.97 (d, $^3J_{HH}$ = 15.3, 1H, C<u>H</u>CH*t*Bu), 5.84 (d, $^2J_{HH}$ = 14.6, 1H, pyC<u>H</u>$_2$), 5.60 (d, $^2J_{HH}$ = 14.4, 1H, pyC<u>H</u>$_2$), 5.11 (d, $^2J_{HH}$ = 14.6, 1H, pyC<u>H</u>$_2$), 5.06 (d, $^2J_{HH}$ = 14.4, 1H, pyC<u>H</u>$_2$), 3.44 (s, 3H, NCH_3), 3.41 (s, 3H, NCH_3), 1.23 (s, 9H, <u>*t*Bu</u>C≡C), 0.96 (s, 9H, CH<u>*t*Bu</u>).

^{13}C{^{1}H} NMR (126 MHz, CD_2Cl_2): δ 184.9 (d, $^1J_{RhC}$ = 41, NCN), 184.5 (d, $^1J_{RhC}$ = 41, NCN), 162.3 (q, $^1J_{CB}$ = 50, Ar^F), 155.7 (s, py), 155.5 (s, py), 153.5 (d, $^3J_{RhC}$ = 2, CH<u>C</u>H*t*Bu), 138.5 (s, py), 135.4 (br, Ar^F), 129.4 (qq, $^2J_{FC}$ = 32, $^3J_{CB}$ = 3, Ar^F), 125.1 (q, $^1J_{FC}$ = 271, Ar^F), 124.2 (s, 2×py), 122.4 (s, NCH), 122.3 (s, NCH), 120.8 (s, NCH), 120.3 (s, NCH), 118.0 (sept, $^3J_{FC}$ = 4, Ar^F), 110.8 (d, $^2J_{RhC}$ = 1, <u>C</u>HCH*t*Bu), 97.2 (d, $^1J_{RhC}$ = 15, *t*Bu<u>C</u>≡C), 75.1 (d, $^1J_{RhC}$ = 12, *t*BuC≡<u>C</u>), 56.3 (d, $^3J_{RhC}$ = 2, py<u>C</u>H$_2$), 56.0 (d, $^3J_{RhC}$ = 2, py<u>C</u>H$_2$), 37.0 (s, NCH_3), 36.5 (s, NCH_3), 33.9 (s, CH<u>*t*Bu</u>{C}), 33.1 (s, <u>*t*Bu</u>C≡C{C}), 31.2 (s, <u>*t*Bu</u>C≡C{CH_3}), 29.7 (s, CH<u>*t*Bu</u>{CH_3}).

Anal. Calcd for $C_{59}H_{49}BF_{24}N_5Rh$ (1397.75 g mol^{-1}): C, 50.70; H, 3.53; N, 5.01. Found: C, 50.56; H, 3.45; N, 4.99.

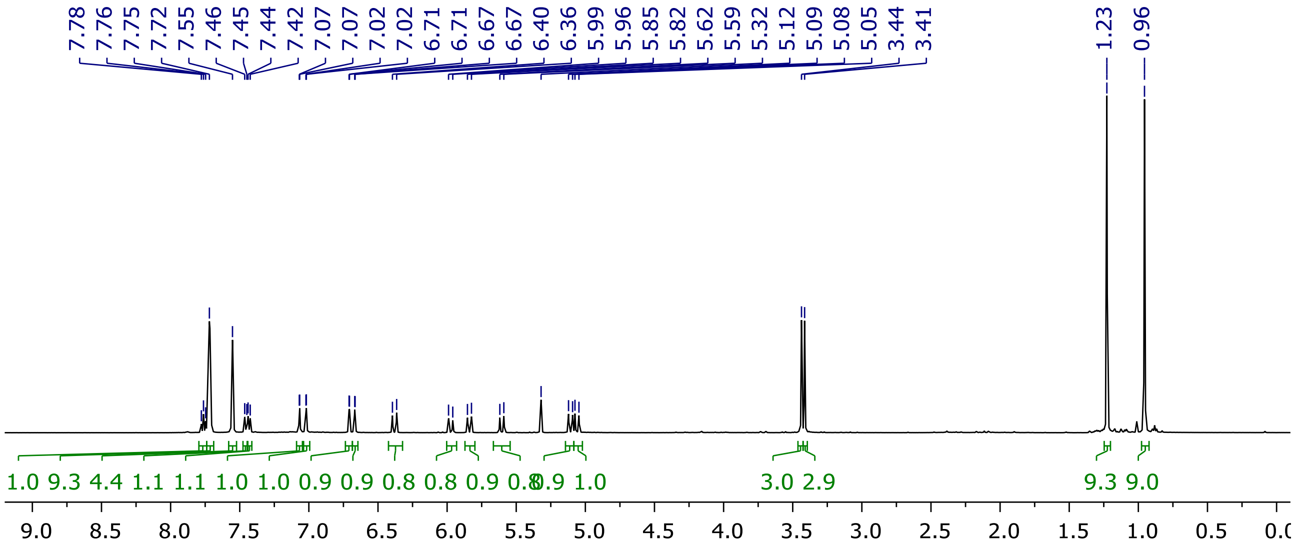

Figure S42: ^{1}H NMR spectrum of **5** (CD_2Cl_2, 500 MHz).

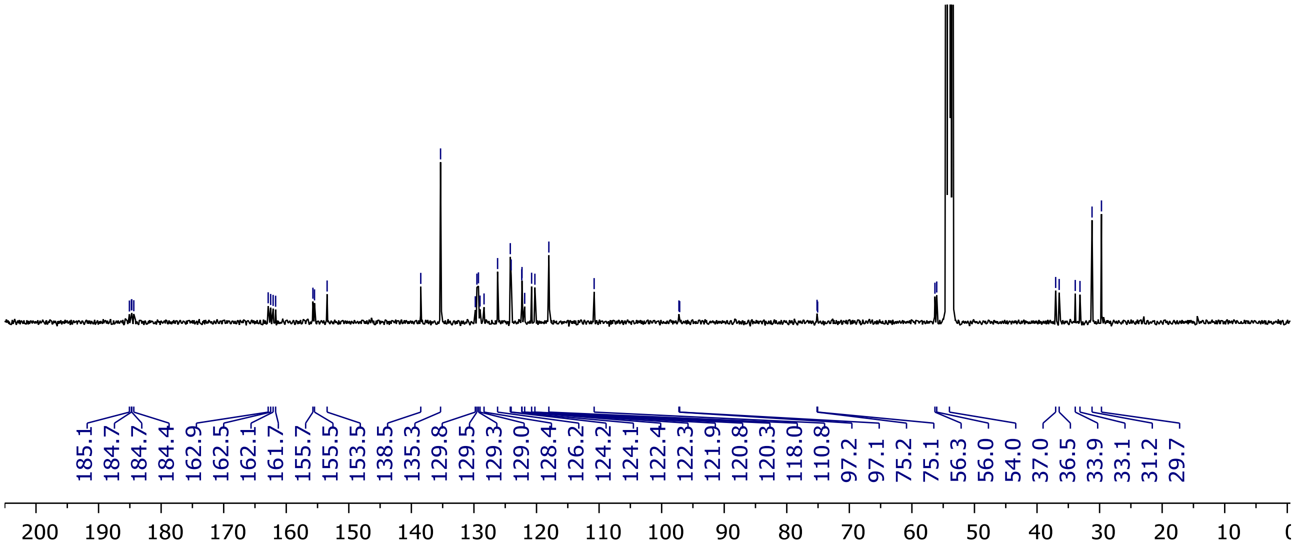

Figure S43: $^{13}C\{^{1}H\}$ NMR spectrum of **5** (CD_2Cl_2, 126 MHz).

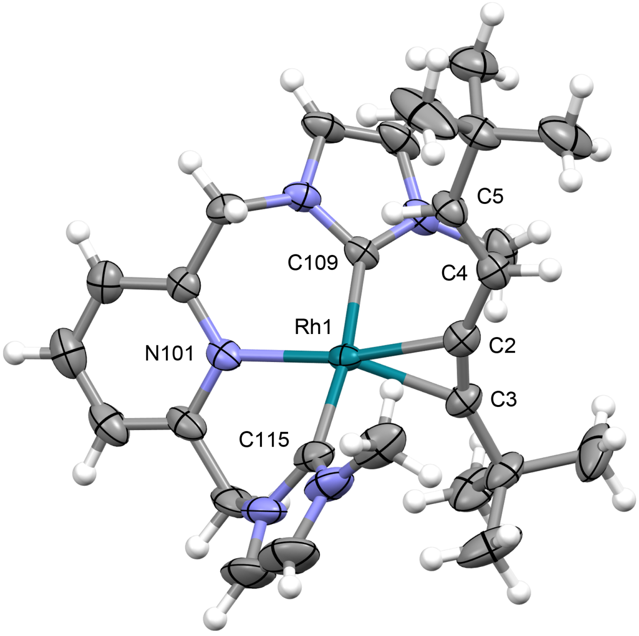

Figure S44: Solid state structure of **5**. Thermal ellipsoids drawn at 50% probability level; anion and CH_2Cl_2 solvent omitted for clarity. Selected data: Rh1–Cnt(C2,C3), 2.006(3) Å; N101–Rh1–Cnt(C2,C3), 173.31(11)°; C2–C3, 1.245(6) Å; C2–C4, 1.442(6) Å; C4–C5, 1.328(6) Å, Rh1–N101, 2.113(3) Å; Rh1–C109, 2.048(4) Å; Rh1–C115, 2.025(4) Å; C109–Rh1–C115, 172.43(17)°; py–Rh–C≡C twist 28.5(3)°.

3. NMR scale reaction of $1{\cdot}C_2H_4$ with 2-ethynylpyridine

2-Ethynylpyridine (2.2 µL, 22 µmol) was added to a solution of **1**·C_2H_4 (12.6 mg, 9.99 µmol) in DFB (0.50 mL) within a J. Young's valve NMR tube giving an immediate colour change from red to pale yellow. The solution was heated at 65 °C and monitored periodically by ^{1}H NMR spectroscopy. Quantitative conversion to **7** was observed within 18 h.

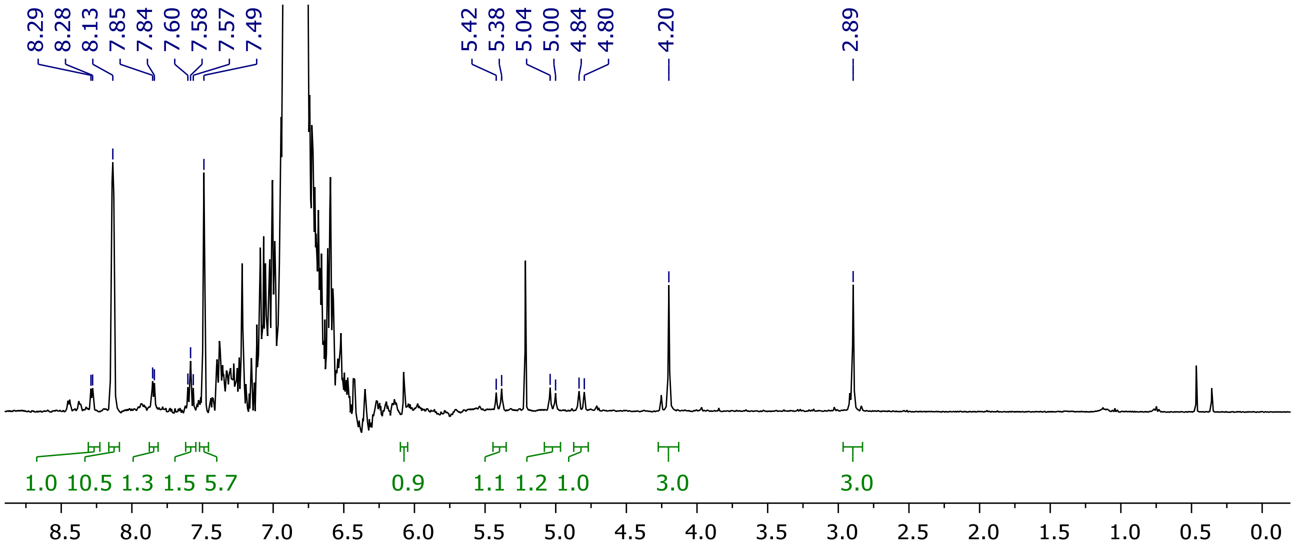

Figure S45: ^{1}H NMR spectrum collected during the reaction between **1**·C_2H_4 and 2-ethynylpyridine (*t* = 18 h, DFB, 400 MHz).

4. Synthesis of $[Rh(CNC\text{-}Me)(C{\equiv}Cpy)\{pyC(CH_2)\}][BAr^F{}_4]$ 7

A stirred solution of **1**·C_2H_4 (38.2 mg, 0.0303 mmol) and 2-ethynylpyridine (6.7 µL, 0.066 mmol) in DFB (2 mL) was heated at 65 °C for 18 h. Volatiles were removed *in vacuo* and the product obtained following recrystallisation from FB/hexane at room temperature. Yield: 35.6 mg (82%). Crystals suitable for X-ray diffraction were grown from a CH_2Cl_2/hexane at -30 °C.

^{1}H NMR (500 MHz, DFB, selected data): *δ* 8.28 (d, $^3J_{HH}$ = 4.9, 1H, C≡Cpy), 8.10 – 8.16 (m, 8H, Ar^F), 7.84 (d, $^3J_{HH}$ = 5.2, 1H, pyC(CH_2)), 7.57 (t, $^3J_{HH}$ = 7.7, 1H, pyCH_2), 7.48 (br, 4H, Ar^F), 6.52 (s, 1H, NCH), 6.43 (app. d, *J* = 4, 1H, pyC(CH_2)), 6.34 (s, 1H, NCH), 6.07 (br, 1H, pyC(CH_2)), 5.40 (d, $^2J_{HH}$ = 15.3, 1H, pyCH_2), 5.02 (d, $^2J_{HH}$ = 15.3, 1H, pyCH_2), 4.82 (d, $^2J_{HH}$ = 15.0, 1H, pyCH_2), 4.20 (s, 3H, NCH_3), 2.89 (s, 3H, NCH_3).

^{1}H NMR (600 MHz, CD_2Cl_2): *δ* 8.32 (d, $^3J_{HH}$ = 4.9, 1H, C≡Cpy), 8.02 (d, $^3J_{HH}$ = 5.2, 1H, pyC(CH_2)), 7.95 (t, $^3J_{HH}$ = 7.7, 1H, pyCH_2), 6.69 – 7.74 (m, 9H, Ar^F + pyC(CH_2)), 7.60 (d, $^3J_{HH}$ = 7.6, 1H, pyCH_2), 7.58 (d, $^3J_{HH}$ = 7.8, 1H, pyCH_2), 7.56 (br, 4H, Ar^F), 7.46 (t, $^3J_{HH}$ = 7.7, 1H, C≡Cpy), 7.40 (d, $^3J_{HH}$ = 8.1, 1H, pyC(CH_2)), 7.16 (s, 1H, NCH), 7.15 (t, $^3J_{HH}$ = 7.0, 1H, pyC(CH_2)), 7.10 (s, 1H, NCH), 7.08 (d, $^3J_{HH}$ = 8.1, 1H, C≡Cpy), 6.94 – 6.99 (m, 2H, C≡Cpy + pyCH_2), 6.90 (s, 1H, NCH), 6.79 (s, 1H, NCH), 6.48 (app. d, *J* = 4, 1H, pyC(CH_2)), 5.97 (d, $^2J_{HH}$ = 1.9, 1H, pyC(CH_2)), 5.38 (d, $^2J_{HH}$ = 15.3, 1H, pyCH_2), 5.13 (d, $^2J_{HH}$ = 15.3, 1H, pyCH_2), 5.06 (d, $^2J_{HH}$ = 15.1, 1H, pyCH_2), 4.19 (s, 3H, NCH_3), 3.07 (s, 3H, NCH_3).

$^{13}C\{^{1}H\}$ NMR (126 MHz, CD_2Cl_2): δ 173.0 (d, $^{2}J_{RhC}$ = 5, pyC(CH_2)), 172.5 (d, $^{1}J_{RhC}$ = 37, NCN), 171.8 (d, $^{1}J_{RhC}$ = 37, NCN), 162.3 (q, $^{1}J_{CB}$ = 50, Ar^F), 157.1 (s, pyCH_2), 155.3 (s, pyCH_2), 149.9 (s, C≡Cpy), 149.1 (s, pyC(CH_2)), 146.7 (s, C≡Cpy), 140.6 (s, pyCH_2), 138.1 (s, pyC(CH_2)), 137.0 (d, $^{1}J_{RhC}$ = 25, pyC(CH_2)), 136.0 (s, C≡Cpy), 135.3 (br, Ar^F), 129.4 (qq, $^{2}J_{FC}$ = 32, $^{3}J_{CB}$ = 3, Ar^F), 126.2 (s, C≡Cpy), 125.5 (s, pyCH_2), 125.2 (s, pyCH_2), 125.1 (q, $^{1}J_{FC}$ = 272, Ar^F), 124.4 (NCH), 124.29 (s, NCH), 124.26 (s, pyC(CH_2)), 122.3 (s, NCH), 121.8 (s, NCH), 120.7 (s, C≡Cpy), 119.8 (s, pyC(CH_2)), 118.0 (sept, $^{3}J_{FC}$ = 4, Ar^F), 114.4 (s, pyC(CH_2)), 106.4 (d, $^{2}J_{RhC}$ = 12, C≡Cpy), 98.8 (d, $^{1}J_{RhC}$ = 56, C≡Cpy), 56.5 (s, pyCH_2), 55.5 (s, pyCH_2), 39.0 (s, NCH_3), 36.4 (s, NCH_3).

HR ESI-MS (positive ion, 4 kV): 576.1373 ($[M]^+$, calcd 576.1377) *m*/z.

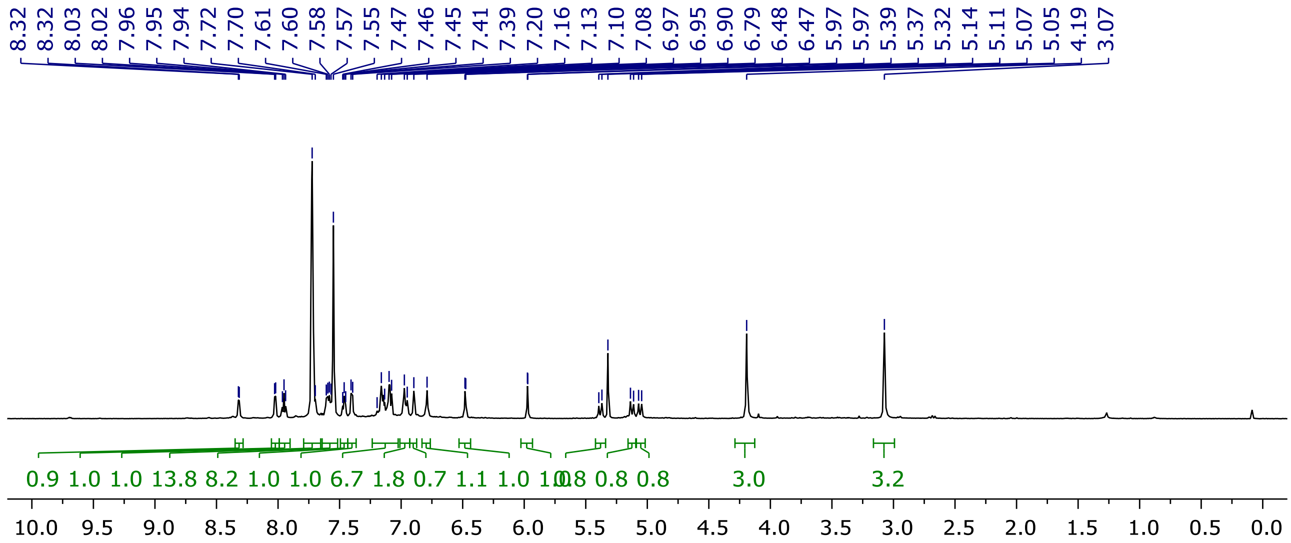

Figure S46: ^{1}H NMR spectrum of **7** (CD_2Cl_2, 600 MHz).

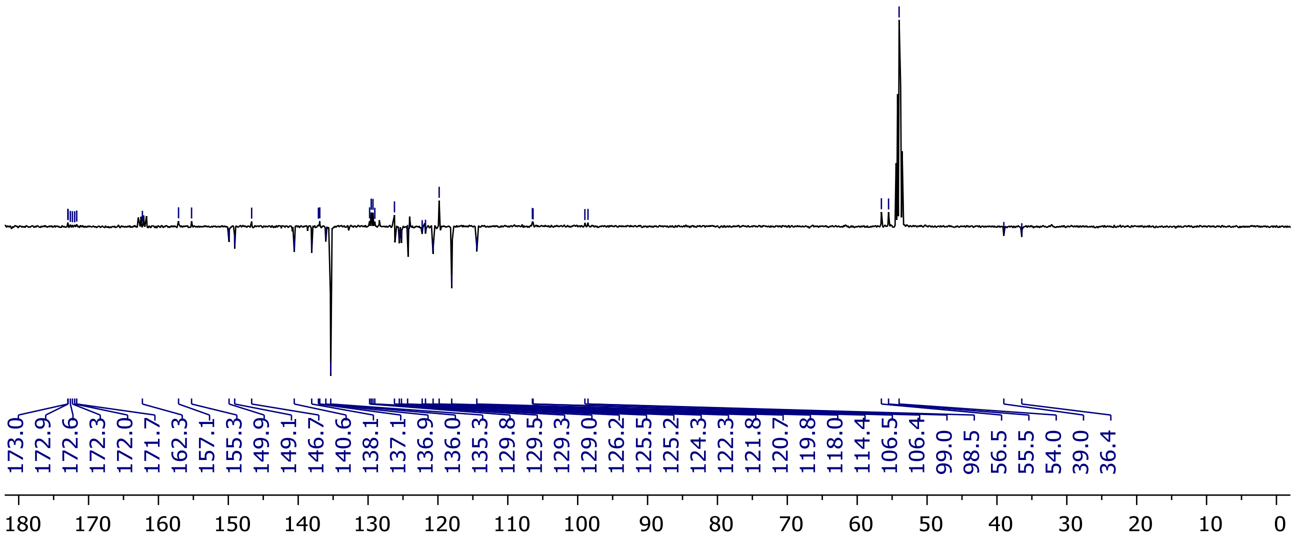

Figure S47: $^{13}C\{^{1}H\}$ APT NMR spectrum of **7** (CD_2Cl_2, 126 MHz).

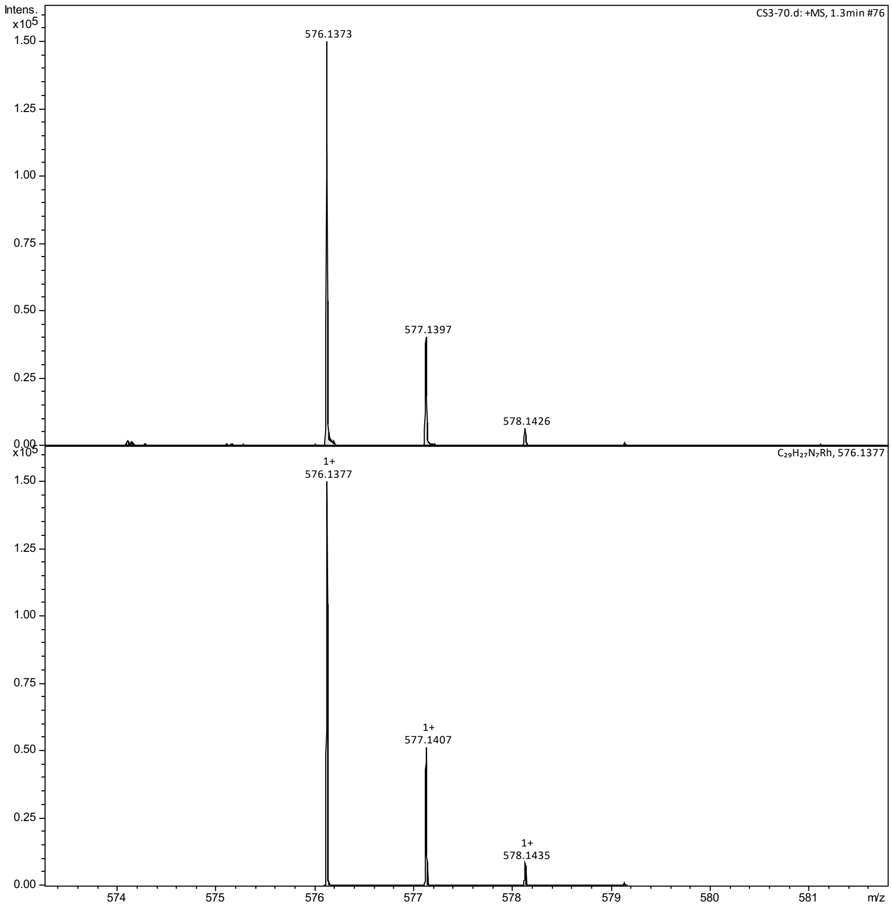

Figure S48 HR ESI-MS of **7**.

5. NMR scale reaction of 1·C_2H_4 with NBD

Norbornadiene (1.1 µL, 0.01 mmol) was added to a solution of **1**·C_2H_4 (12.6 mg, 0.01 mmol) in DFB (0.5 mL) within a J. Young's valve NMR tube. The reaction was mixed *via* inversion (*ca.* 30 rpm) at RT and monitored periodically by ^{1}H NMR spectroscopy. Quantitative conversion to **8** was observed within 2.5 h.

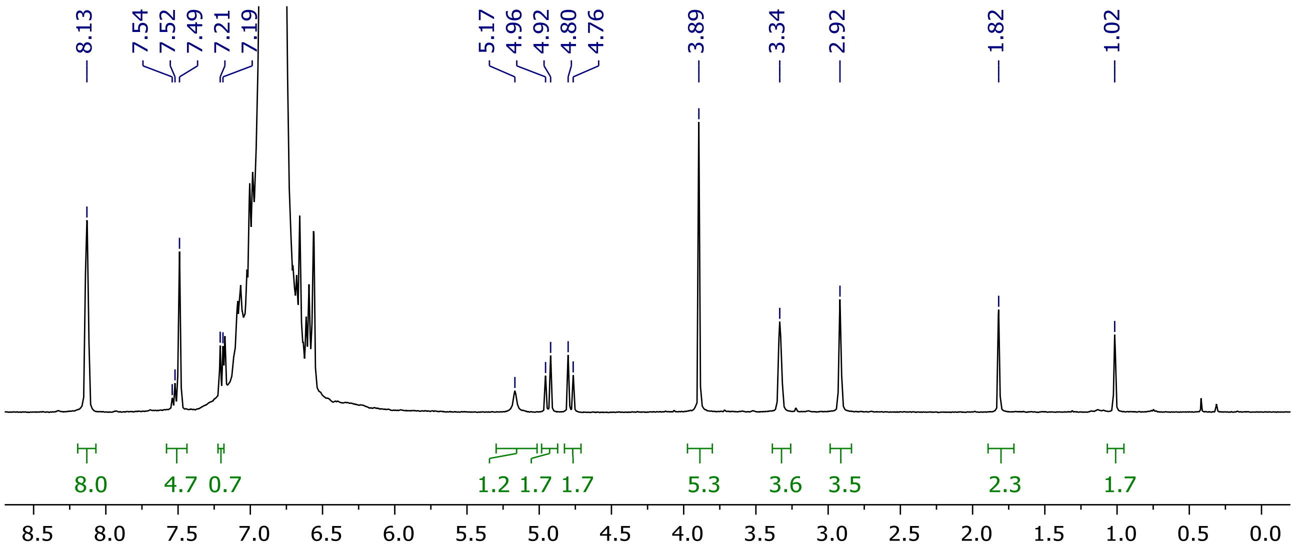

Figure S49: ^{1}H NMR spectrum collected during the reaction between **1**·C_2H_4 and NBD (*t* = 2.5 h, DFB, 400 MHz).

6. Synthesis of [Rh(CNC-Me)(NBD)][BAr$^F_4$] 8

Norbornadiene (4 µL, 0.04 µmol) was added to a stirred solution of **1**·C_2H_4 (45 mg, 0.036 µmol) in DFB (4 mL). After 10 minutes, volatiles were removed in vacuo and the resulting yellow residue washed with pentane (2 mL) and then recrystallised from a CH_2Cl_2/pentane at 18 °C to afford the product as a yellow crystalline solid. Yield: 35.1 mg (76%). Crystals grown in this way were suitable for X-ray diffraction.

^{1}H NMR (400 MHz, DFB): δ 8.10 – 8.16 (m, 8H, Ar^F), 7.52 (t, $^3J_{HH}$ = 7.8, 1H, py), 7.49 (br, 4H, Ar^F), 7.20 (d, $^3J_{HH}$ = 7.8, 2H, py), 6.66 (br, 2H, NCH), 6.56 (br, 2H, NCH), 4.94 (d, $^2J_{HH}$ = 14.0, 2H, pyC<u>H</u>$_2$), 4.78 (d, $^2J_{HH}$ = 14.1, 2H, pyC<u>H</u>$_2$), 3.89 (s, 6H, NCH_3), 3.33 (br, 2H, NBD{CH}), 2.92 (br, 4H, NBD{CH=CH}), 1.02 (s, 2H, NBD{CH_2}).

^{1}H NMR (500 MHz, CD_2Cl_2): δ 7.82 (t, $^3J_{HH}$ = 7.7, 1H, py), 7.70 – 7.76 (m, 8H, Ar^F), 7.56 (br, 4H, Ar^F), 7.45 (d, $^3J_{HH}$ = 7.7, 2H, py), 7.07 (d, $^3J_{HH}$ = 1.8, 2H, NCH), 6.90 (d, $^3J_{HH}$ = 1.8, 2H, NCH), 5.13 (d, $^2J_{HH}$ = 14.0, 2H, pyC<u>H</u>$_2$), 4.97 (d, $^2J_{HH}$ = 14.1, 2H, pyC<u>H</u>$_2$), 4.10 (s, 6H, NCH_3), 3.51 (br, 2H, NBD{CH}), 3.13 (br, 4H, NBD{CH=CH}), 1.16 (s, 2H, NBD{CH_2}).

^{13}C{^{1}H} NMR (126 MHz, CD_2Cl_2): δ 187.4 (d, $^1J_{RhC}$ = 51, NCN), 162.3 (q, $^1J_{CB}$ = 50, Ar^F), 157.0 (s, py), 139.6 (s, py), 135.4 (br, Ar^F), 129.4 (qq, $^2J_{FC}$ = 32, $^3J_{CB}$ = 3, Ar^F), 125.1 (q, $^1J_{FC}$ = 272, Ar^F), 123.9 (s, py), 123.1 (s, NCH), 122.6 (s, NCH), 118.0 (sept, $^3J_{FC}$ = 4, Ar^F), 60.9 (d, $^3J_{RhC}$ = 5, NBD{CH_2}), 56.5 (s, py<u>C</u>H$_2$), 48.3 (d, $^2J_{RhC}$ = 2, NBD{CH}), 43.6 (d, $^1J_{RhC}$ = 8, NBD{CH=CH}), 39.7 (s, NCH_3).

HR ESI-MS (positive ion, 4 kV): 462.1159 ([M]$^+$, 462.1160 calcd) *m/z*.

Anal. Calcd for $C_{54}H_{37}BF_{24}N_5Rh$ (1325.60 g·mol^{-1}): C, 48.93; H, 2.81; N, 5.28. Found: C, 49.04; H, 2.63; N, 5.30.

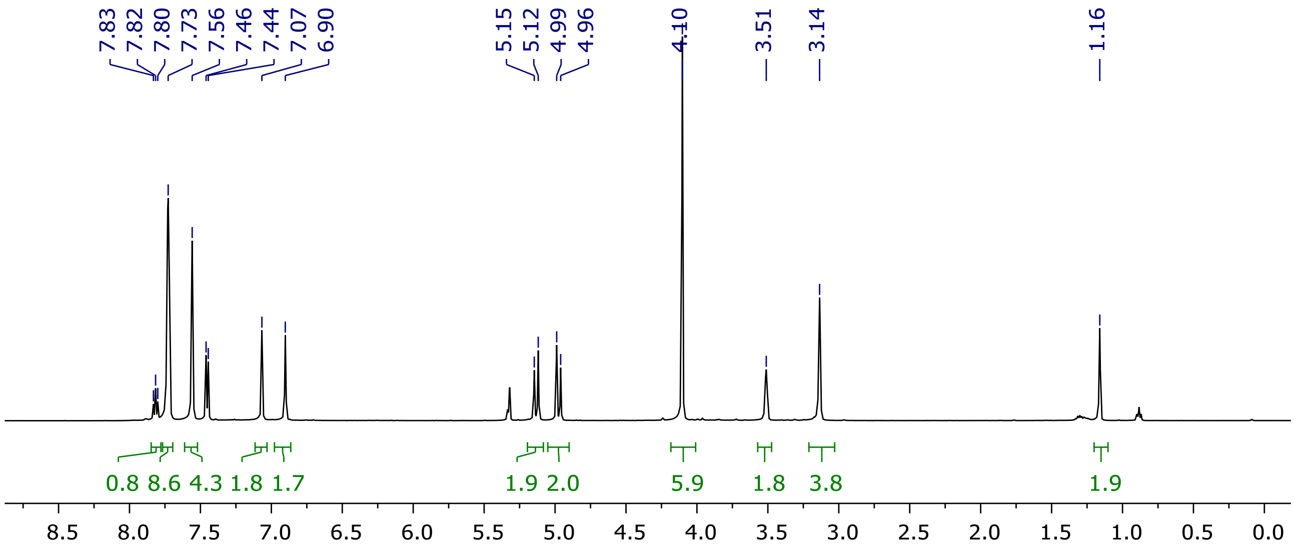

Figure S50: ^{1}H NMR spectrum of **8** (CD_2Cl_2, 500 MHz).

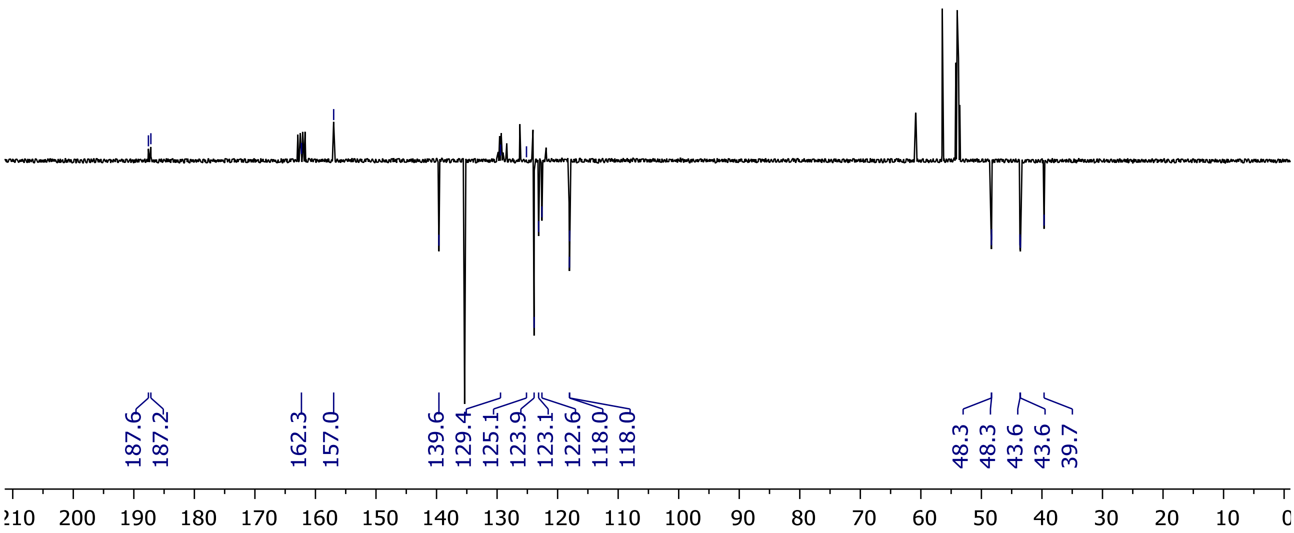

Figure S51: $^{13}C\{^{1}H\}$ APT NMR spectrum of **8** (CD_2Cl_2, 500 MHz).

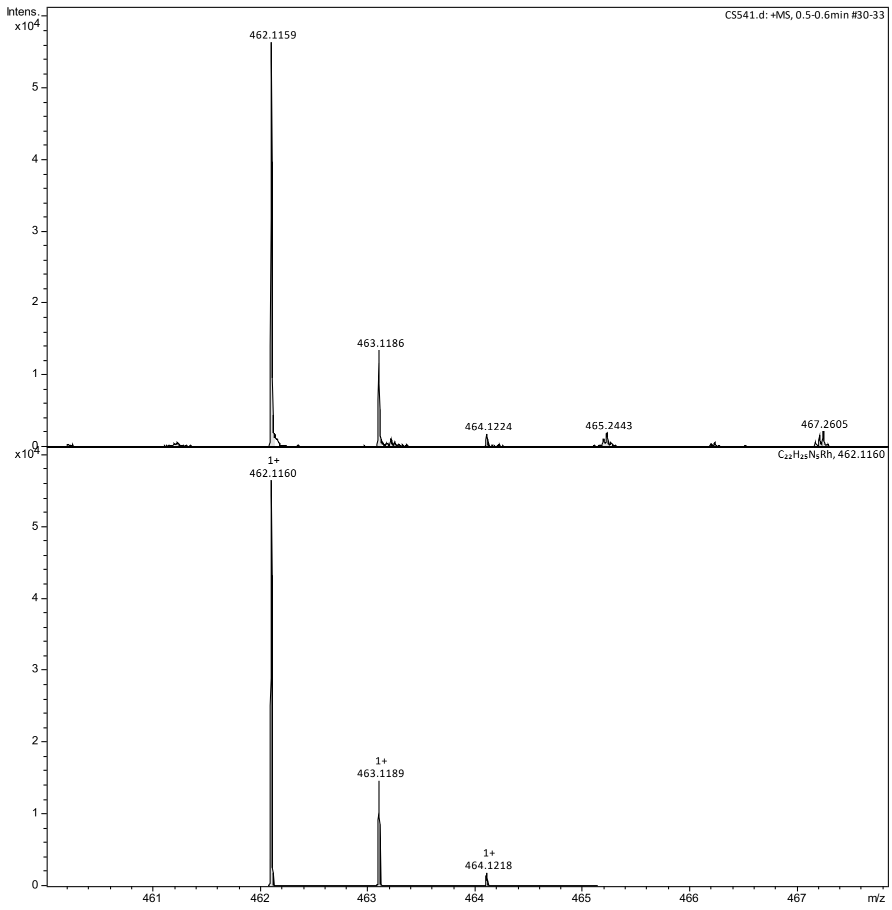

Figure S52: HR ESI-MS of **8.**

7. NMR scale reaction of 8 with CO

A solution of **8** (13 mg, 0.01 mmol) in DFB (0.5 mL) within a J. Young's valve NMR tube was freeze-pump-thaw degassed and placed under an atmosphere of CO (1 atm). The reaction was mixed *via* inversion (*ca.* 30 rpm) at RT and monitored periodically by ^{1}H NMR spectroscopy. Quantitative conversion to **1**·CO was observed with 5 h. NMR data are in agreement with the literature.[2]

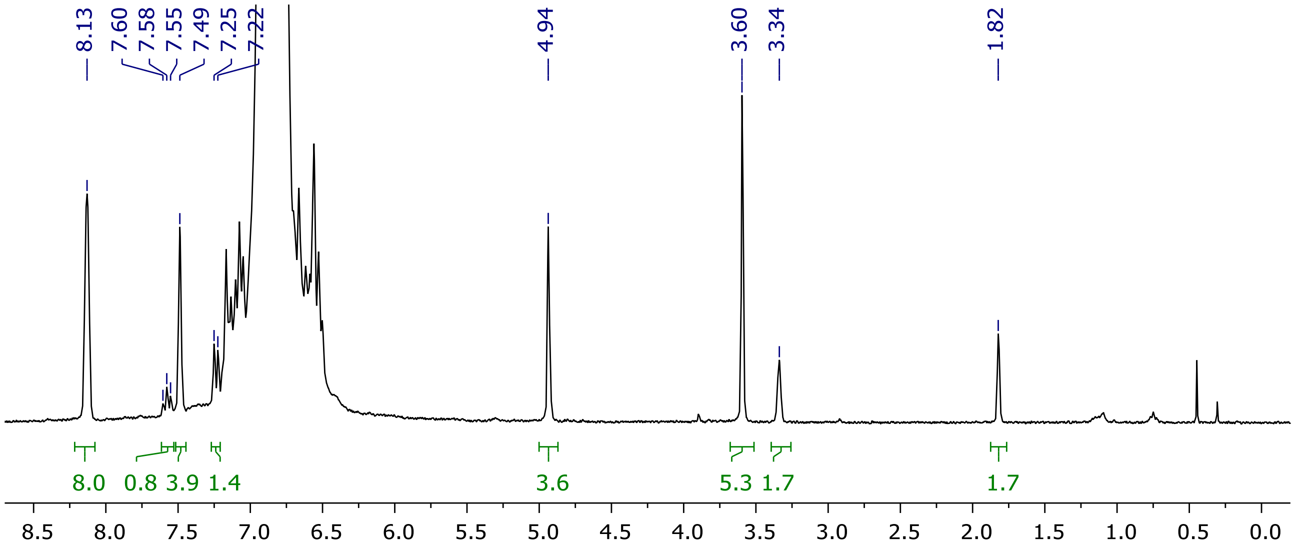

Figure S53: ^{1}H NMR spectrum collected during the reaction between **8** and CO (*t* = 5 h, DFB, 300 MHz).

8. NMR scale reaction of $1{\cdot}C_2H_4$ with di(3-phenylprop-2-ynyl)ether

A solution of $\mathbf{1}{\cdot}C_2H_4$ (10.2 mg, 8.09 µmol) and di(3-phenylprop-2-ynyl)ether (2.0 mg, 8.1 µmol) in DFB (0.5 mL) within a J. Young's valve NMR tube was mixed to afford a green solution. Analysis by ^{1}H NMR spectroscopy indicated quantitative conversion to **9** within 30 min.

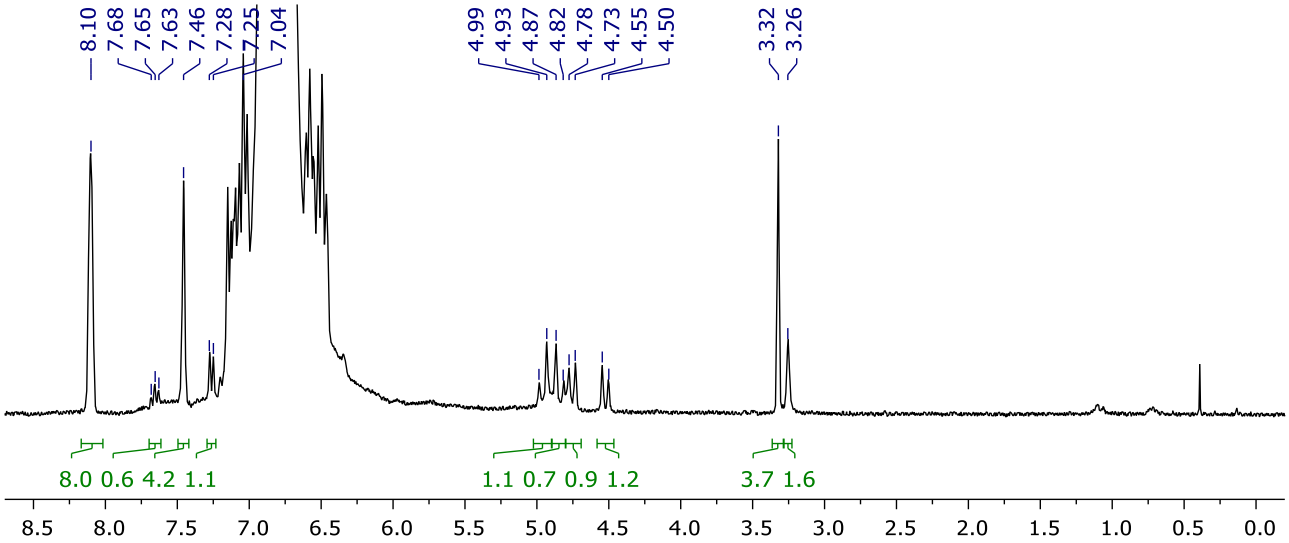

Figure S54: ^{1}H NMR spectrum collected during the reaction between $\mathbf{1}{\cdot}C_2H_4$ and di(3-phenylprop-2-ynyl)ether (t = 0.5 h, DFB, 300 MHz).

9. Synthesis of [Rh(CNC-Me)($Ph_2C_6H_4O$)][BAr$^F_4$] 9

A solution of $\mathbf{1}{\cdot}C_2H_4$ (12.6 mg, 0.01 mmol) and di(3-phenylprop-2-ynyl)ether (2.5 mg, 0.01 mmol) in DFB (0.5 mL) was stirred at RT for 1 h to afford a green solution. Yield: 14.4 mg (97%). Crystals suitable for X-ray diffraction were grown from a DFB/hexane at 18 °C.

^{1}H NMR (300 MHz, DFB, selected data): δ 8.10 – 8.17 (m, 8H, Ar^F), 7.69 (t, $^3J_{HH}$ = 7.7, 1H, py), 7.49 (br, 4H, Ar^F), 7.29 (d, $^3J_{HH}$ = 7.7, 2H, py), 4.99 (d, $^2J_{HH}$ = 15.7, 2H, pyC$\underline{H}_2$), 4.87 (d, $^2J_{HH}$ = 15.7, 2H, pyC$\underline{H}_2$), 4.79 (d, $^2J_{HH}$ = 12.8, 2H, OCH_2), 4.56 (d, $^2J_{HH}$ = 12.7, 2H, OCH_2), 3.36 (s, 6H, NCH_3).

^{1}H NMR (500 MHz, CD_2Cl_2): δ 7.87 (t, $^3J_{HH}$ = 7.7, 1H, py), 7.70 – 7.77 (m, 8H, Ar^F), 7.56 (br, 4H, Ar^F), 7.43 (d, $^3J_{HH}$ = 7.7, 2H, py), 7.16 (d, $^3J_{HH}$ = 1.8, 2H, NCH), 7.02 – 7.10 (m, 6H, *p*-Ph + *m*-Ph), 6.90 (d, $^3J_{HH}$ = 1.8, 2H, NCH), 6.54 (d, $^3J_{HH}$ = 6.1, 4H, *o*-Ph), 5.17 (d, $^2J_{HH}$ = 15.7, 2H, pyC$\underline{H}_2$), 4.98 (d, $^2J_{HH}$ = 15.7, 2H, pyC$\underline{H}_2$), 4.73 (d, $^2J_{HH}$ = 12.6, 2H, OCH_2), 4.54 (d, $^2J_{HH}$ = 12.7, 2H, OCH_2), 3.53 (s, 6H, NCH_3).

^{13}C{^{1}H} NMR (126 MHz, CD_2Cl_2): δ 175.9 (d, $^1J_{RhC}$ = 44, NCN), 162.3 (q, $^1J_{CB}$ = 50, Ar^F), 155.6 (s, py), 155.5 (d, $^2J_{RhC}$ = 3, $\underline{C}CH_2O$), 150.8 (d, $^1J_{RhC}$ = 41, Rh$\underline{C}$Ph), 145.1 (d, $^2J_{RhC}$ = 2, *i*-Ph), 141.2 (s, py), 135.4 (br, Ar^F), 129.4 (qq, $^2J_{FC}$ = 32, $^3J_{CB}$ = 3, Ar^F), 129.1 (s, *m*-Ph), 126.4 (s, *p*-Ph), 125.3 (s, py), 125.5 (s, *o*-Ph), 125.1 (q, $^1J_{FC}$ = 272, Ar^F), 124.6 (s, NCH), 122.3 (s, NCH), 118.1 (sept, $^3J_{FC}$ = 4, Ar^F), 68.0 (d, $^3J_{RhC}$ = 2, OCH_2), 55.7 (s, py$\underline{C}H_2$), 37.8 (s, NCH_3).

HR ESI-MS (positive ion, 4 kV): 616.1581 ([M]$^+$, 616.1578 calcd) *m/z*.

Anal. Calcd for $C_{65}H_{43}BF_{24}N_5ORh$ (1479.77 g·mol^{-1}): C, 52.76; H, 2.93; N, 4.73. Found: C, 52.87; H, 2.89; N, 4.63.

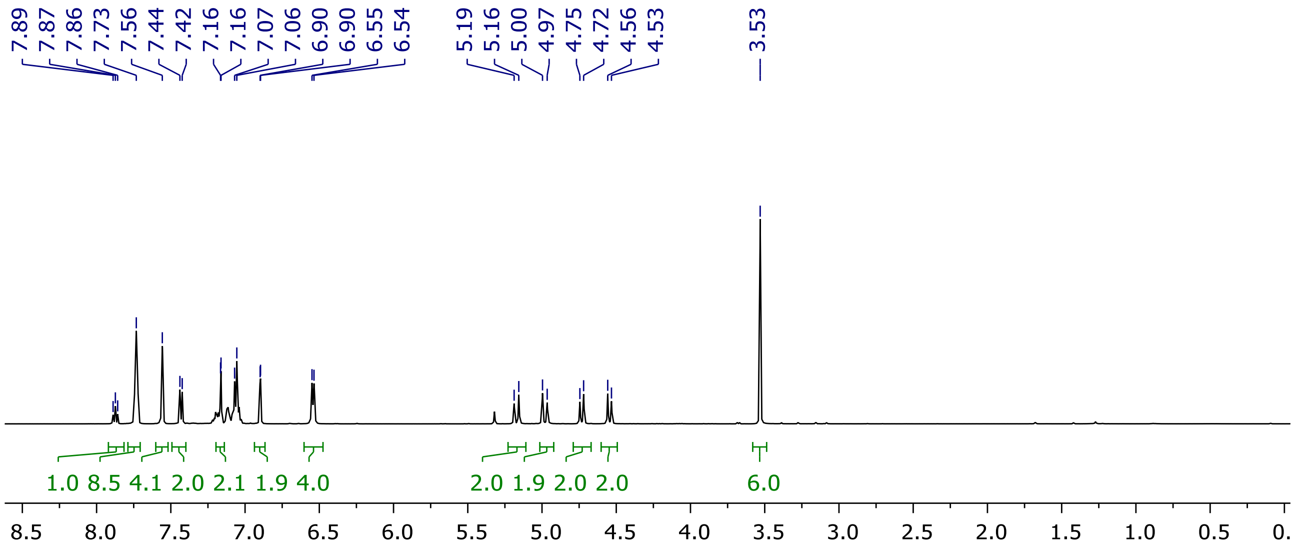

Figure S55: ^{1}H NMR spectrum of **9** (CD_2Cl_2, 500 MHz).

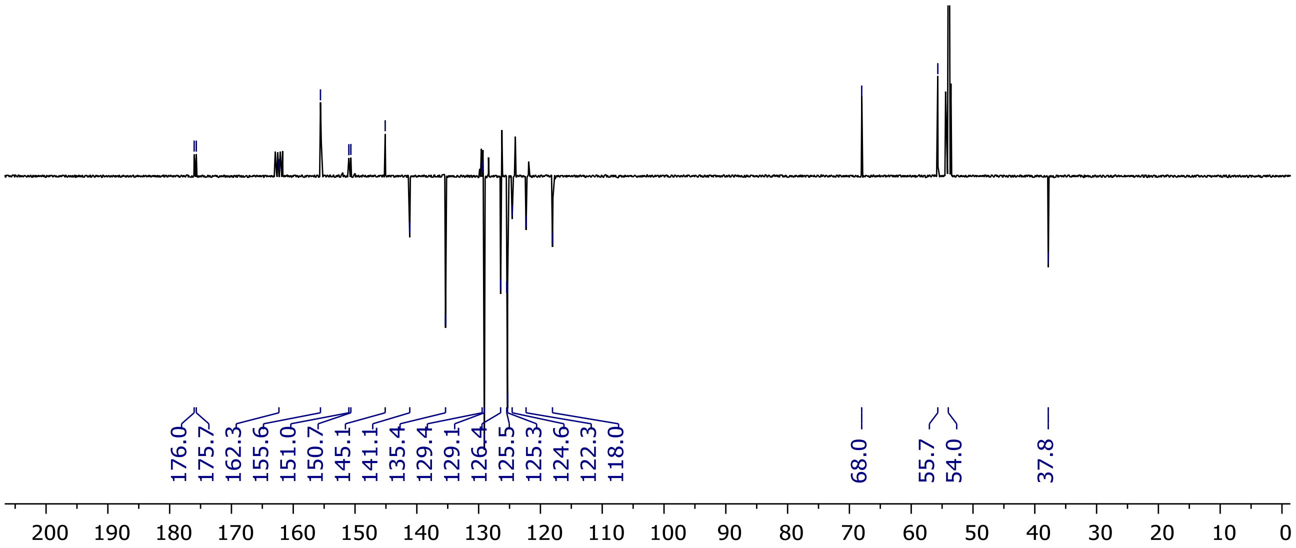

Figure S56: $^{13}C\{^{1}H\}$ APT NMR spectrum of **9** (CD_2Cl_2, 500 MHz).

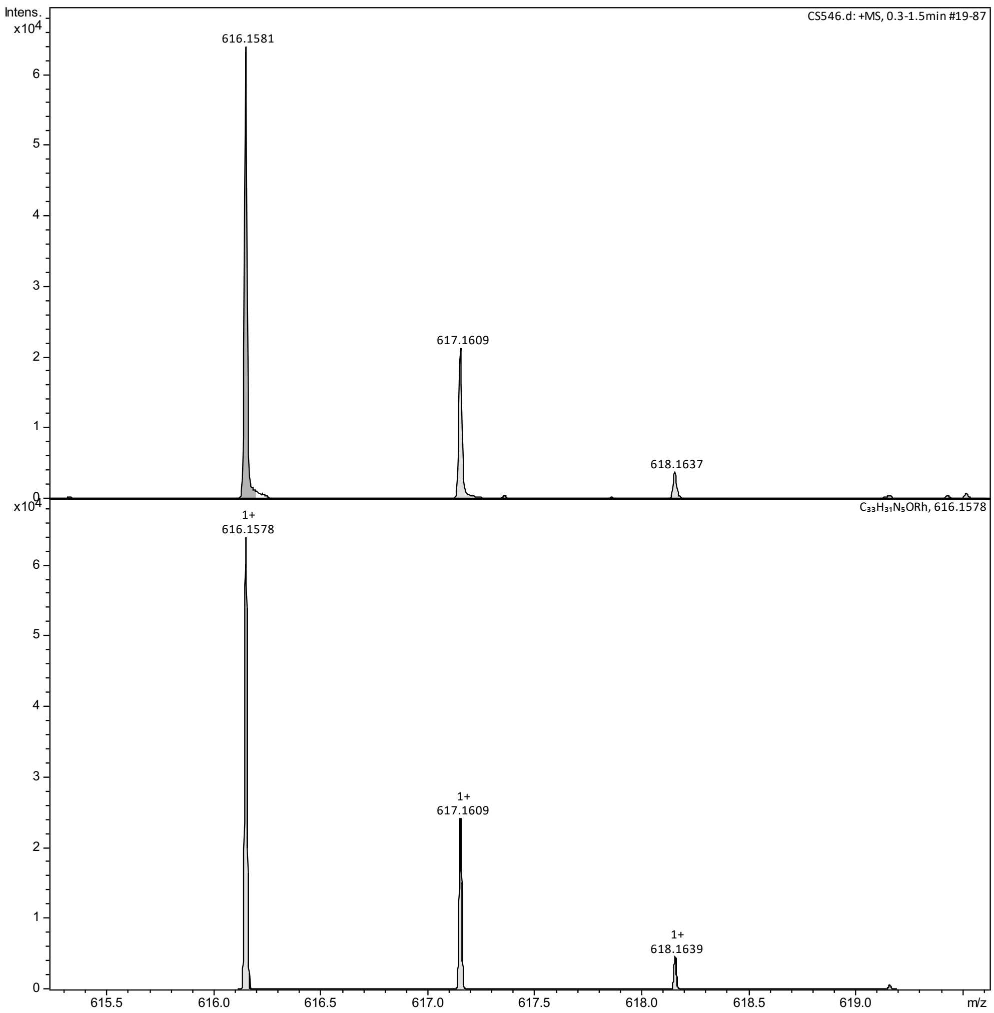

Figure S57: HR ESI-MS of **9.**

10. NMR scale thermolysis of 9 in DFB

A solution of **9** (11.0 mg, 7.44 µmol) in DFB (0.37 mL) within a J. Young's valve NMR tube was heated at 85 °C for 24 h and monitored periodically by ^{1}H NMR spectroscopy. No reaction was apparent.

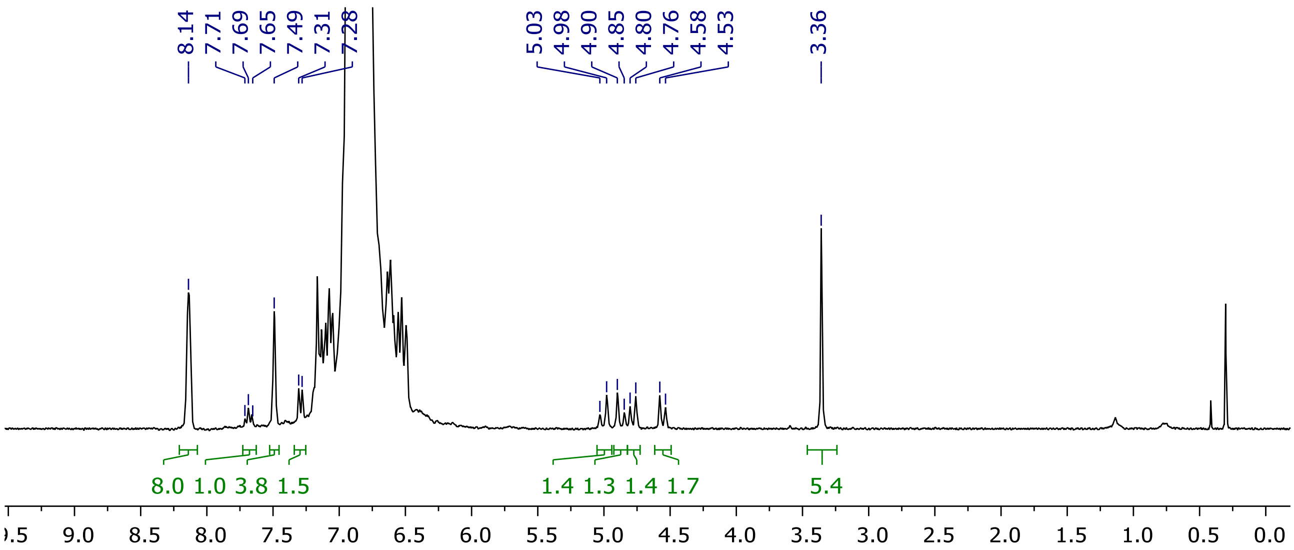

Figure S58: ^{1}H NMR spectrum collected during the thermolysis of **9** (t = 24 h, DFB, 300 MHz).

11. Organometallic chemistry of [Rh(CNC-12)(C_2H_4)][BAr^F$_4$] 6·C_2H_4

11.1. NMR scale reaction of 6·C_2H_4 with NBD

Norbornadiene (1.1 µL, 0.01 mmol) was added to a solution of **6**·C_2H_4 (14 mg, 0.01 mmol) in DFB (0.5 mL) within a J. Young's valve NMR tube. The reaction was mixed *via* inversion (*ca.* 30 rpm) at RT and monitored periodically by ^{1}H NMR spectroscopy. Quantitative conversion to [Rh(CNC-12)(NBD)][BAr^F$_4$] was observed with 2 h.

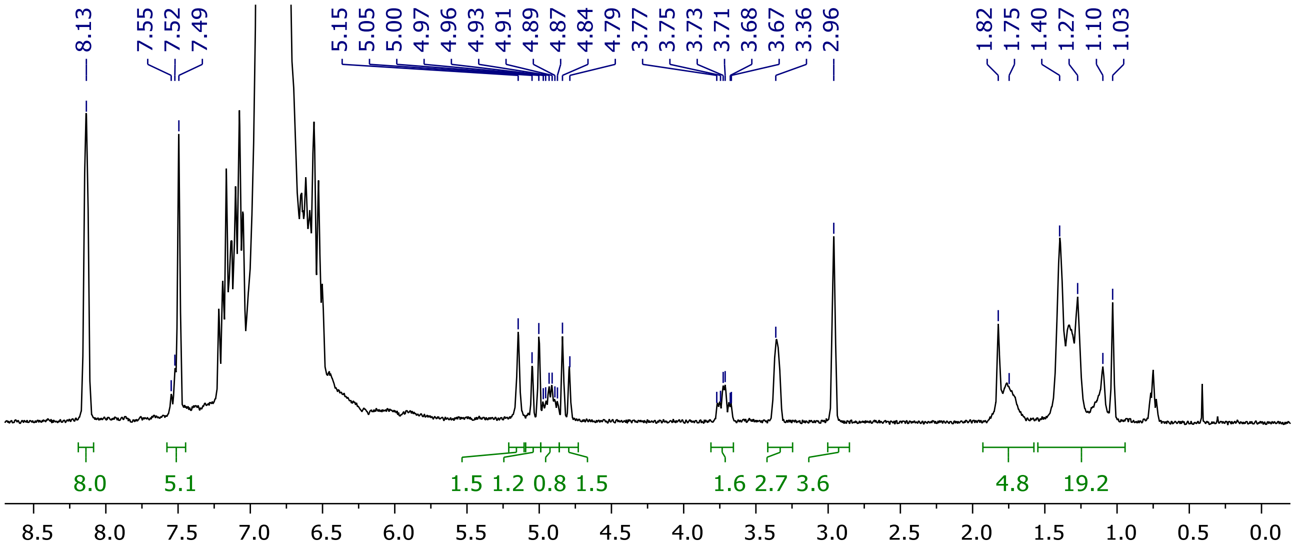

Figure S59: ^{1}H NMR spectrum collected during the reaction between **6**·C_2H_4 and NBD (t = 2 h, DFB, 300 MHz).

11.2. Synthesis of [Rh(CNC-12)(NBD)][BAr^F$_4$]

Norbornadiene (1.1 µL, 0.01 µmol) was added to a stirred solution of **6**·C_2H_4 (14 mg, 0.01 µmol) DFB (0.5 mL). The solution was stirred for 3 h, volatiles removed in vacuo and the resulting yellow residue washed with pentane (2 mL) and dried. Yield: 10.1 mg (69%). Crystals suitable for X-ray diffraction were grown from DFB/hexane at -30 °C.

^{1}H NMR (300 MHz, DFB, selected data): δ 8.10 – 8.17 (m, 8H, Ar^F), 7.52 (t, $^3J_{HH}$ = 7.7, 1H, py), 7.49 (br, 4H, Ar^F), 5.03 (d, $^3J_{HH}$ = 14.2, 2H, pyC<u>H</u>$_2$), 4.92 (td, $^2J_{HH}$ = 12.0, $^3J_{HH}$ = 6.2, 2H, NCH_2), 4.81 (d, $^3J_{HH}$ = 14.2, 2H, pyC<u>H</u>$_2$), 3.72 (td, $^2J_{HH}$ = 12.0, $^3J_{HH}$ = 4.9, 2H, NCH_2), 3.36 (br, 2H, NBD{CH}), 2.96 (br, 4H, NBD{CH=CH}), 1.75 (vbr, fwhm = 40, 4H, CH_2), 1.20 – 1.50 (m, 16H, CH_2), 1.03 (br, 2H, NBD{CH_2}).

^{1}H NMR (500 MHz, CD_2Cl_2): δ 7.82 (t, $^3J_{HH}$ = 7.7, 1H, py), 7.70 – 7.75 (m, 8H, Ar^F), 7.55 (br, 4H, Ar^F), 7.45 (d, $^3J_{HH}$ = 7.7, 2H, py), 7.10 (d, $^3J_{HH}$ = 1.9, 2H, NCH), 6.95 (d, $^3J_{HH}$ = 1.8, 2H, NCH), 5.19 (d, $^2J_{HH}$ = 14.1, 2H, pyC<u>H</u>$_2$), 5.04 (td, $^2J_{HH}$ = 12.2, $^3J_{HH}$ = 5.8, 2H, NCH_2), 4.99 (d, $^2J_{HH}$ = 14.1, 2H, pyC<u>H</u>$_2$), 3.92 (td, $^2J_{HH}$ = 12.2, $^3J_{HH}$ = 4.7, 2H,

NCH_2), 3.51 (br, 2H, NBD{CH}), 3.14 (br, 4H, NBD{CH=CH}), 1.94 – 2.05 (m, 2H, CH_2), 1.82 – 1.94 (m, 2H, CH_2), 1.42 – 1.63 (m, 16H, CH_2), 1.17 (br, 2H, NBD{CH_2}).

^{13}C{^{1}H} NMR (126 MHz, CD_2Cl_2): δ 186.6 (d, $^1J_{RhC}$ = 51, NCN), 162.3 (q, $^1J_{CB}$ = 50, Ar^F), 157.2 (s, py), 139.6 (s, py), 135.2 (br, Ar^F), 129.4 (qq, $^2J_{FC}$ = 32, $^3J_{CB}$ = 3, Ar^F), 125.1 (q, $^1J_{FC}$ = 272, Ar^F), 123.8 (s, py), 123.6 (s, NCH), 120.4 (s, NCH), 118.0 (sept, $^3J_{FC}$ = 4, Ar^F), 60.8 (d, $^3J_{RhC}$ = 5, NBD{CH_2}), 56.5 (s, py$\underline{C}H_2$), 52.2 (s, NCH_2), 48.4 (d, $^2J_{RhC}$ = 2, NBD{CH}), 43.1 (d, $^1J_{RhC}$ = 8, NBD{CH=CH}), 31.8 (s, CH_2), 26.9 (s, CH_2), 26.7 (s, CH_2), 26.0 (s, CH_2), 24.9 (s, CH_2).

HR ESI-MS (positive ion, 4 kV): 600.2563 ([M]$^+$, 600.2568 calcd) *m/z*.

Anal. Calcd for $C_{64}H_{55}BF_{24}N_5Rh$ (1463.86 g·mol^{-1}): C, 52.51; H, 3.79; N, 4.78. Found: C, 52.64; H, 3.72; N, 4.65.

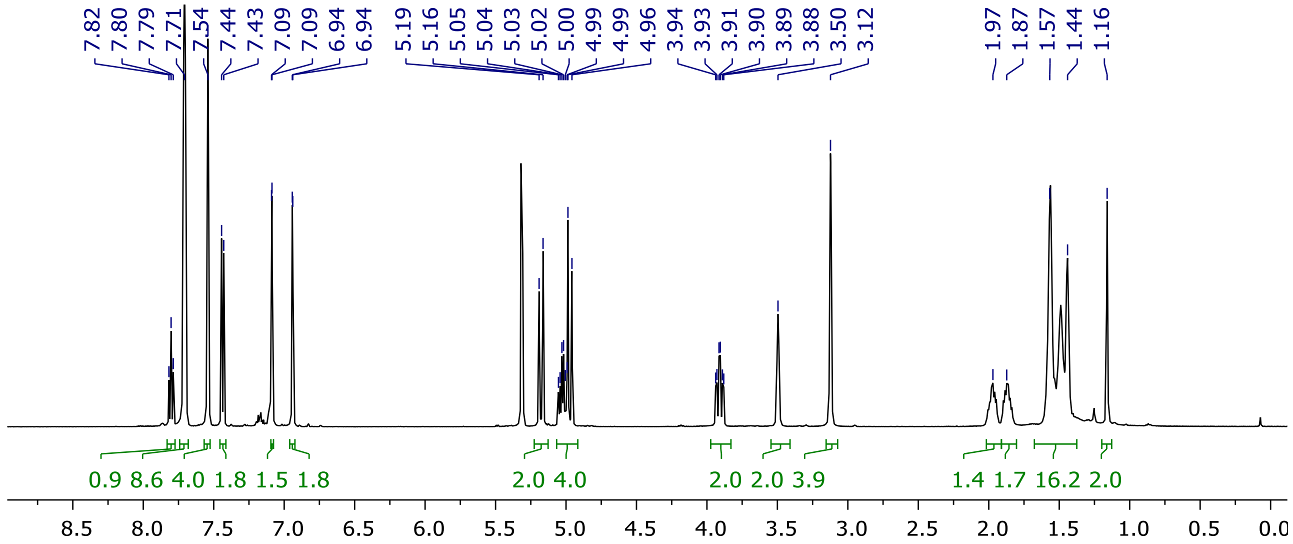

Figure S60: ^{1}H NMR spectrum of [Rh(CNC-12)(NBD)][BAr$^F_4$] (CD_2Cl_2, 500 MHz).

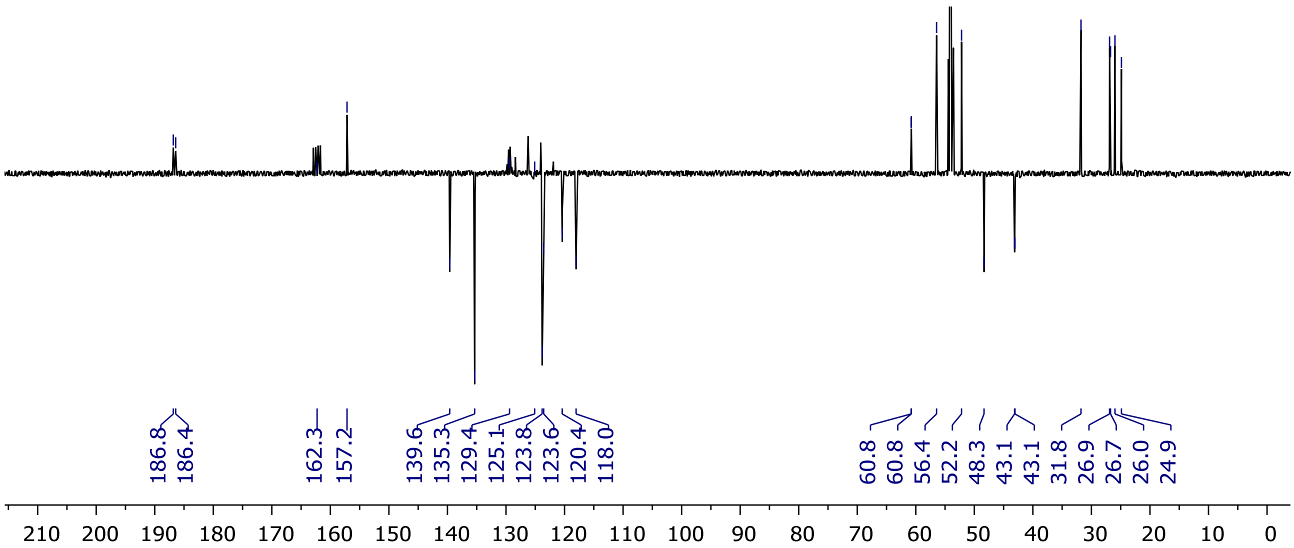

Figure S61{^{1}H} APT NMR spectrum of [Rh(CNC-12)(NBD)][BAr$^F_4$] (CD_2Cl_2, 500 MHz).

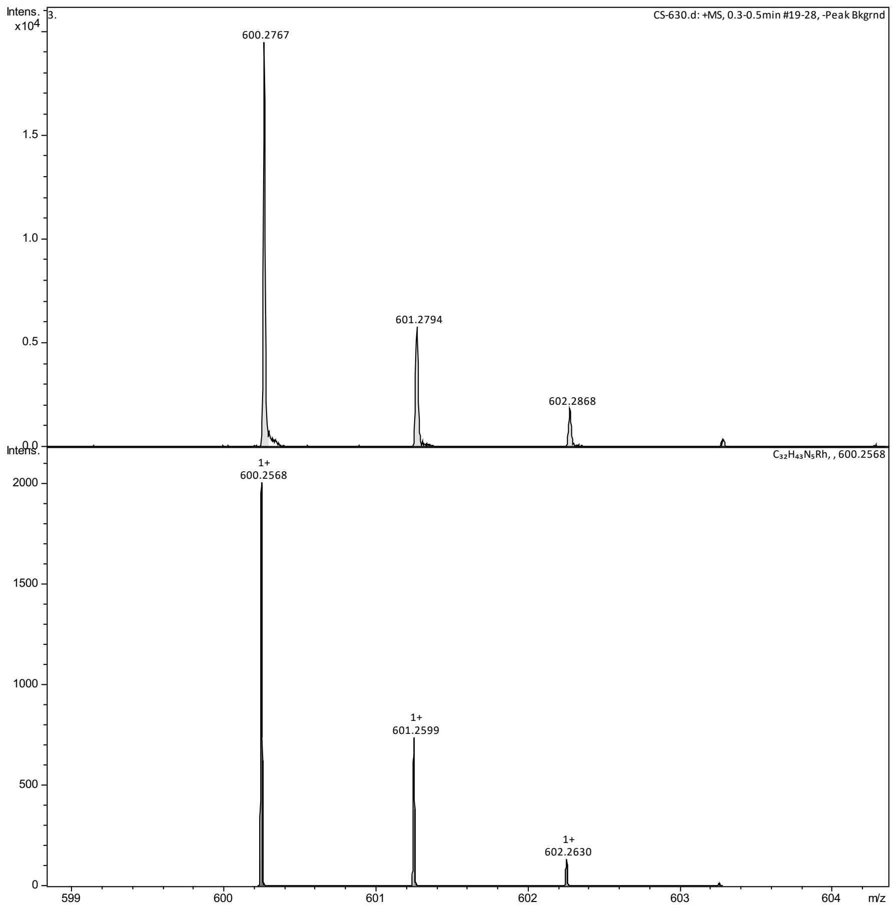

Figure S62: HR ESI-MS of [Rh(CNC-12)(NBD)][BAr$^F{}_4$].

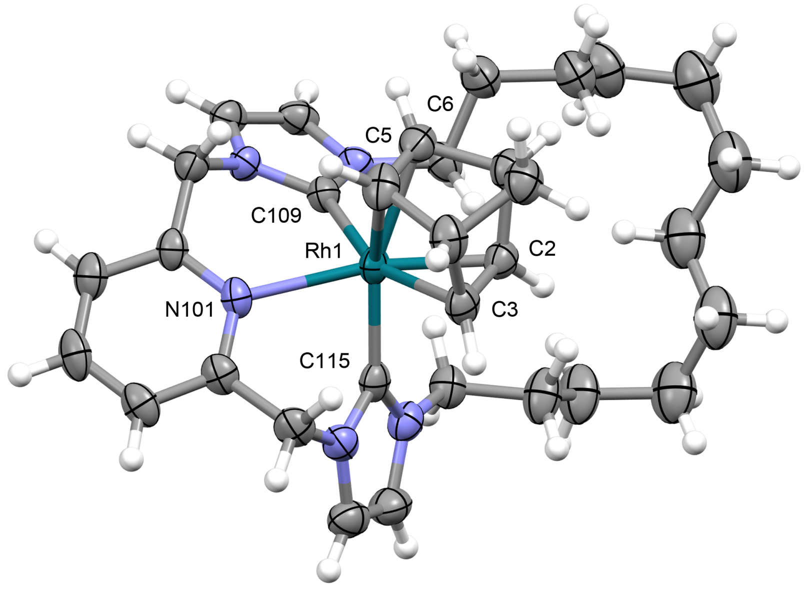

Figure S63: Solid state structure of [Rh(CNC-12)(NBD)][BAr$^F{}_4$]. Thermal ellipsoids drawn at 50% probability level; anion and hexane solvent omitted for clarity. Selected data: Rh1–Cnt(C2,C3), 1.965(2) Å; Rh1–Cnt(C5,C6), 2.135(3) Å; C2–C3, 1.435(6) Å; C5–C6, 1.371(6) Å; N101–Rh1–Cnt(C2,C3), 136.69(12)°; C109–Rh1–Cnt(C2,C3), 138.46(12)°; C115–Rh1–Cnt(C5,C6), 160.00(12)°; Rh1–N101, 2.334(3) Å; Rh1–C109, 2.115(4) Å; Rh1–C115, 2.041(3) Å; C109–Rh1–C115, 102.51(13)°.

11.3. NMR scale reaction of [Rh(CNC-12)(NBD)][BAr$^F_4$] with CO

A solution of [Rh(CNC-12)(NBD)][BAr$^F_4$] (10.1 mg, 0.007 mmol) in DFB (0.5 mL) within a J. Young's valve NMR tube was freeze-pump-thaw degassed and placed under an atmosphere of CO (1 atm). The reaction was mixed *via* inversion (*ca.* 30 rpm) at RT and monitored periodically by ^{1}H NMR spectroscopy. Quantitative conversion to **6**·CO was observed with 30 min. NMR data are in agreement with the literature.[2]

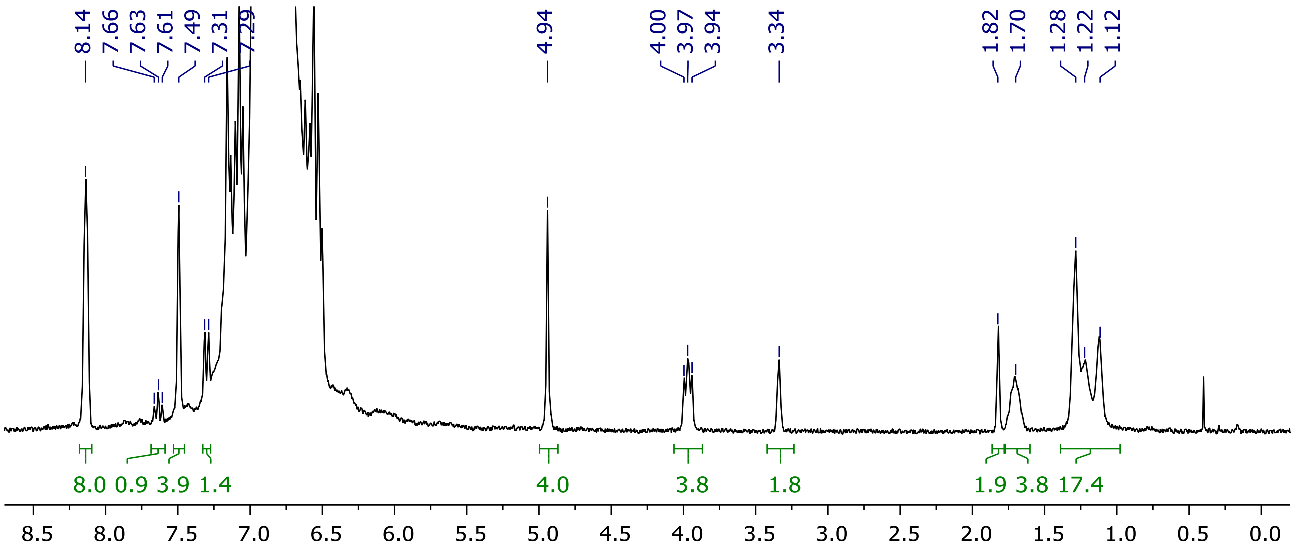

Figure S64: ^{1}H NMR spectrum collected during the reaction between [Rh(CNC-12)(NBD)][BAr$^F_4$] and CO (*t* = 30 min, DFB, 300 MHz).

11.4. Zipper annulation of Ar'C≡CC(CH$_2$)Ar' (3a) catalysed by 6·C_2H_4

A solution of **3a** (50 mM, 0.5 mL) in DFB was added to a cooled J. Young's valve NMR tube (-30 °C) charged with complex **6**·C_2H_4 (3.5 mg, 2.5 mmol) and the reaction mixture heated at 50 °C. The reaction was monitored periodically by ^{1}H NMR spectroscopy and quenched after 45 days by freeze-pump-thaw degassing and placing under an atmosphere of CO (1 atm). Using the CH_2 signals of **4a** and CH=CH signals of **3a**, 40% conversion of **3a** to **4a** was determined by integration (2 TON).

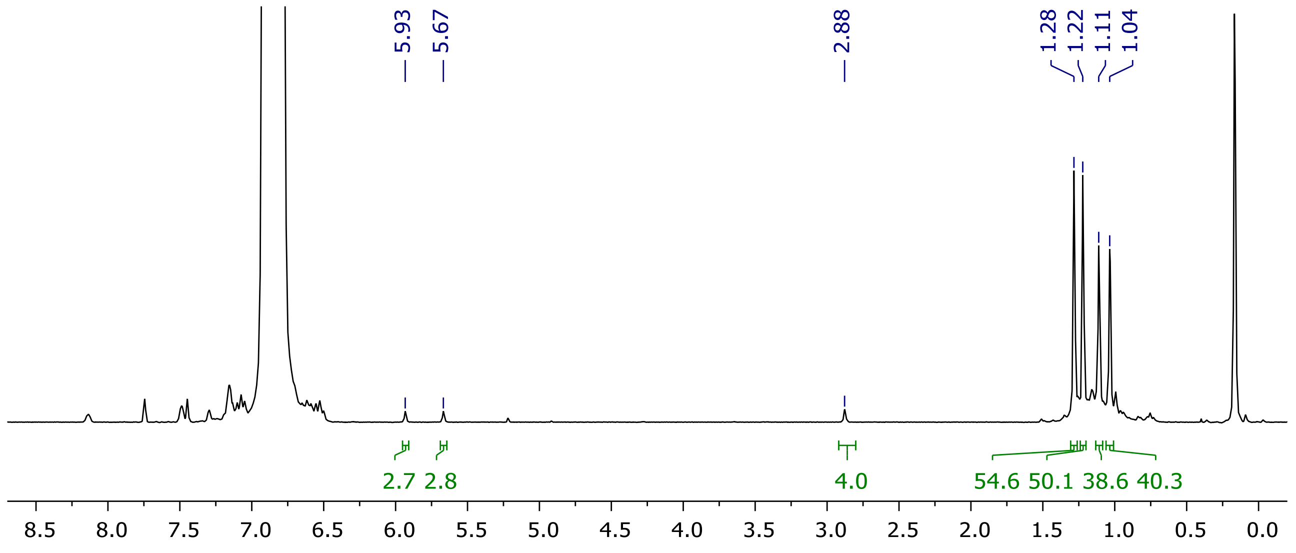

Figure S65: ^{1}H NMR spectrum collected after quenching (DFB, 300 MHz).

12. Computational methods

All electronic structure calculations presented in this paper were carried out using the Gaussian 09 (Revision E.01) program suite at the DFT level of theory.[11] Geometries of all complexes were fully optimized without imposing symmetry constraints (C_1 symmetry), employing the B3PW91 global hybrid GGA functional. B3PW91 combines Becke's three-parameter functional with 20% exact exchange and the non-local correlation provided by the Perdew 91 expression.[12] The Stuttgart-Dresden (SDD) relativistic effective core potential in combination with the associated basis sets were used to describe the Rh centre, augmented with an additional f-type polarization function (ζ = 1.350).[13,14] The 6-31G(d,p) basis sets developed by Pople and co-workers were used on all lighter atoms (C, N, F and H).[15] Optimized stationary points were characterized by analysis of their analytical second derivatives, with minima having only positive eigenvalues and transition states having exactly one imaginary eigenvalue. Geometries are provided in .xyz format.

In order to identify the minima linked by each transition state, subsequent geometry optimizations were performed in both forward and reverse direction of the displacement vector of the transition state coordinate. The frequency calculations also provided thermal and entropic corrections to the total energy in gas phase at T = 298.15 K and p = 1 atm within the rigid-rotor/harmonic oscillator (RRHO) approximation. Dispersion effects were accounted for by applying Grimme's van der Waals correction (D3 parameterization with Becke-Johnson damping) during geometry optimizations of all stationary points.[16,17] Effects due to the presence of a solvent were treated implicitly with a polarisable dielectric model, using the IEFPCM formalism in conjunction with Truhlar's SMD model.[18] In the absence of defined parameters for 1,2-difluorobenzene solvent, default SMD parameters were selected for fluorobenzene and the dielectric constant adjusted to that of 1,2-difluorobenzene (ε = 13.4). We note that similar results were obtained when 1,2-dichlorobenzene (ε = 9.99) was used as the solvent. All Gibbs energies are reported in kcal mol^{-1}. All structures were visualized using the Chemcraft tool.[19] Computational resources were provided by the Irish Centre for High-End Computing (ICHEC). All calculations were run on a single node (i.e. 40 cores) of the Kay supercomputer architecture, a cluster system of 336 nodes, where each node has 2×20-core 2.4 GHz Intel Xeon Gold 6148 (Skylake) processors, 192 GB of RAM, a 400 GB local SSD scratch allocation and a 100 Gbit OmniPath network adaptor.

13. Homocoupling of PhC≡CH (2b)

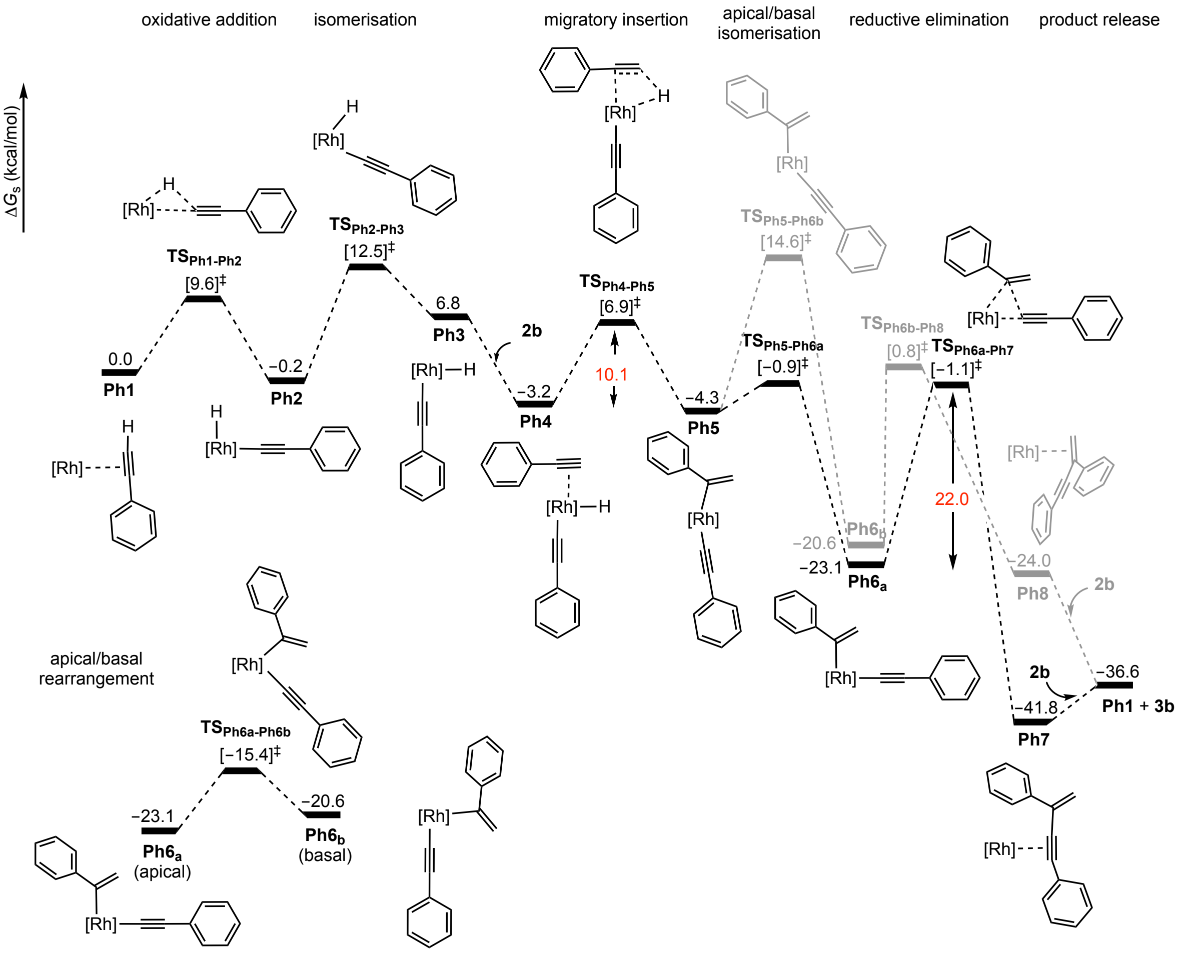

Figure S66: Calculated reaction profile (B3PW91-D3/SDD/6-31G**) for the head-to-tail homocoupling of **2b** affording **3b** via the hydrometallation mechanism. Relative Gibbs free energies (kcal mol^{-1}) are corrected for DFB solvent.

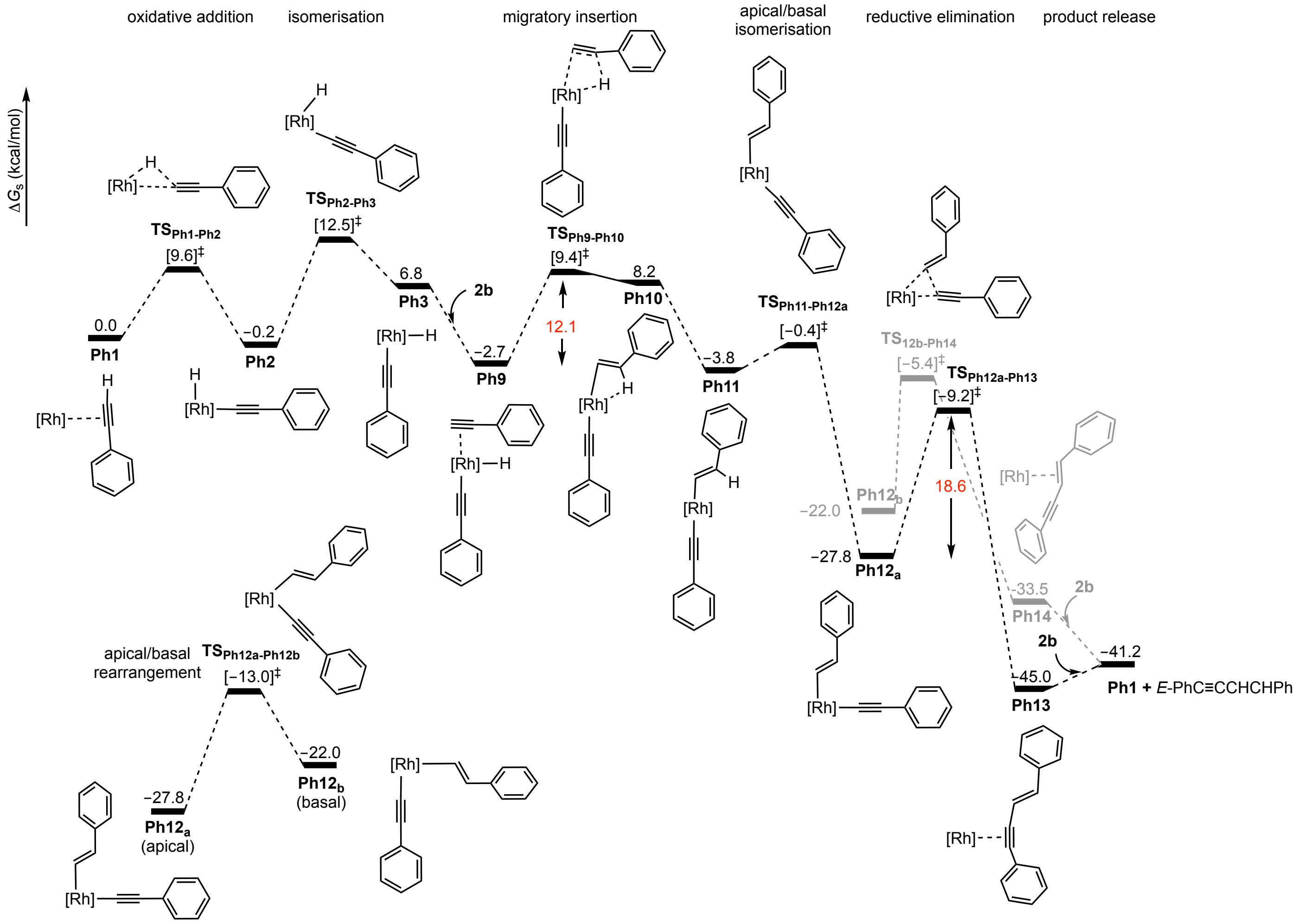

Figure S67: Calculated reaction profile (B3PW91-D3/SDD/6-31G**) for the head-to-head homocoupling of **2b** affording *E*-PhC≡CCHCHPh via the hydrometallation mechanism. Relative Gibbs free energies (kcal mol^{-1}) are corrected for DFB solvent.

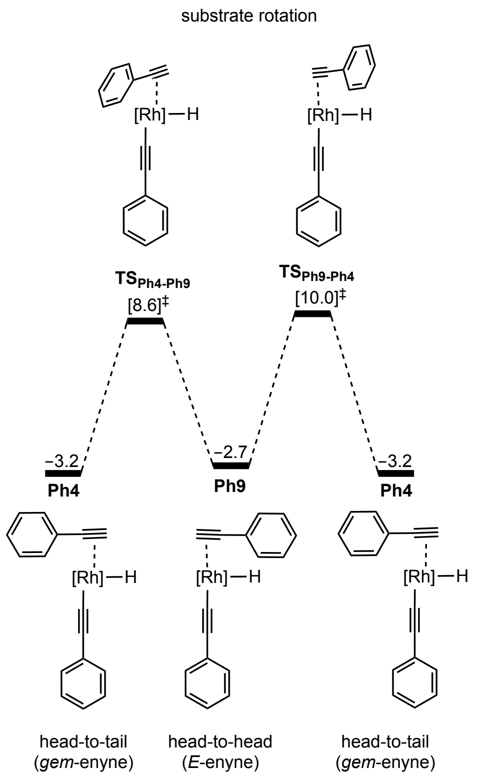

Figure S68: Calculated reaction profile (B3PW91-D3/SDD/6-31G**) for the interchange between **Ph4** and **Ph9** via substrate rotation, associated with the homocoupling of **2b**. Relative Gibbs free energies (kcal mol^{-1}) are corrected for DFB solvent.

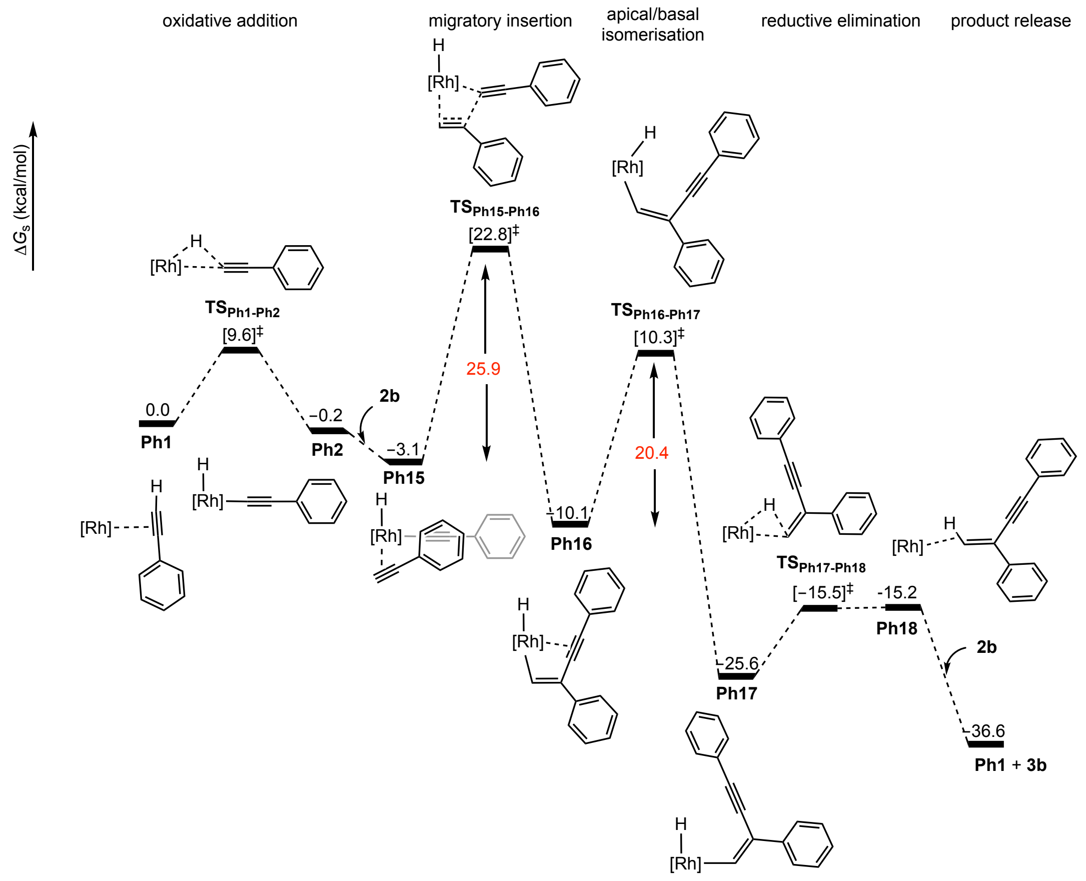

Figure S69: Calculated reaction profile (B3PW91-D3/SDD/6-31G**) for the head-to-tail homocoupling of **2b** affording **3b** via the carbometallation mechanism. Relative Gibbs free energies (kcal mol^{-1}) are corrected for DFB solvent.

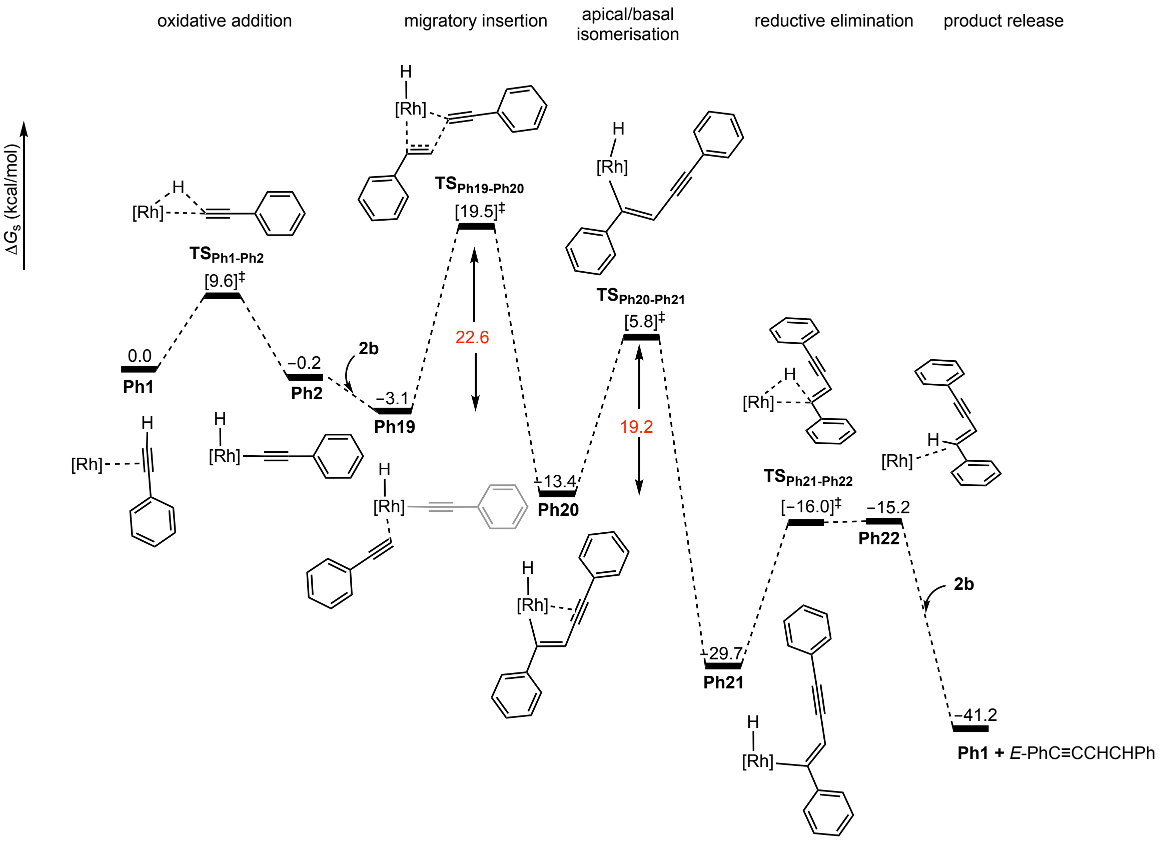

Figure S70: Calculated reaction profile (B3PW91-D3/SDD/6-31G**) for the head-to-head homocoupling of **2b** affording *E*-PhC≡CCHCHPh via the carbometallation mechanism. Relative Gibbs free energies (kcal mol^{-1}) are corrected for DFB solvent.

14. Homocoupling of *t*BuC≡CH (2j)

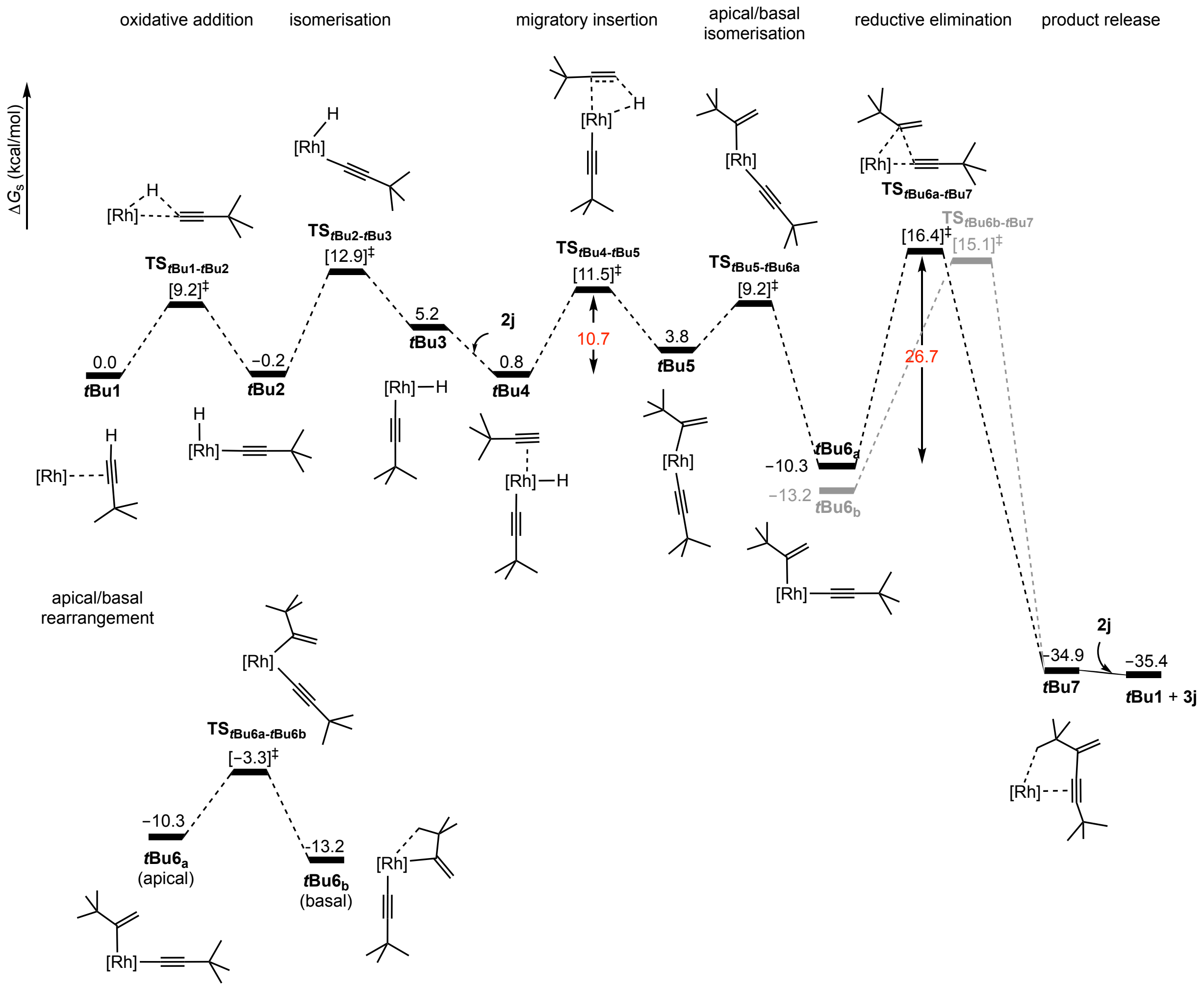

Figure S71: Calculated reaction profile (B3PW91-D3/SDD/6-31G**) for the head-to-tail homocoupling of **2j** affording **3j** via the hydrometallation mechanism. Relative Gibbs free energies (kcal mol^{-1}) are corrected for DFB solvent.

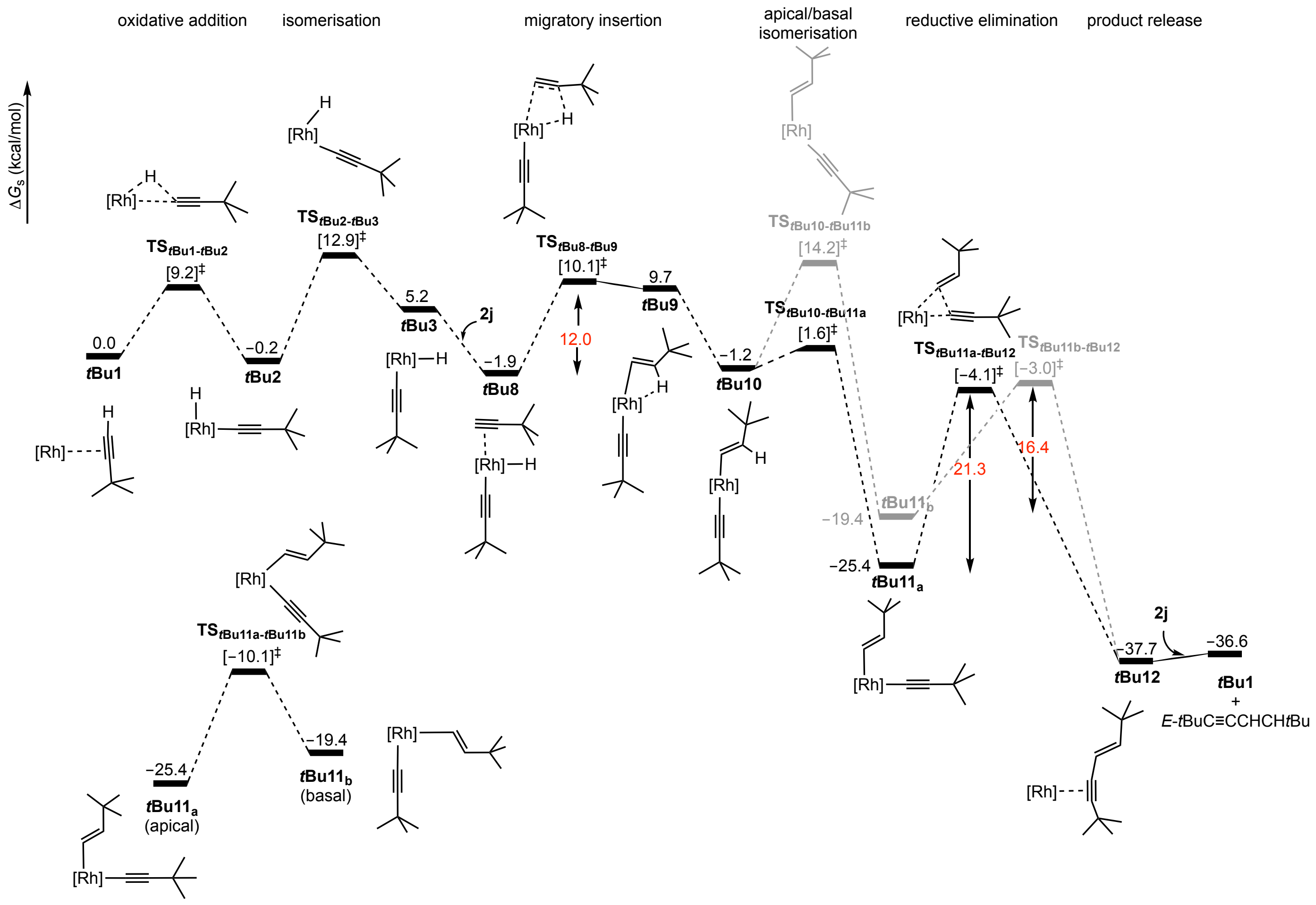

Figure S72: Calculated reaction profile (B3PW91-D3/SDD/6-31G**) for the head-to-head homocoupling of **2j** affording *E*-*t*BuC≡CCHCH*t*Bu via the hydrometallation mechanism. Relative Gibbs free energies (kcal mol^{-1}) are corrected for DFB solvent.

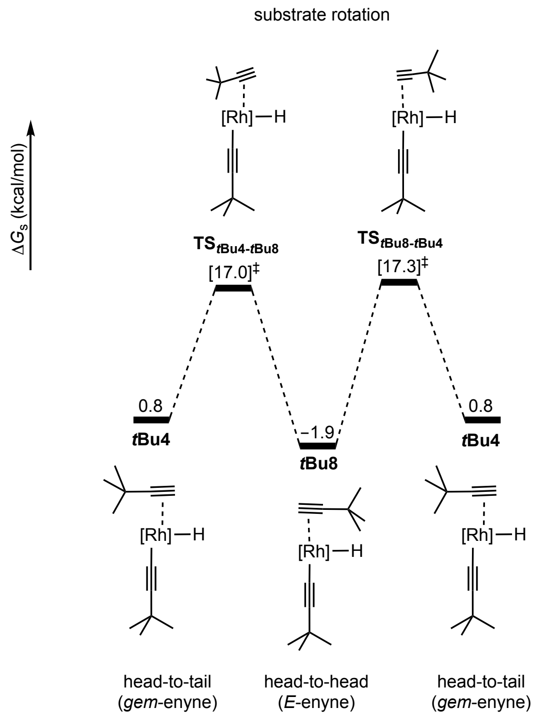

Figure S73: Calculated reaction profile (B3PW91-D3/SDD/6-31G**) for the interchange between ***t*Bu4** and ***t*Bu8** via substrate rotation, associated with the homocoupling of **2j**. Relative Gibbs free energies (kcal mol^{-1}) are corrected for DFB solvent.

15. Annulation of PhC≡CC(CH₂)Ph (3b)

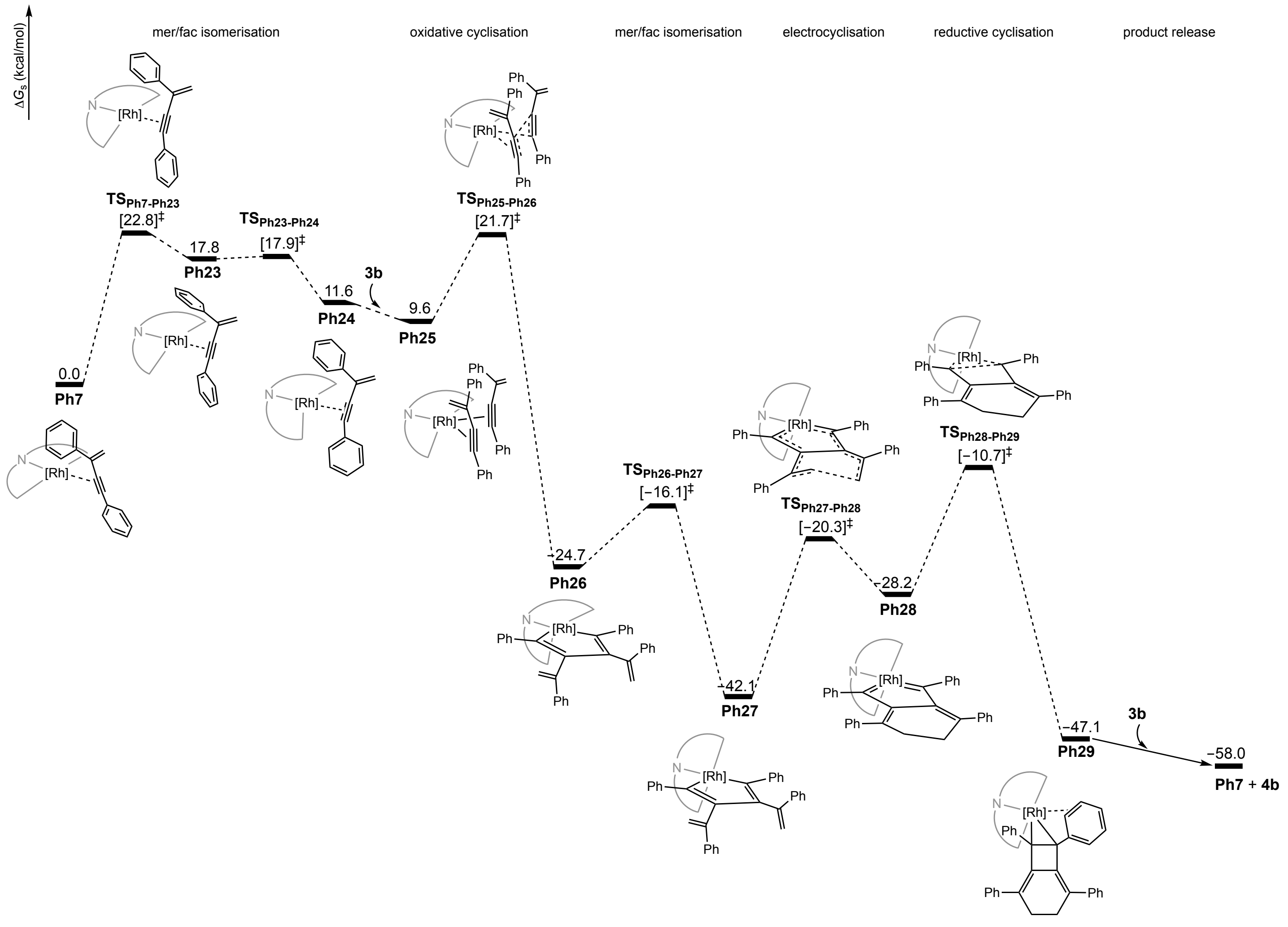

Figure S74: Calculated reaction profile (B3PW91-D3/SDD/6-31G**) for the lowest-energy zipper annulation pathway (via **Ph27**) affording **4b**. Relative Gibbs free energies (kcal mol⁻¹) are corrected for DFB solvent.

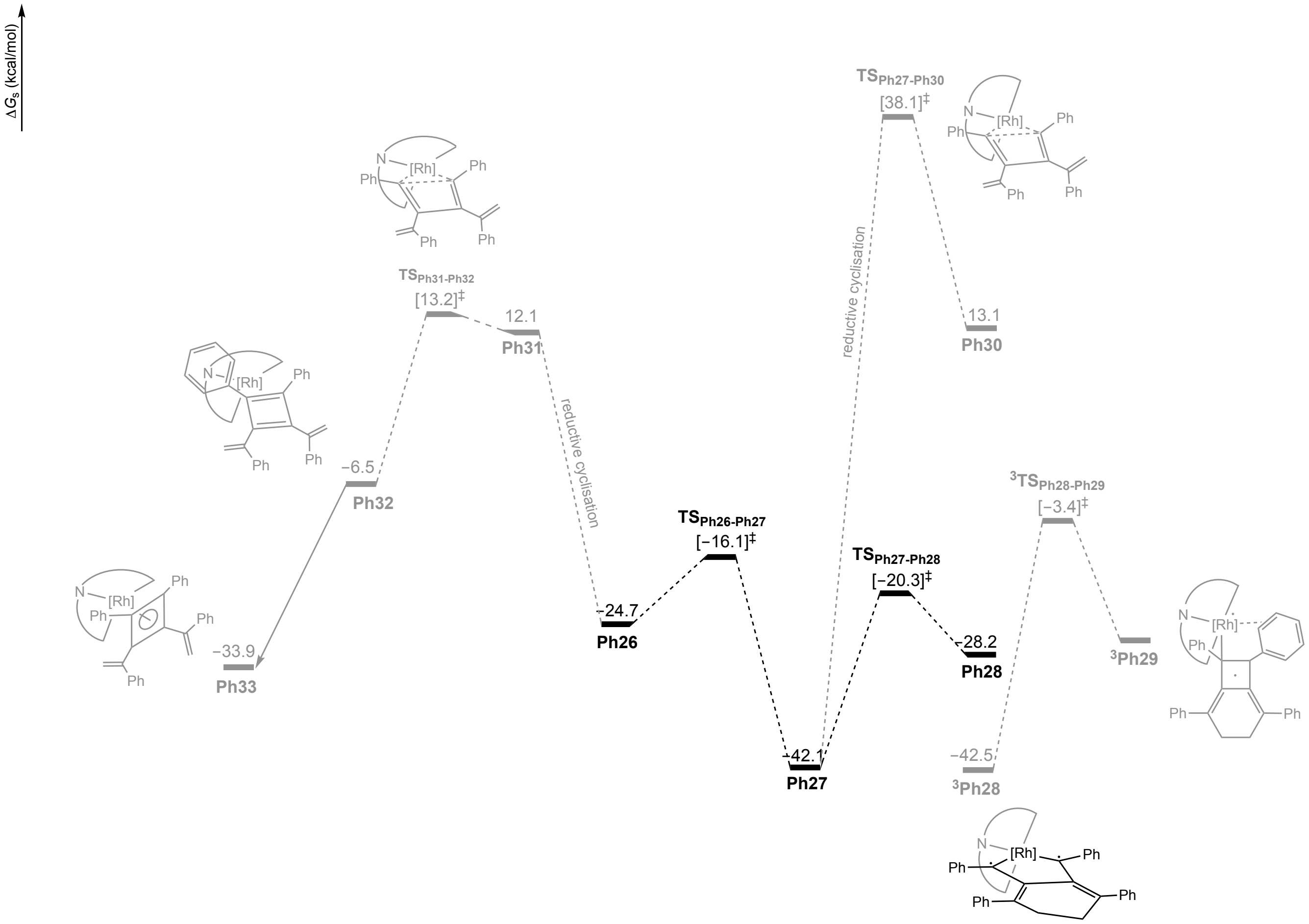

Figure S75: Calculated reaction profiles (B3PW91-D3/SDD/6-31G**) for alternative reaction pathways starting from **Ph26**. Relative Gibbs free energies (kcal mol^{-1}) are corrected for DFB solvent.

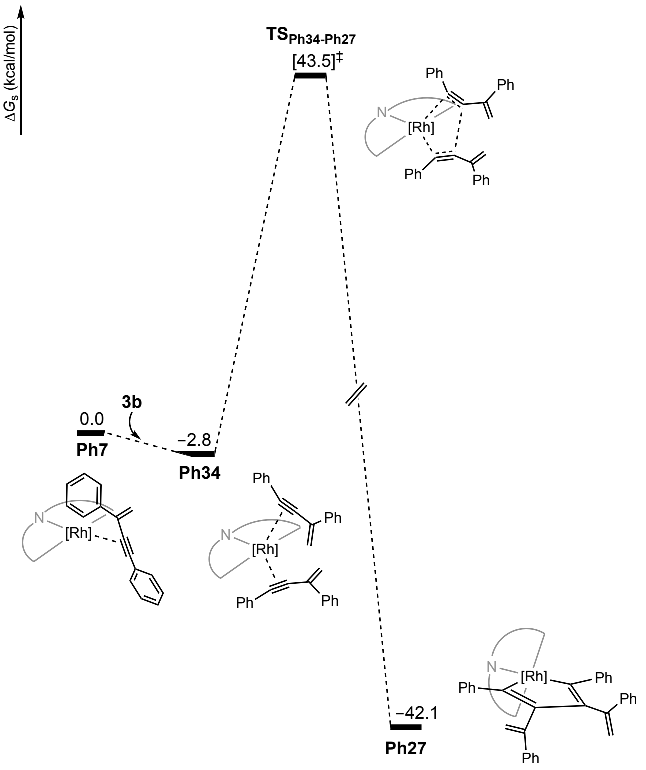

Figure S76: Calculated reaction profiles (B3PW91-D3/SDD/6-31G**) for an alternative reaction pathway starting from **Ph7**. Relative Gibbs free energies (kcal mol^{-1}) are corrected for DFB solvent.

16. References

[1] S. D. Pike, M. R. Crimmin and A. B. Chaplin, *Chem. Commun.*, 2017, **53**, 3615–3633.

[2] C. M. Storey, M. R. Gyton, R. E. Andrew and A. B. Chaplin, *Angew. Chem. Int. Ed.*, 2018, **57**, 12003–12006.

[3] R. Zhang, X. Hao, X. Li, Z. Zhou, J. Sun and R. Cao, *Cryst. Growth Des.* 2015, **15**, 2505–2513.

[4] C. Yang and S. P. Nolan, *J. Org. Chem.*, 2002, **67**, 591–593.

[5] S. Moulin, H. Dentel, A. Pagnoux-Ozherelyeva, S. Gaillard, A. Poater, L. Cavallo, J.-F. Lohier and J.-L. Renaud, *Chem. Eur. J.*, 2013, **19**, 17881–17890.

[6] R. E. Islas, J. Cárdenas, R. Gaviño, E. García-Ríos, L. Lomas-Romero and J. A. Morales-Serna, *RSC Adv.*, 2017, **7**, 9780–9789.

[7] S. Ventre, E. Derat, M. Amatore, C. Aubert and M. Petit, *Adv. Synth. Catal.*, 2013, **355**, 2584–2590.

[8] C. Lüken and C. Moberg, *Org. Lett.*, 2008, **10**, 2505–2508.

[9] C. Xu, W. Du, Y. Zeng, B. Dai and H. Guo, *Org. Lett.*, 2014, **16**, 948–951.

[10] C. Jahier, O. V. Zatolochnaya, N. V. Zvyagintsev, V. P. Ananikov and V. Gevorgyan, *Org. Lett.*, 2012, **14**, 2846–2849.

[11] Gaussian 09, Revision E.01, M. J. Frisch, G. W. Trucks, H. B. Schlegel, G. E. Scuseria, M. A. Robb, J. R. Cheeseman, G. Scalmani, V. Barone, B. Mennucci, G. A. Petersson, H. Nakatsuji, M. Caricato, X. Li, H. P. Hratchian, A. F. Izmaylov, J. Bloino, G. Zheng, J. L. Sonnenberg, M. Hada, M. Ehara, K. Toyota, R. Fukuda, J. Hasegawa, M. Ishida, T. Nakajima, Y. Honda, O. Kitao, H. Nakai, T. Vreven, J. A. Montgomery, Jr., J. E. Peralta, F. Ogliaro, M. Bearpark, J. J. Heyd, E. Brothers, K. N. Kudin, V. N. Staroverov, T. Keith, R. Kobayashi, J. Normand, K. Raghavachari, A. Rendell, J. C. Burant, S. S. Iyengar, J. Tomasi, M. Cossi, N. Rega, J. M. Millam, M. Klene, J. E. Knox, J. B. Cross, V. Bakken, C. Adamo, J. Jaramillo, R. Gomperts, R. E. Stratmann, O. Yazyev, A. J. Austin, R. Cammi, C. Pomelli, J. W. Ochterski, R. L. Martin, K. Morokuma, V. G. Zakrzewski, G. A. Voth, P. Salvador, J. J. Dannenberg, S. Dapprich, A. D. Daniels, O. Farkas, J. B. Foresman, J. V. Ortiz, J. Cioslowski and D. J. Fox, Gaussian, Inc., Wallingford CT, 2013.

[12] (a) A. D. Becke, *Phys. Rev. A*, 1988, **38**, 3098–3100; (b) A. D. Becke, *J. Chem. Phys.*, 1993, **98**, 5648–5652.

[13] D. Andrae, U. Häußermann, M. Dolg, H. Stoll and H. Preuß, *Theor. Chim. Acta.*, 1990, **77**, 123–141.

[14] A. W. Ehlers, M. Böhme, S. Dapprich, A. Gobbi, A. Höllwarth, V. Jonas, K. F. Köhler, R. Stegmann, A. Veldkamp and G. Frenking, *Chem. Phys. Lett.*, 1993, **208**, 111–114.

[15] (a) W. J. Hehre, R. Ditchfield and J. A. Pople, *J. Chem. Phys.*, 1972, **56**, 2257–2261. (b) P. C. Harihara and J. A. Pople, *Theor. Chim. Acta.*, 1973, **28**, 213–222.

[16] S. Grimme, J. Antony, S. Ehrlich and H. Krieg, *J. Chem. Phys.*, 2010, **132**, 154104.

[17] S. Grimme, S. Ehrlich and L. Goerigk, *J. Comp. Chem.*, 2011, **32**, 1456–1465.

[18] A. V. Marenich, C. J. Cramer and D. G. Truhlar, *J. Phys. Chem. B*, 2009, **113**, 6378–6396.

[19] G. A. Zhurko, D. A. Zhurko and G. A. Andrienko, Chemcraft Version 1.8 (Build 536).
